# Supplementary material for: The lysophospholipase D enzyme Gdpd3 is required to maintain chronic myelogenous leukaemia stem cells
Source: Nat Commun. 2020 Sep 17;11:4681. doi: 10.1038/s41467-020-18491-9 (PMC7499193; doi:10.1038/s41467-020-18491-9)
Supplement: Supplementary file 1 — Supplementary Information [file 41467_2020_18491_MOESM1_ESM.pdf]

Supplementary Information PDF

The lysophospholipase D enzyme Gdpd3 is required to maintain chronic myelogenous leukaemia stem cells

Kazuhito Naka, Ryosuke Ochiai, Eriko Matsubara, Chie Kondo, Kyung-Min Yang, Takayuki Hoshii, Masatake Araki, Kimi Araki, Yusuke Sotomaru, Ko Sasaki, Kinuko Mitani, Dong-Wook Kim, Akira Ooshima and Seong-Jin Kim

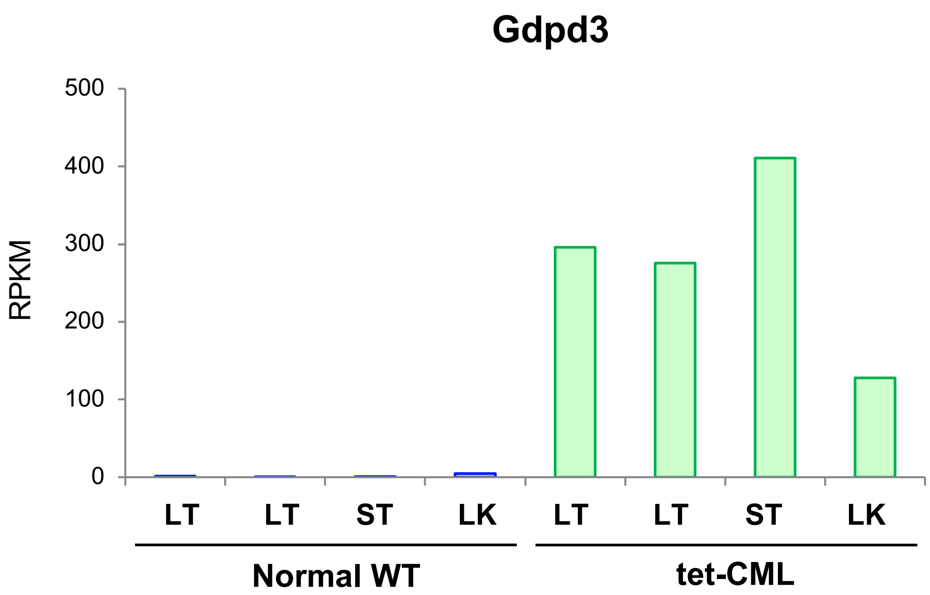

Supplementary Figure 1. Expression of Gdpd3 mRNA as determined by RNA-sequencing.

RNA-Seq determinations of *Gdpd3* mRNA levels in LT-stem cells, ST-stem cells and Lineage$^-$Sca-1$^-$cKit$^+$ (LK) cell populations isolated from bone marrow mononuclear cells (BMMNCs) of littermate normal healthy control mice (*SCL-tTA*$^+$) and tet-CML mice (*SCL-tTA*$^+$*TRE-BCR-ABL1*$^+$*)* determined as reported previously[26]. Results are expressed as RPKM (reads per kilobase of exon per million mapped sequence reads) from a public database gene expression omnibus that we previously made available (GEO, ID: GSE70031, NCBI, NIH, USA). Source data are available in Source Data file.

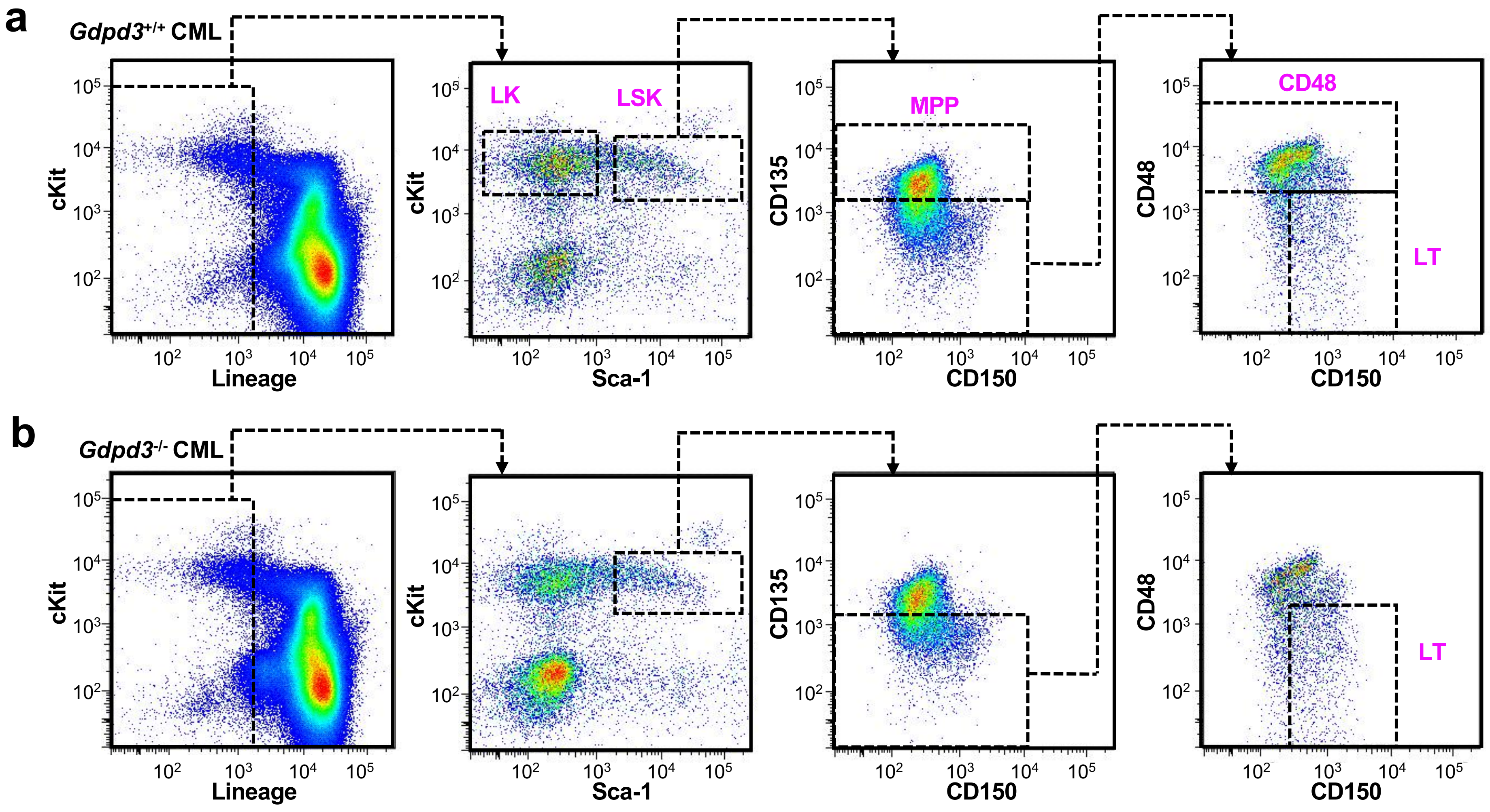

Supplementary Figure 2. Fractionation of sub-populations of BMMNCs isolated from *Gdpd3*$^{+/+}$ and *Gdpd3*$^{-/-}$ tet-CML-affected mice. Total BMMNCs were isolated from the two hind limbs of (**a**) *Gdpd3*$^{+/+}$ tet-CML-affected and (**b**) *Gdpd3*$^{-/-}$ tet-CML-affected mice. These cells were immunostained to detect lineage markers (CD4, CD8, B220, Mac1, Gr-1 and Ter119), as well as Sca-1, cKit, CD135, CD48 and CD150, and analysed by flow cytometry. The gating pattern for cell sorting and representative flow cytometric data of LK cells (Lineage^{-}Sca-1^{-}cKit^{+}), LSK cells (Lineage^{-}Sca-1^{+}cKit^{+}), MPP cells (CD135^{+}LSK), CD48 cells (CD48^{+}CD135^{-}LSK), and the most primitive LT-CML stem cells (CD150^{+}CD48^{-}CD135^{-}LSK) are shown.

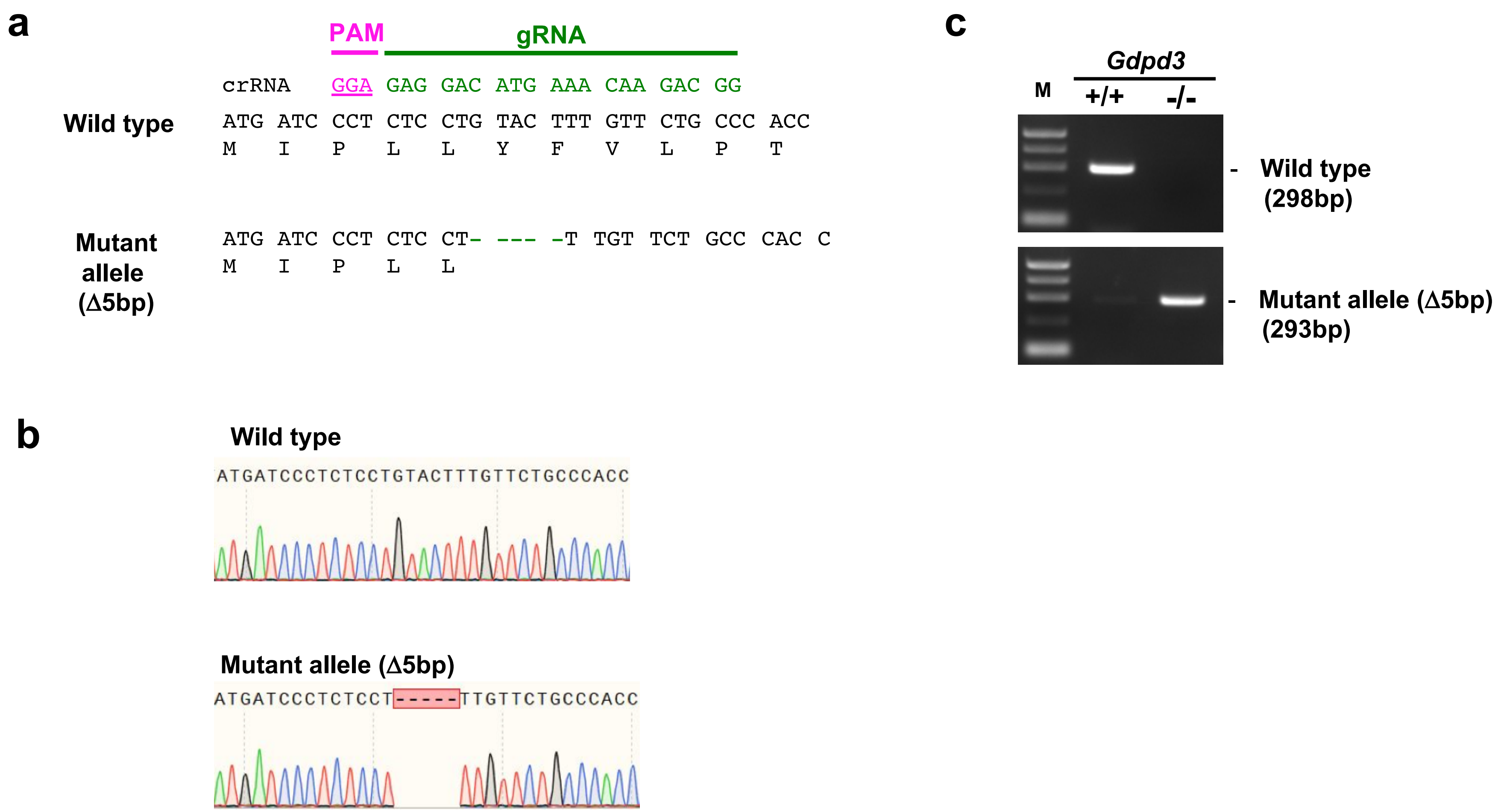

Supplementary Figure 3. Establishment of *Gdpd3*$^{-/-}$ mice by genome-editing.

(**a**) Nucleotide and amino acid sequences of the *Gdpd3* gene in *Gdpd3*$^{+/+}$ (upper) and 5 bp-disrupted *Gdpd3*$^{-/-}$ (lower) mice are indicated. The targetting gRNA and PAM sequences are also indicated. (**b,c**) Identification of genome-edited mice. (**b**) Representative genomic sequencing data (n=5 biologically independent samples, two independent experiments) and (**c**) PCR genotyping (n=5 biologically independent samples, three independent experiments) of *Gdpd3*$^{+/+}$ and *Gdpd3*$^{-/-}$ mouse tails are shown. M, molecular weight markers. Unprocessed original gel in (**c**) is available in Source Data file.

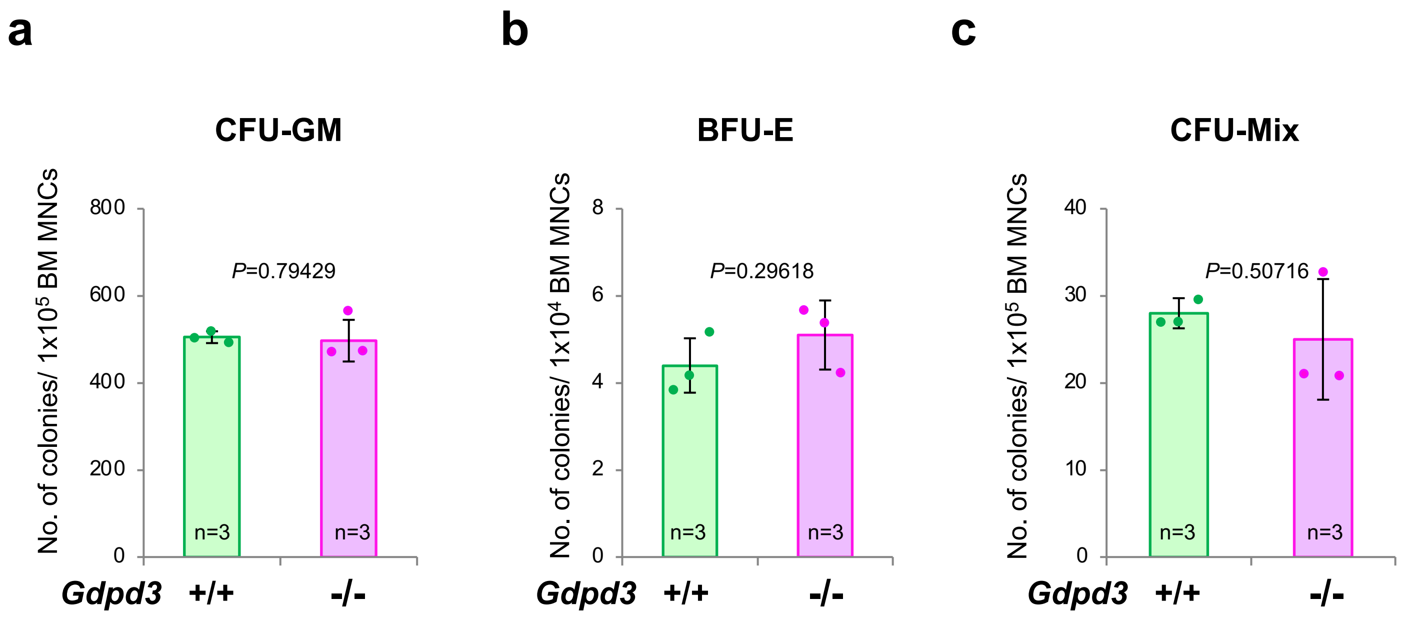

Supplementary Figure 4. Colony-forming capacity of normal murine haematopoietic cells *in vitro*.

BMMNCs isolated from normal healthy *Gdpd3*$^{+/+}$ and *Gdpd3*$^{-/-}$ littermate mice were cultured with cytokines in semi-solid methylcellulose medium for seven days under hypoxic (3% O_2) conditions at 37° C. Data are the mean number±s.d. (n=3 biologically independent samples) of colonies for (**a**) myeloid (CFU-GM), (**b**) erythroid (BFU-E) and (**c**) mixed (CFU-mix) lineages, and are representative of three independent experiments (*P*-value compared with control, unpaired two-sided Student's t-test). Source data are available in Source Data file.

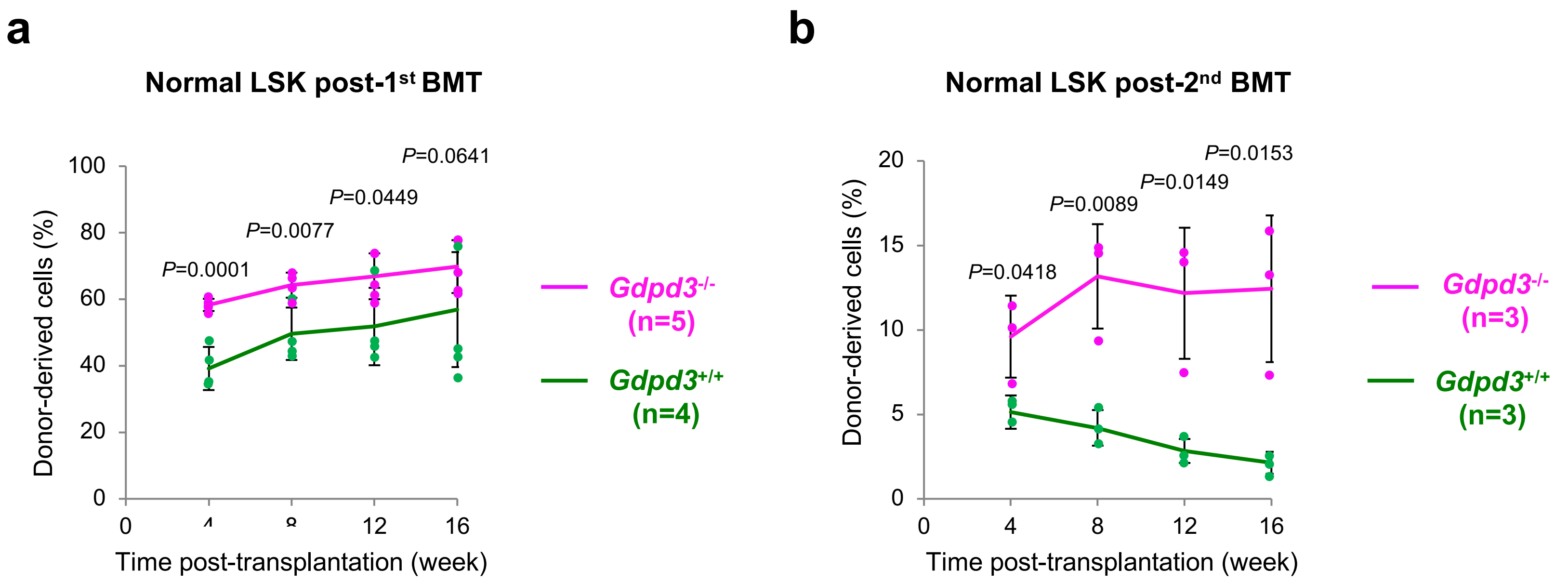

Supplementary Figure 5. Competitive bone marrow reconstitution assays using normal haematopoietic stem/progenitor cells *in vivo*. **(a)** Lethally irradiated C57BL/6-CD45.1 congenic recipient mice were transplanted with normal haematopoietic LSK cells (2,000 cells) isolated from normal healthy *Gdpd3*$^{+/+}$ or *Gdpd3*$^{-/-}$ littermate mice (C57BL/6-CD45.2) plus 5x10^{5} unfractionated BMMNCs from healthy C57BL/6-CD45.1 mice. Reconstitution of donor-derived cells (CD45.2) was monitored by staining blood cells with mAbs against CD45.2 (FITC) and CD45.1 (PE). **(b)** For serial transplantation analyses, normal LSK cells (2,000 cells) were obtained from recipient mice at sixteen weeks post-transplantation (first-BMT) and transplanted into a second set of lethally irradiated C57BL/6-CD45.1 congenic mice (second-BMT) using the same protocol as for the first-BMT. **(a,b)** Results are the mean frequency (%)±s.d. of donor-derived cells (*P*-value, unpaired two-sided Student's t-test). n numbers indicate biologically independent recipient mouse numbers. Source data are available in Source Data file.

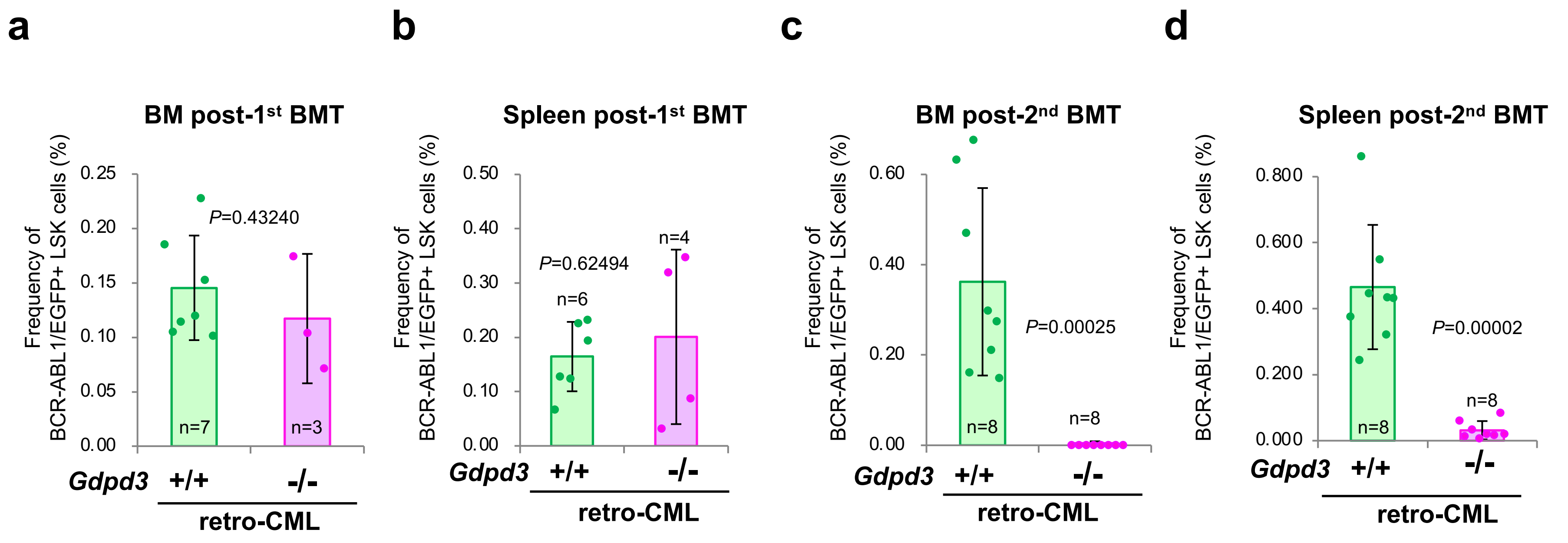

Supplementary Figure 6. Frequency of BCR-ABL1/EGFP^{+}CML LSK cells in *Gdpd3*$^{+/+}$ and *Gdpd3*$^{-/-}$ retro-CML-affected mice. BCR-ABL1/EGFP^{+}CML LSK cells were isolated from **(a,c)** BM of the two hind limbs and **(b,d)** spleen of *Gdpd3*$^{+/+}$ retro-CML-affected mice and *Gdpd3*$^{-/-}$ retro-CML-affected mice after **(a,b)** first-round and **(c,d)** second-round serial transplantation. Frequency of BCR-ABL1/EGFP^{+} LSK cells were examined by a FACS Aria III cell sorter with BD FACSDiva software ver.6.1.3 (BD Biosciences) Data are the mean frequency (%)±s.d. of BCR-ABL1/EGFP^{+} LSK cells (*P*-value, unpaired two-sided Student's t-test). (See also Fig.2c-f). n numbers indicate biologically independent recipient mouse numbers. Source data are available in Source Data file.

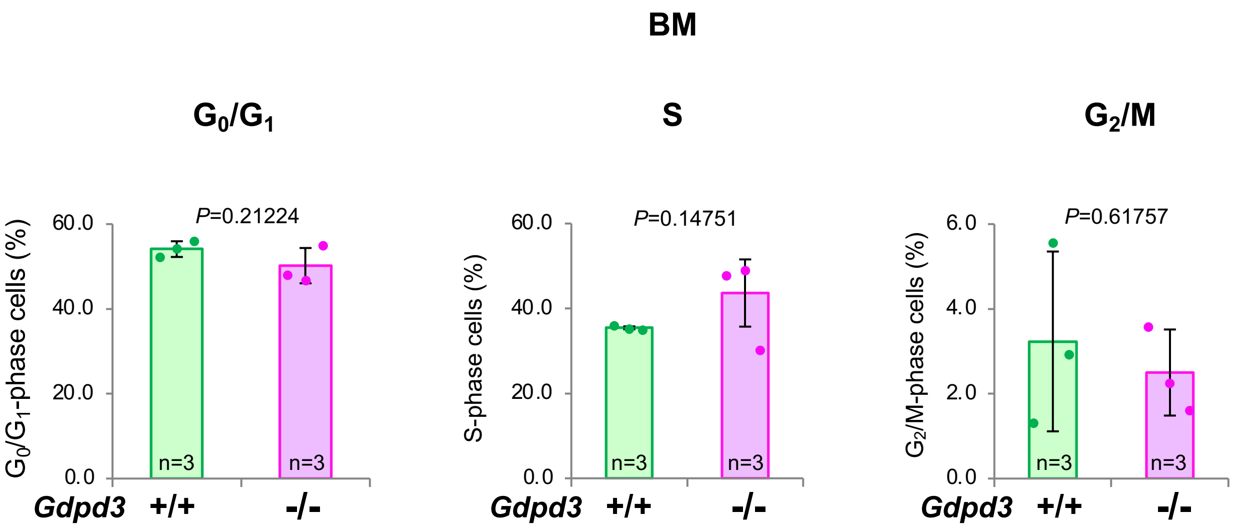

Supplementary Figure 7. Cell cycle distribution of BCR-ABL1/EGFP$^+$CML LSK cells in bone marrow of retro-CML-affected mice. *Gdpd3*$^{+/+}$ retro-CML-affected mice (3 females) and *Gdpd3*$^{-/-}$ retro-CML-affected mice (3 females) were intraperitoneally administered BrdU (100 mg kg^{-1} of body weight in saline; Sigma) for three hrs after a first-round of BMT as in Fig.2a. BCR-ABL1/EGFP$^+$CML LSK cells were isolated from bone marrow of two hind limbs of retro-CML-affected and stained with anti-BrdU antibody (3D4; BD Biosciences) plus 7AAD (BD Biosciences). Frequency of BrdU$^+$ CML LSK cells in bone marrow was evaluated by a FACS Aria III cell sorter with BD FACSDiva software ver.6.1.3 (BD Biosciences). Results are the mean frequency (%)±s.d. of G_0/G_1 phase CML-LSK cells, BrdU$^+$ S-phase CML-LSK cells, and G_2/M phase CML-LSK cells (n=3 biologically independent samples) (*P*-value, unpaired two-sided Student's t-test) (See also Fig.2,g,h). Source data are available in Source Data file.

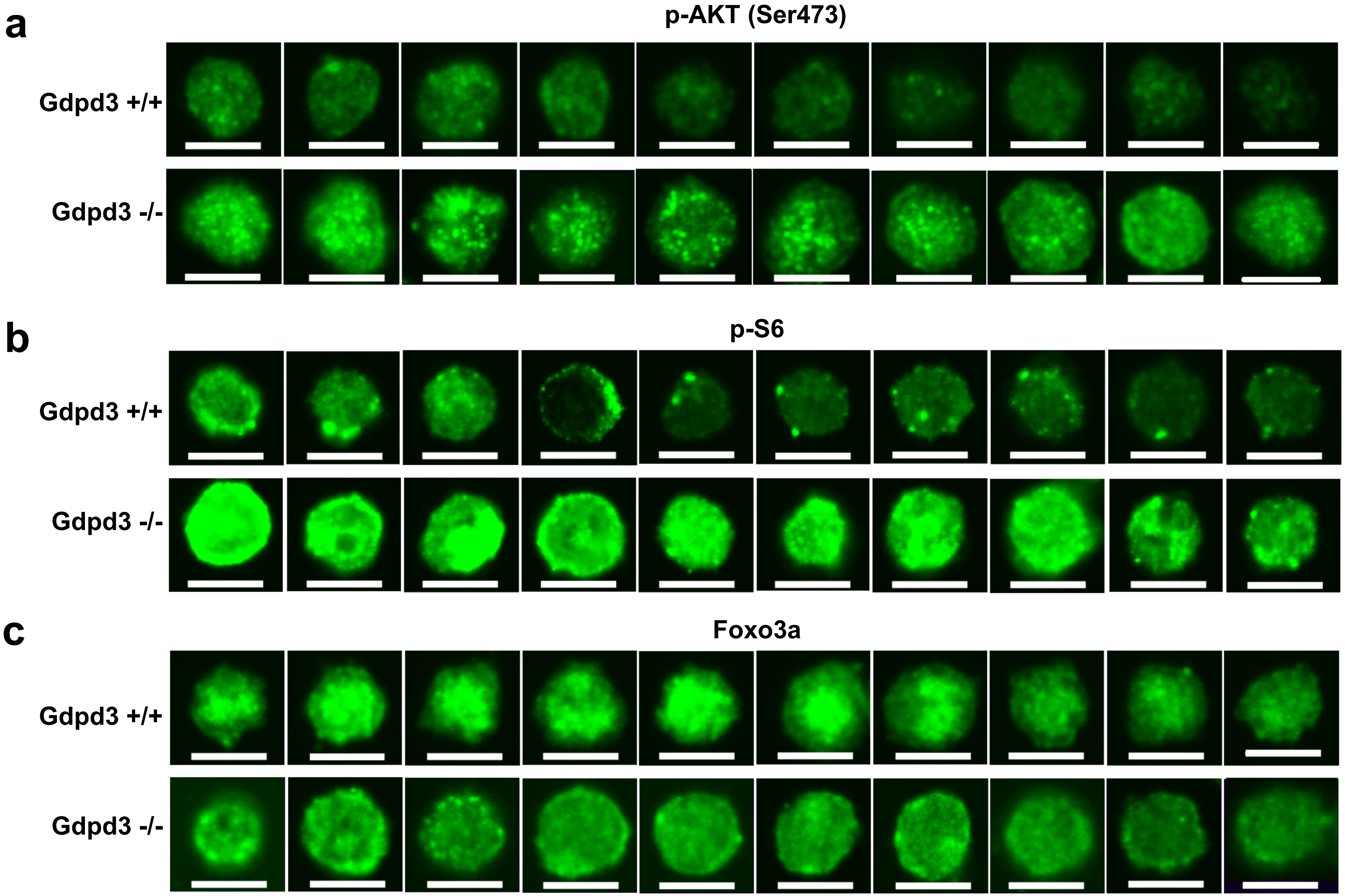

Supplementary Figure 8. Increased phosphorylation of AKT and S6 ribosomal protein and decreased nuclear localisation of Foxo3a in *Gdpd3*$^{-/-}$ LT-CML stem cells. LT-CML stem cells isolated from *Gdpd3*$^{+/+}$ tet-CML-affected mice (4 males), or *Gdpd3*$^{-/-}$ tet-CML-affected mice (3 males), at five weeks post-Dox withdrawal were immunostained to detect (**a**) phosphorylated AKT (Ser473), (**b**) phosphorylated S6 ribosomal protein (Ser235/236), and (**c**) Foxo3a. Scale bar, 10μm. Results are representative of three biologically independent trials. (See also Fig.5).

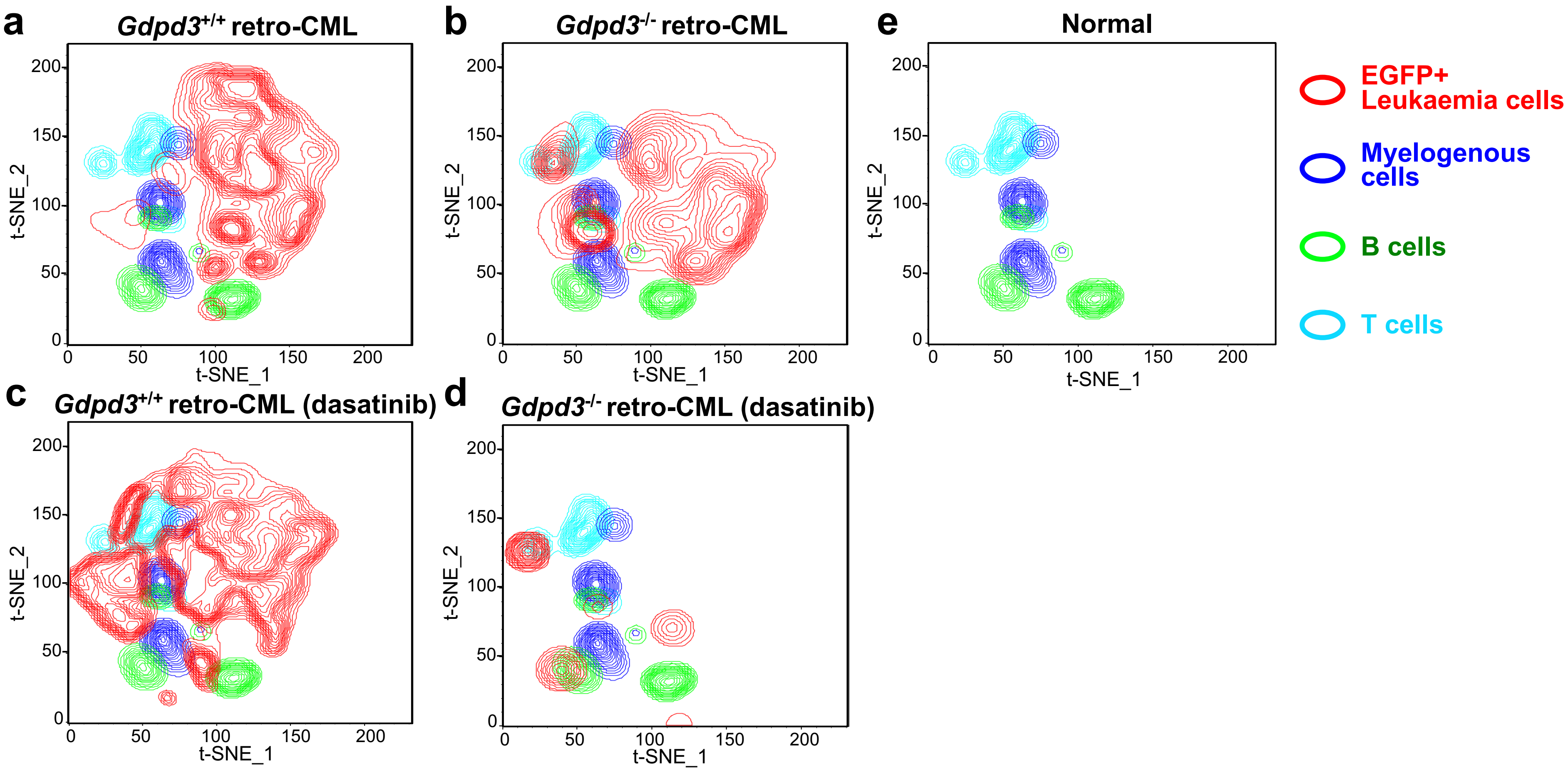

Supplementary Figure 9. t-SNE analyses of cell populations in peripheral blood (PB) of retro-CML-affected mice.

(a-d) Representative t-SNE analyses of BCR-ABL1/EGFP$^+$ leukaemia cells in PB from (**a,b**) untreated *Gdpd3*$^{+/+}$ and *Gdpd3*$^{-/-}$ retro-CML-affected mice (as in Fig.6b), and (**c,d**) dasatinib-treated *Gdpd3*$^{+/+}$ and *Gdpd3*$^{-/-}$ retro-CML-affected mice (as in Fig.6c). (**e**) Representative t-SNE analysis of PB mononuclear cells (MNCs) from one healthy WT C57BL/6 mouse (8-wk-old female; negative control). PB MNCs were stained with anti-CD4 and anti-CD8 (PE-Cy7), anti-B220 (PE), anti-Mac1 and anti-Gr-1 (APC) mAbs followed by flow cytometry. t-SNE algorithm analyses were performed using FlowJo^{TM} (build number 10.6.1) software. For (**a-d**), the red contour lines indicating BCR-ABL1/EGFP$^+$ leukaemia cells were overlayed on the t-SNE data for the PB MNCs from the normal C57BL/6 mouse in (**e**). The blue contour lines represent Mac1/Gr-1$^+$ myelogenous cells. The green contour lines represent B220$^+$ B cells. The light blue contour lines represent CD4$^+$/CD8$^+$ T cells. Results are representative of three biologically independent trials (n=4 biologically independent samples).

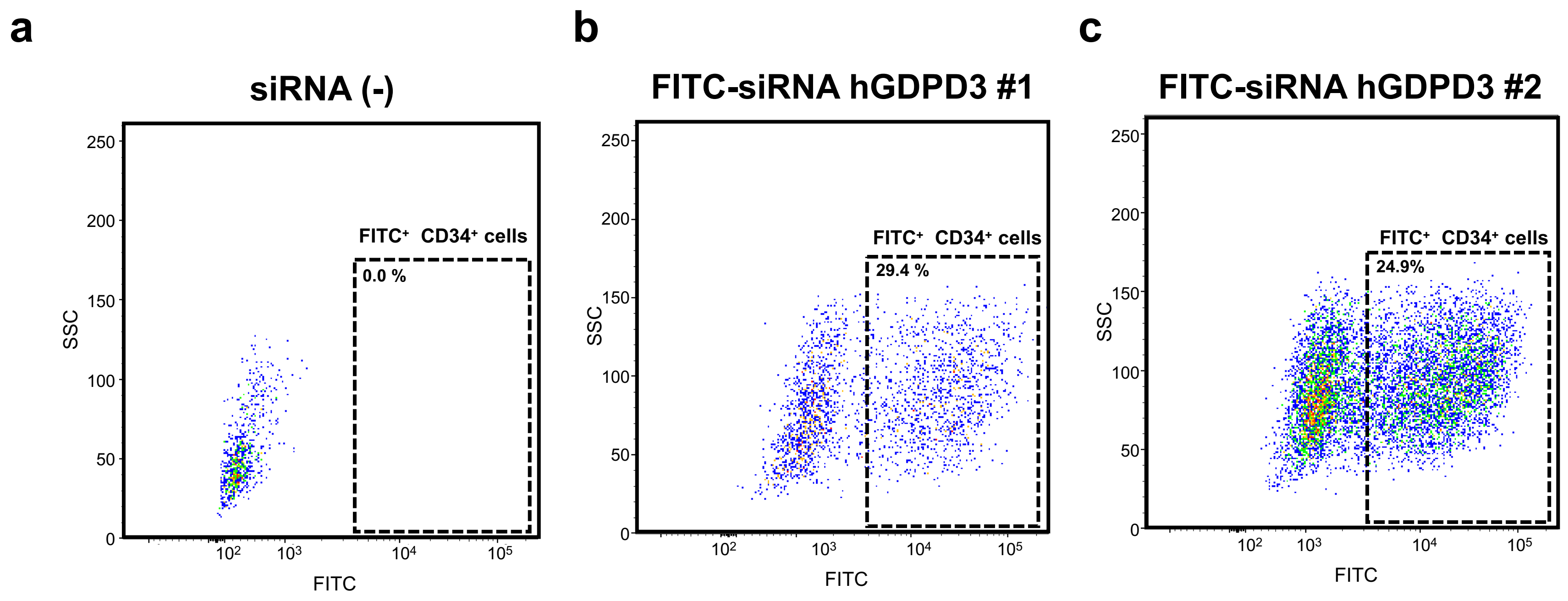

Supplementary Figure 10. Purification of human bone marrow CML CD34$^+$ cells transduced with FITC-labelled hGDPD3 siRNAs. Human CML CD34$^+$ cells isolated from BM of a chronic phase CML patient were (**a**) mock-transduced, or (**b**) transduced with FITC-siRNA hGDPD3 #1 or (**c**) FITC-siRNA hGDPD3 #2 (both targetting human GDPD3 mRNA; see Methods). FITC$^+$CML CD34$^+$ cells and FITC$^-$CML CD34$^+$ cells were purified at three days post-transduction by cell sorter using a FACS Aria III instrument (BD Biosciences). The colony-forming capacity of these FITC$^-$ and FITC$^+$ human CML CD34$^+$ cells was determined by culture on semi-solid methylcellulose medium (Methocult GF$^+$ H4435; Stem Cell Technologies) under hypoxic (3% O_2) conditions as in Fig.6f,g.

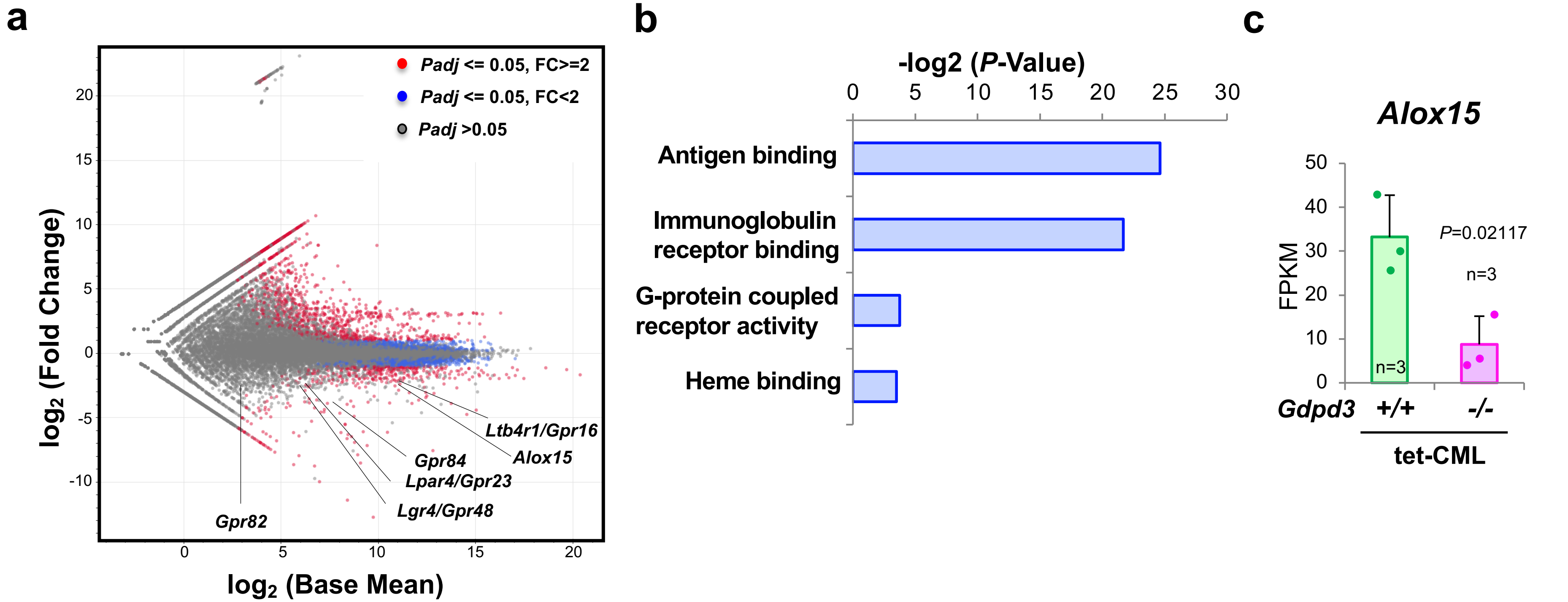

Supplementary Figure 11. Bioinformatics analyses of LT CML stem cells isolated from *Gdpd3*$^{+/+}$ and *Gdpd3*$^{-/-}$ tet-CML-affected mice. (**a**,**b**) Results from RNA-Seq analyses were evaluated by bioinformatics using (**a**) MA-plot and (**b**) GO term enrichment analyses. RNAs were extracted from *Gdpd3*$^{+/+}$ tet-CML-affected mice (22 males, 12 females) and *Gdpd3*$^{-/-}$ tet-CML-affected mice (5 male, 5 females). RNA-Seq was performed as described in Methods. (**a**) FPKM, log2 (BaseMean), log2 (FoldChange), *P*-value (two-tailed Wald test)[52], and adjusted *P*-value (two-tailed Wald test)[52,53] were determined by the Bioconductor package DESeq2 (ver. 1.20.0) (https://bioconductor.org/packages/release/bioc/html/DES0eq2.html)[52]. MA-plot indicating genes in the Bokeh library (ver. 0.13.0) (https://docs.bokeh.org/en/0.13.0/) that were upregulated or downregulated in *Gdpd3*$^{-/-}$ tet-CML-affected mice compared to *Gdpd3*$^{+/+}$ tet-CML-affected mice. X-axis, $\log_2$ base mean. Y-axis, $\log_2$ fold change. (**b**) GO term enrichment analysis categorised by MF (molecular function) using the DAVID Bioinformatics Resource 6.8. (http://david.abcc.ncifcrf.gov). Genes were filtered according to the criterion that the FPKM (fragments per kilobase of exon per million reads mapped) values obtained exhibited less than a -1.5 fold change in *Gdpd3*$^{-/-}$ tet-CML-affected mice compared to values in *Gdpd3*$^{+/+}$ tet-CML-affected mice. X-axis, $-\log_2$ *P*-value. (**c**) FPKM determination of *Alox15* expression in LT CML stem cells from *Gdpd3*$^{+/+}$ tet-CML-affected mice and *Gdpd3*$^{-/-}$ tet-CML-affected mice. Data are the mean FPKM±s.d. (n=3 biologically independent samples) (*P*-value, unpaired two-sided Student's t-test). Source data are available in Source Data file.

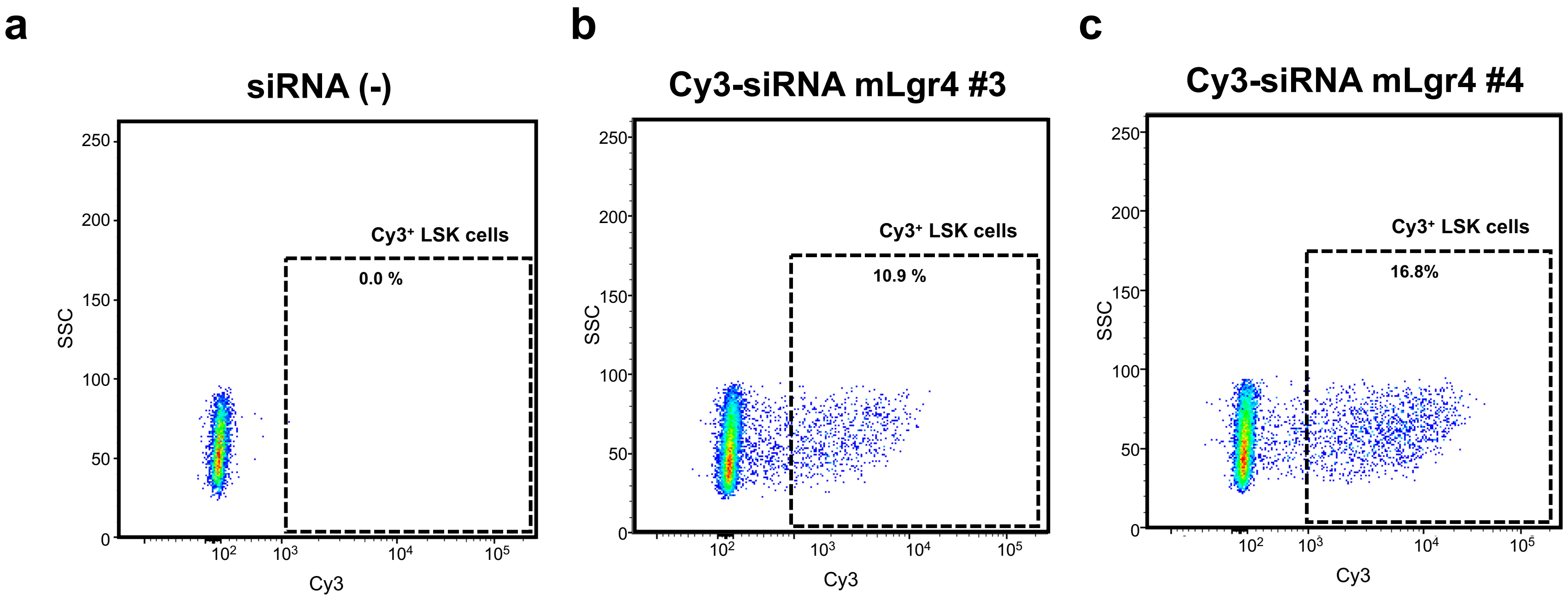

Supplementary Figure 12. Purification of CML LSK cells transduced with Cy3-labelled Lgr4 siRNA.

CML LSK cells isolated from *Gdpd3*$^{+/+}$ tet-CML-affected mice (3 males) were (**a**) mock-transduced, or (**b**) transduced with Cy3-siRNA mLgr4 #3 or (**c**) Cy3-siRNA mLgr4 #4 (both targetting mouse *Lgr4*/*Gpr48* mRNA; see Methods). Cy3^{+} CML LSK cells and Cy3^{-} CML LSK cells were purified at three days post-transduction by cell sorter using a FACS Aria III instrument (BD Biosciences). In Fig.7b, the colony-forming capacity of these Cy3^{-} and Cy3^{+} CML LSK cells was determined by culture on semi-solid methylcellulose medium under hypoxic (3% O_2) conditions.

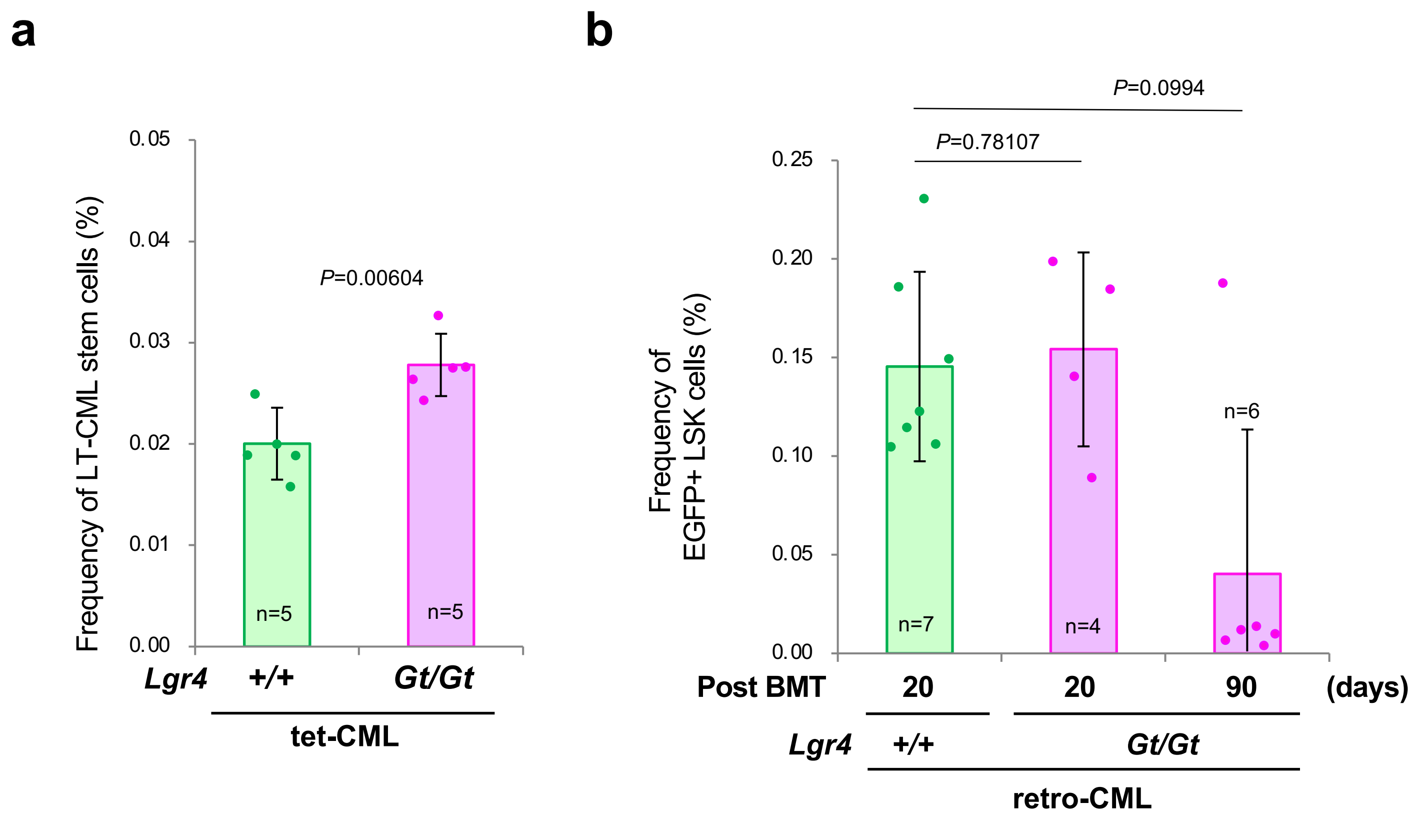

Supplementary Figure 13. Frequency of primitive CML stem/progenitor cells in *Lgr4*$^{+/+}$ and *Lgr4*$^{Gt/Gt}$ CML-affected mice.

(a) Frequency of LT-CML stem cells isolated from BM of the two hind limbs of *Lgr4*$^{+/+}$ tet-CML-affected mice (4 males, 1 female) and *Lgr4*$^{Gt/Gt}$ tet-CML-affected mice (5 females). Data are the mean frequency (%)±s.d. of LT-CML stem cells (n=5 biologically independent samples) (*P*-value, unpaired two-sided Student's t-test). (See Figure 7c). **(b)** Frequency of BCR-ABL1/EGFP^{+}CML LSK cells isolated from BM of the two hind limbs of *Lgr4*$^{+/+}$ retro-CML-affected mice and *Lgr4*$^{Gt/Gt}$ retro-CML-affected mice after a first-round of transplantation. Data are the mean frequency (%)±s.d. of BCR-ABL1/EGFP^{+} LSK cells (n numbers indicate biologically independent sample numbers) (*P*-value, unpaired two-sided Student's t-test). (See also Fig.7f). Source data are available in Source Data file.

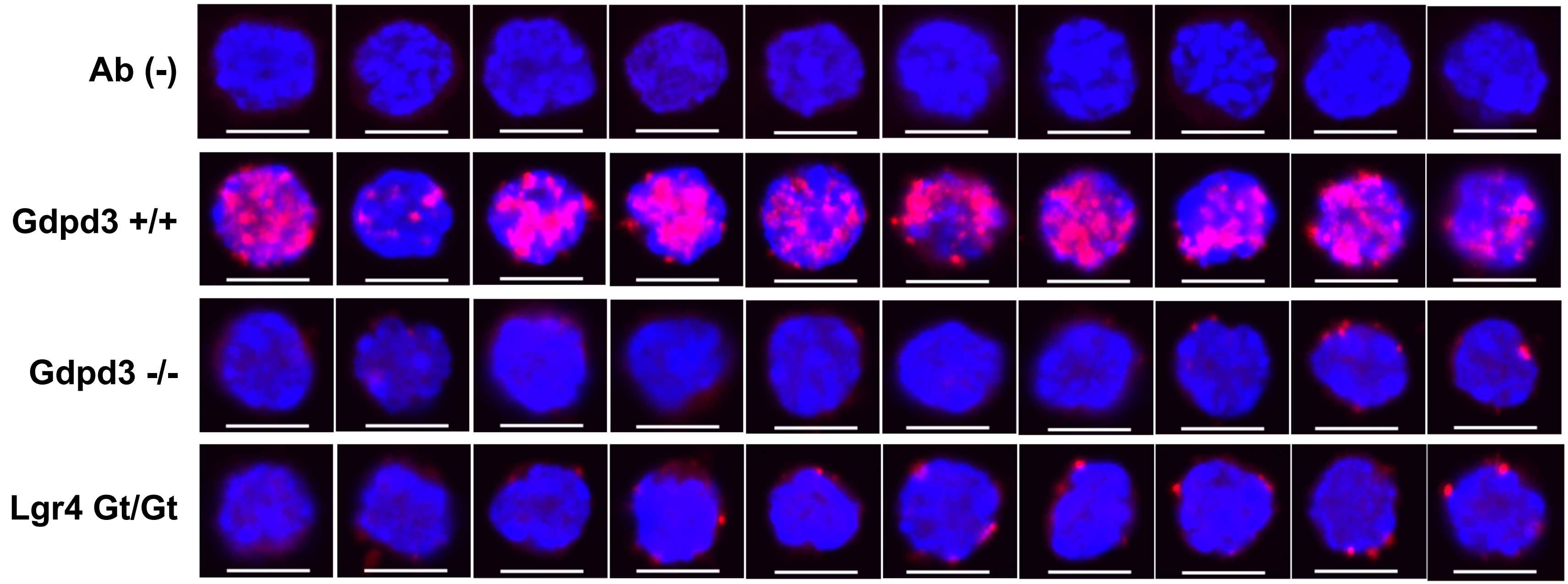

Supplementary Figure 14. Duolink® *in situ* PLA to detect Foxo3 binding to β-catenin in freshly isolated LT-CML stem cells. Freshly isolated LT-CML stem cells from *Gdpd3*$^{+/+}$ tet-CML-affected mice (4 males, 2 females), *Gdpd3*$^{-/-}$ tet-CML-affected mice (3 males, 2 females), or *Lgr4*$^{Gt/Gt}$ tet-CML-affected mice (4 females), at five weeks post-Dox withdrawal were subjected to Duolink® *in situ* PLA imaging (Merck) using rabbit anti-Foxo3a and mouse anti-active β-catenin monoclonal Abs as in **Fig.8a**. Results are representative of three biologically independent trials. Ab (-), technical negative control without primary antibody. Nuclei were visualised using DAPI. Scale bar, 10μm.

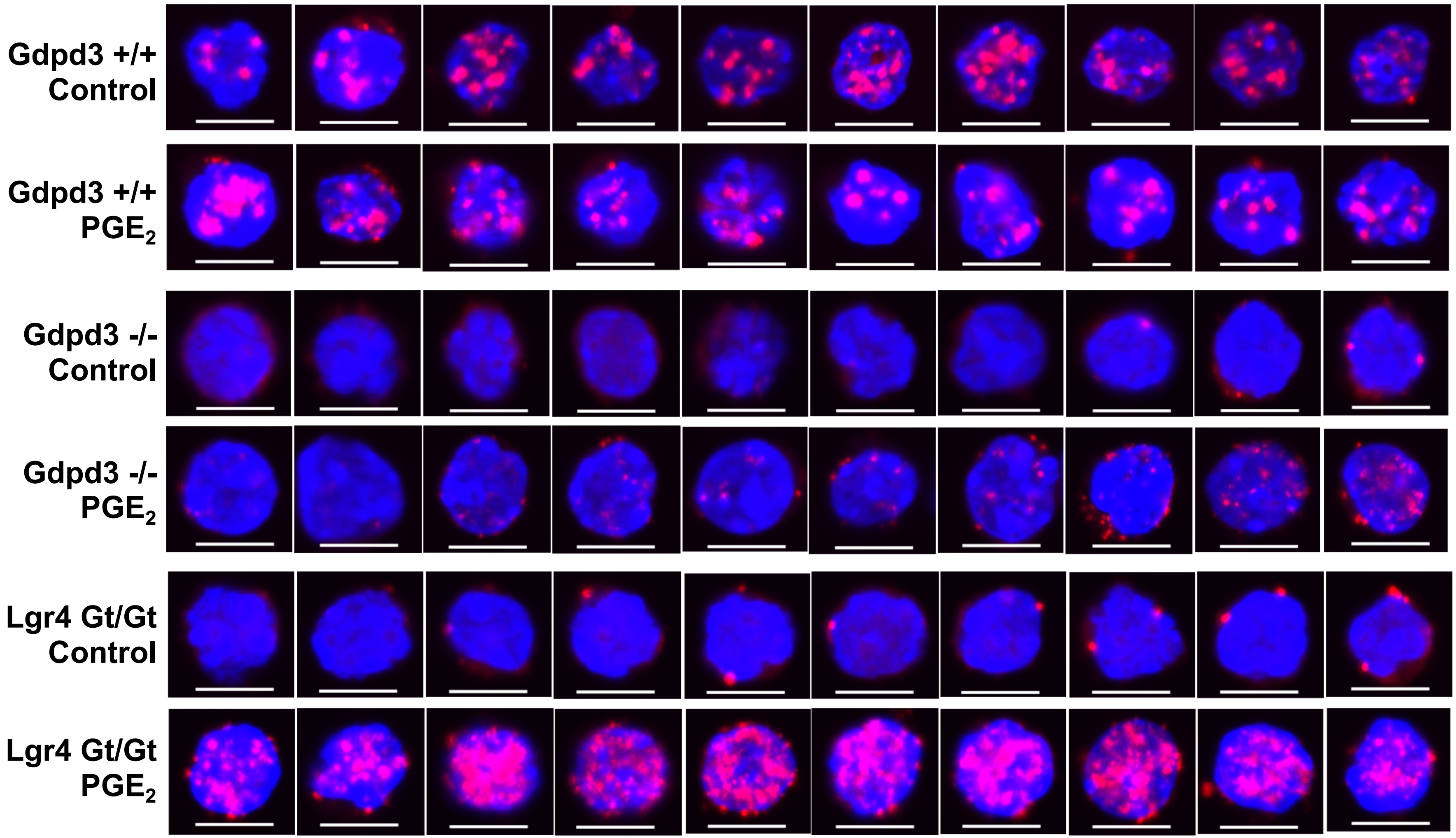

Supplementary Figure 15. Duolink® *in situ* PLA to detect Foxo3 binding to β-catenin in PGE_2-treated LT-CML stem cells.

LT-CML stem cells isolated from *Gdpd3*$^{+/+}$ tet-CML-affected mice (2 females), *Gdpd3*$^{-/-}$ tet-CML-affected mice (2 females), or *Lgr4*$^{Gt/Gt}$ tet-CML-affected mice (2 females), at five weeks post-Dox withdrawal were treated *in vitro* with/without PGE_2 (10μM) for two hours. These LT-CML stem cells were subjected to Duolink® *in situ* PLA imaging (Merck) using rabbit anti-Foxo3a and mouse anti-active β-catenin monoclonal Abs as in **Fig.8b**. Results are representative of two biologically independent trials. Nuclei were visualised using DAPI.

Scale bar, 10μm.

Supplementary Table 1. Blood Cell Counts in *Gdpd3*$^{+/+}$ and *Gdpd3*$^{-/-}$ Mice

Genotype	*Gdpd3*$^{+/+}$	*Gdpd3*$^{-/-}$	*Gdpd3*$^{-/-}$
Age (week-old)	10	10	39
N	10	10	10
Male	5	5	5
Female	5	5	5
**WBC** (/μl)	4,100 ± 994	5,200 ± 2,394 (*P*=0.19639)	4,600 ± 2011 (*P*=0.48998)
**RBC** (x10^4/μl)	785 ± 87	898 ± 186 (*P*=0.09853)	934 ± 179 (*P*=0.02976)
**Hb** (g/dl)	11.7 ± 0.67	12.5 ± 1.65 (*P*=0.17294)	12.8 ± 1.99 (*P*=0.11501)
**HCT** (%)	33.0 ± 3.16	38.2 ± 7.38 (*P*=0.05532)	39.3 ± 7.15 (*P*=0.02017)
**MCV** (fl)	42.1 ± 1.19	42.6 ± 1.07 (*P*=0.28208)	42.1 ± 1.14 (*P*=0.89425)
**MCH** (pg)	15.0 ± 1.62	12.7 ± 4.62 (*P*=0.14805)	13.8 ± 1.22 (*P*=0.07590)
**PLT** (x10^4/μl)	156.7 ± 106	102.5 ± 60.45 (*P*=0.17814)	136.4 ± 85.92 (*P*=0.64430)

For analysis of blood cell counts, PB from the postorbital vein was analysed by a particle counter PCE-310 (ERMA Inc., Tokyo, Japan). Values are the mean ± SD for ten mice per genotype. **WBC**, white blood cell; **RBC**, red blood cell; **Hb**, haemoglobin; **HCT**, haematocrit; **MCV**, mean corpuscular volume; **MCH**, mean corpuscular hemoglobin; **PLT**, platelet (n=10 biologically independent samples) (*P*-value, unpaired two-sided Student's t-test) Source data are available in Source Data file.

Supplementary Method 1

Analysis for LPA

Chemicals

Oleoyl-*L*-α-Lysophosphatidic acid (LPA) (Merck KGaA, Darmstadt, Germany), HPLC-grade methanol and 2-propanol (Kanto Chemical Co., Inc., Tokyo, Japan), ammonium acetate and 28% ammonia solution (Fujifilm Wako pure chemical corporation, Osaka, Japan) were purchased. Ultrapure water was prepared by using the milli-Q system (Millipore, Billerica, MA, USA).

LC/MS conditions

LPAs were analysed using an LC/MS system consisted of NexeraX2 system (Shimadzu Corporation, Kyoto, Japan) and Triple Quad 5500 (Sciex, Framingham, MA, USA). A reversed-phase column (Mastro C18, 2.1x150 mm, 3 μm, Lot No. VR08-035, Shimadzu GLC Ltd., Tokyo, Japan) was used for chromatographic separation. For mobile phases A and B, 20 mM ammonium acetate buffer (pH8.5), and 2-propanol were used, respectively. The flow rate was 0.25 mL/min. The sample cooler and column oven temperatures were set at 5 °C and 40 °C, respectively. The gradient of mobile phase B concentration was programmed as 40%(0 min)-40%(1 min)-98%(9.5 min)-98%(13 min)-40%(13.1 min)-40%(15 min). Methanol was used as a sample solvent. Sample injection volume was 5 μL. Electrospray inonization (ESI) in the negative ion mode was used in this study. For MS condition, multiple reaction monitoring (MRM) were used in

order to detect LPA12:0 to LPA22:6. To determine an absolute calibration curve, LPA18:1 was diluted 10, 25, 50, 100, 250, 500 and 1000 ng/mL with methanol. MRM table was indicated as below:

**Negative**	**LPA**	
Compound	Q1 (*m/z*)	Q3 (*m/z*)
LPA 12:0	353.3	152.9
LPA 14:0	381.3	152.9
LPA 14:1	379.3	152.9
LPA 15:0	395.3	152.9
LPA 16:0	409.4	152.9
LPA 16:1	407.3	152.9
LPA 17:0	423.4	152.9
LPA 17:1	421.4	152.9
LPA 18:0	437.4	152.9
LPA 18:1	435.4	152.9
LPA 18:2	433.4	152.9
LPA 18:3	431.4	152.9
LPA 20:0	465.5	152.9
LPA 20:1	463.5	152.9
LPA 20:2	461.4	152.9

LPA 20:3	459.4	152.9
LPA 20:4	457.4	152.9
LPA 20:5	455.4	152.9
LPA 22:0	493.5	152.9
LPA 22:4	485.5	152.9
LPA 22:5	483.4	152.9
LPA 22:6	481.4	152.9

Sample preparation

Total BMMNCs ($1x10^7$) were isolated from *Gdpd3*$^{+/+}$ tet-CML-affected mice (5 females), *Gdpd3*$^{-/-}$ tet-CML-affected mice (1 male and 3 females) at 5 weeks post-Dox withdrawal as described above. Total BMMNCs ($1x10^7$) were also isolated from 6-8 wk-old WT C57BL/6 mice (2 males and 4 females). Cell pellets were frosen at -80°C immediately after centrifugation. Five hundred milliliter of methanol was added to the frozen cell pellet. For lipid extraction, the solutions was performed ultrasonic for 1 min at 4 °C, and centrifuged at approximately 11,000 × g for 5 min. The supernatant was subjected to chromatography.

Data analysis

An absolute calibration curve was determined by standard LPA18:1 samples. Concentration and ratio of each sample were semiquantitatively determined based on the absolute calibration curve. Analyst Ver. 1.7 (Sciex, Framingham, MA, USA) software was used for the data processing, and the MRM data analysis in this study. Limit of detection (LOD) was defined with a signal-to-noise ratio (S/N) of 3.

Chromatogram

Representative chromatogram data were indicated as below:

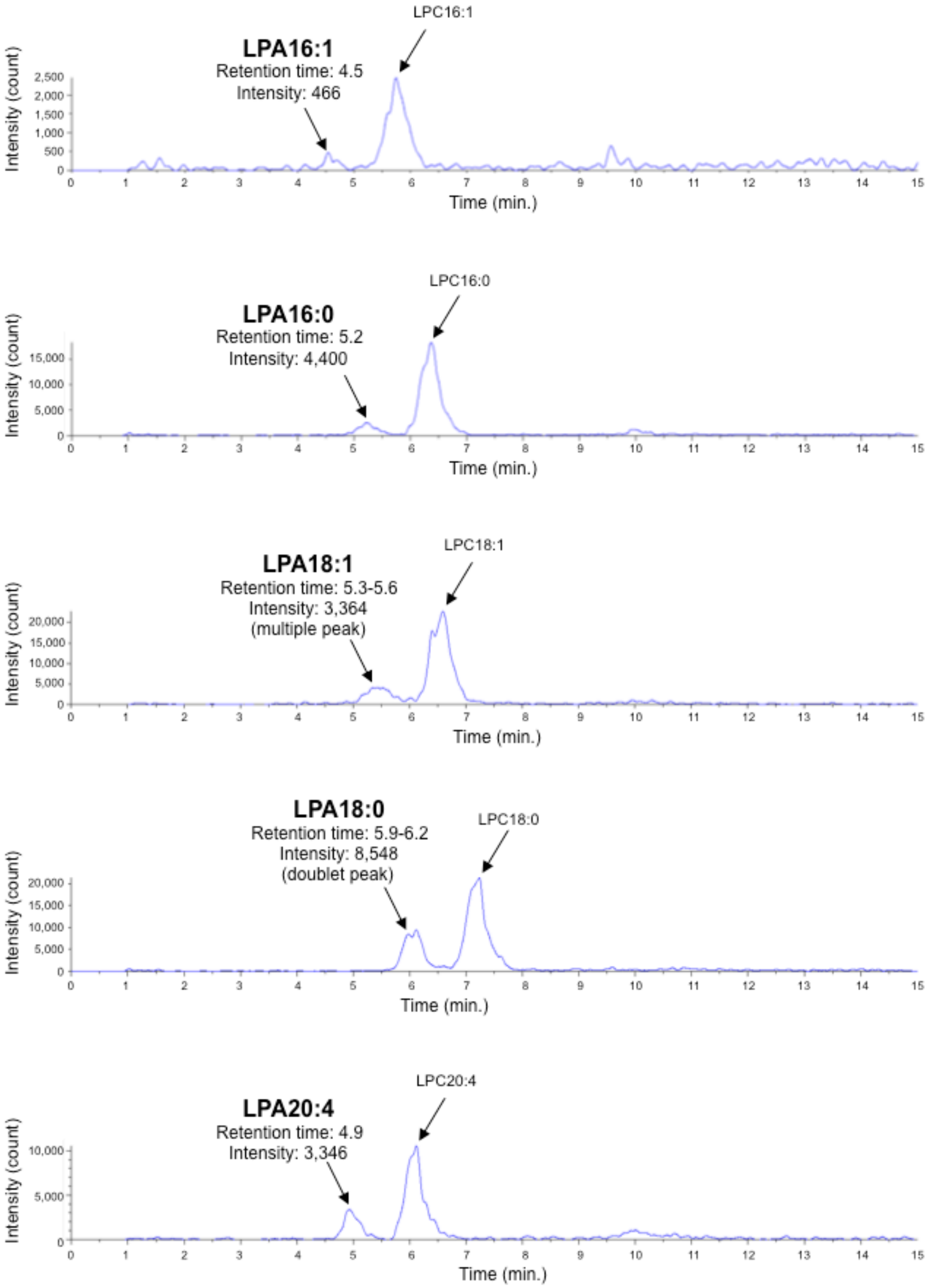

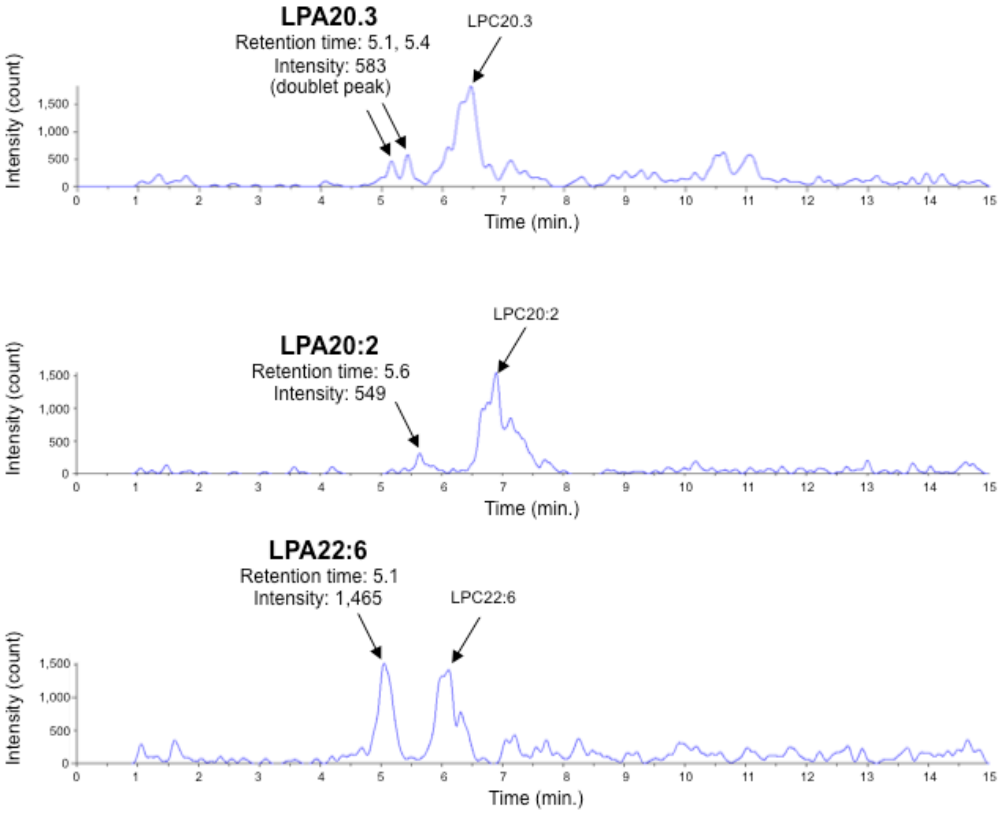
LPA20.3
Retention time: 5.1, 5.4
Intensity: 583
(doublet peak)
LPC20.3
Intensity (count)
Time (min.)
LPA20:2
Retention time: 5.6
Intensity: 549
LPC20:2
Intensity (count)
Time (min.)
LPA22:6
Retention time: 5.1
Intensity: 1,465
LPC22:6
Intensity (count)
Time (min.)

Supplementary Method 2

Analysis for lipid mediators

Chemicals

All lipid standards, including 196 target compounds (listed in **Source Data Fig3b.xls**) and 18 deuterium-labeled internal standards (see below) were purchased from Cayman Chemical (Ann Arbor, MI, USA).

Internal Standard (IS)	
11,12-EET-d_{11}	Cayman Chemical (Ann Arbor, MI, USA)
12-HETE-d_8	
14,15-DiHET-d_{11}	
15-HETE-d_8	
5-HETE-d_8	
6-keto-$PGF_{1\alpha}$-d_4	
AA-d_8	
LTB_4-d_4	
LTC_4-d_5	
LTD_4-d_5	
OEA-d_4	
PAF-d_4	
PGA_2-d_4	
PGD_2-d_4	
PGE_2-d_4	
$PGF_{2\alpha}$-d_4	
tetranor-PGEM-d_6	
TXB_2-d_4	

These chemicals were dissolved in methanol and stored at −80 °C. LC/MS-grade acetonitrile (Kanto Chemical Co., Inc., Tokyo, Japan), HPLC-grade formic acid (Fujifilm Wako pure chemical corporation, Osaka, Japan), HPLC-grade methanol (Kanto Chemical Co., Inc.), and ethanol (Fujifilm Wako pure chemical) were purchased.

Ultrapure water was prepared by using the milli-Q system (Millipore, Billerica, MA, USA).

LC/MS conditions

Lipid mediators were analysed using an LC/MS system consisted of Nexera UHPLC LC-30A pumps, an SIL-30AC auto-sampler, a CTO-20A column oven, a CBM-20A system controller, and a triple quadrupole mass spectrometer LCMS-8050 (Shimadzu, Kyoto, Japan) as described previously[1]. A reversed-phase column (Kinetex C8 100A, 2.1 × 150 mm, 2.6 μm, Phenomenex, Torrance, CA, USA) was used for chromatographic separation. For mobile phases A and B, 0.1% formic acid in water, and acetonitrile were used, respectively[1]. The flow rate was 0.4 mL/min. The sample cooler and column oven temperatures were set at 5 °C and 40 °C, respectively. The gradient of mobile phase B concentration was programmed as 10% (0min)-25% (5min)-35% (10min)-75% (20min)-95% (20.1min)-95% (25.0 min)-10% (25.1 min)-10% (28 min). Methanol was used as a sample solvent. Sample injection volume was 5 μL. MS condition was followed by LC/MS/MS Method Package Lipid Mediators (Version 3)[1] (Shimadzu Corporation) (https://www.shimadzu.com/an/lcms/lipid_mediators.html).

Sample preparation

Total BMMNCs (1x10^{7}) were isolated from *Gdpd3*$^{+/+}$ tet-CML-affected mice (5 females), *Gdpd3*$^{-/-}$ tet-CML-affected mice (1 male and 3 females) at 5 weeks post-Dox withdrawal as described above. Total BMMNCs (1x10^{7}) were also isolated from 6-8 wk-old WT C57BL/6 mice (2 males and 4 females). Cell pellets were frosen at -80°C immediately after centrifugation. Two hundred microlitter of 0.1% formic acid in water

was added to the frozen cell pellet and homogenized the sample. One milliliter of 0.1%formic acid in methanol was added to the sample, followed by the addition of 10 µL of IS mixture. The suspension was centrifuged at 10,000 × g for 5 min, 4℃. The supernatant was diluted with 4mL of 0.1% formic acid in water and loaded onto preconditioned solid-phase extraction column cartridge (Oasis HLB VAC RC, 30 mg, Waters). The cartridge was washed with 1mL each of 0.1% formic acid, and 0.1% formic acid in 15% ethanol. Then, the lipids were eluted with 200µL of 0.1% formic acid in methanol. The eluent was evaporated by vacuum evaporator and reconstituted in 20 µL of 0.1% formic acid in methanol, and 5µL was injected for analysis. Blank was prepared same procedure without sample.

Lower limit of quantification (LLOQ) and linear dynamic range

An internal standard method was used for quantification as described previously[1] with modification. A mixture of 18 internal standards (IS mixture) containing 1.0 µg/mL each of the 11,12-EET-d_{11}, 14,15-DiHET-d_{11}, tetranor-PGEM-d_6, TXB_2-d_4, PGE_2-d_4, PGD_2-d_4, LTC_4-d_5, LTB_4-d_4, 15-HETE-d_8, 5-HETE-d_8, oleoylethanolamide (OEA)-d_4, EPA-d_5, and DHA-d_5, 2.0 µg/mL each of the, 12-HETE-d_8, 15-HETE-d_8, and PAF-d_4, 20 µg/mL 6-keto PGF1α-d_4, and 50 µg/mL AA-d_8 was used in this study. LLOQ was defined as described previously[1]. Briefly, the accuracy and CV at LLOQ were in the range of 80–120% and within 20%, respectively, and the accuracy and CV at all the calibration points above LLOQ were in the range of 85–115% and within 15%, respectively. Limit of detection (LOD) was defined with a signal-to-noise ratio (S/N) of

3 as described previously[1]. The groups for lipid detection and IS used in this study were listed as below:

Category	Compound Name	CAS No.	Transition (m/z)[1]	IS
AA	11,12-EET	123931-40-8	319.2>167.1	11,12-EET-d_{11}
EDA	11-HEDE	5598-37-8	323.3>199.1*	11,12-EET-d_{11}
AA	14,15-EET	197508-62-6	319.2>257.2	11,12-EET-d_{11}
EDA	15-HEDE	77159-57-0	323.2>223.2	11,12-EET-d_{11}
DHA	16,17-EpDPA	155073-46-4		11,12-EET-d_{11}
AA	5,6-EET	87173-80-6	319.2>191.1	11,12-EET-d_{11}
DGLA	5-HETrE	213382-49-1		11,12-EET-d_{11}
AA	**5-KETE**	106154-18-1	317.2>203.2	11,12-EET-d_{11}
AA	8,9-EET	N.A.	319.2>155.1	11,12-EET-d_{11}

(*; unpublished information)

Category	Compound Name	CAS No.	Transition (m/z) [1]	IS
DHA	10-HDHA	90780-50-0	343.2>153.1	12-HETE-d_8
AA	11-HETE	54886-50-9	319.2>167.1	12-HETE-d_8
AA	12-HETE	54397-83-0	319.2>179.1	12-HETE-d_8
DHA	13-HDHA	90780-53-3	343.2>193.1	12-HETE-d_8
EPA	14,15-EpETE	131339-24-7		12-HETE-d_8
DHA	14-HDHA	87042-40-8	343.2>205.2	12-HETE-d_8
AA	15-HpETE	70981-96-3	335.2>113.1	12-HETE-d_8
AA	15-KETE	81416-72-0		12-HETE-d_8
DHA	16-HDHA	90780-51-1	343.2>233.2	12-HETE-d_8
DHA	**17-HDHA**	90780-52-2	343.2>245.2	12-HETE-d_8
AA	8-HETE	98462-03-4	319.2>155.1	12-HETE-d_8
LA	9-KODE	54232-59-6	293.2>185.1	12-HETE-d_8

Category	Compound Name	CAS No.	Transition (m/z) [1]	IS
EA	11,12-EET-EA	N.A.	364.3>62.1*	15-HETE-d_8
EPA	11-HEPE	99217-78-4		15-HETE-d_8
EPA	12-HEPE	116180-17-7	317.2>179.1	15-HETE-d_8
EPA	12-HpEPE	103239-14-1	333.2>271.2	15-HETE-d_8
LA	13-HODE	29623-28-7	295.2>195.1	15-HETE-d_8
LA	13-HpODE	23017-93-8		15-HETE-d_8
ALA	13-HpOTrE	67597-26-6		15-HETE-d_8
LA	13-KODE	54739-30-9	293.2>113.1	15-HETE-d_8
EPA	**15-HEPE**	86282-92-0	319.2>219.2	15-HETE-d_8
AA	15-HETE	54845-95-3	319.2>219.2	15-HETE-d_8
EPA	15-HpEPE	125992-60-1	333.2>111.1	15-HETE-d_8
AA	16-HETE	128914-46-5	319.2>233.2	15-HETE-d_8
EPA	17,18-EpETE	N.A.		15-HETE-d_8
AA	17-HETE	128914-47-6	319.2>247.2	15-HETE-d_8
AA	18-HETE	133268-58-3	319.2>261.2	15-HETE-d_8
DHA	20-HDHA	90906-41-5	343.2>241.2	15-HETE-d_8
AA	20-HETE	79551-86-3	319.2>275.2	15-HETE-d_8
EA	5,6-EET-EA	N.A.	364.3>62.1*	15-HETE-d_8
EPA	5-HEPE	92008-51-0	317.2>115.1	15-HETE-d_8
EPA	5-HpEPE	143292-98-2	333.2>173.1	15-HETE-d_8
EA	8,9-EET-EA	N.A.	364.3>62.1*	15-HETE-d_8
EPA	8-HEPE	99217-77-3		15-HETE-d_8
EPA	9-HEPE	286390-03-2		15-HETE-d_8
LA	9-HODE	73543-67-6	295.2.171.1	15-HETE-d_8
LA	9-HpODE	5502-91-0	311.2>185.2*	15-HETE-d_8
	Lyso-PAF	52691-62-0	482.3>104.2*	15-HETE-d_8

**Category**	**Compound Name**	**CAS No.**	**Transition (m/z) [1]**	**IS**
AA	11,12-DHET	N.A.	337.2>167.1	14,15-DiHET-d_{11}
LA	12,13-DiHOME	263399-35-5	313.2>183.1*	14,15-DiHET-d_{11}
AA	12-HHT	54397-84-1	279.2>179.1	14,15-DiHET-d_{11}
AA	12-keto-LTB_4	136696-10-1	333.2>179.1	14,15-DiHET-d_{11}
ALA	13-HOTrE	87984-82-5	293.2>195.1	14,15-DiHET-d_{11}
AA	14,15-DHET	N.A.	337.2>207.2	14,15-DiHET-d_{11}
EPA	14,15-DiHETE	N.A.	335.2>207.2	14,15-DiHET-d_{11}
EA	14,15-EET-EA	N.A.	364.3>62.1	14,15-DiHET-d_{11}
AA	15-deoxy-$\Delta^{12,14}$-PGJ_2	87893-55-8	315.2>271.2	14,15-DiHET-d_{11}
EPA	18-HEPE	141110-17-0	317-2>215.2	14,15-DiHET-d_{11}
DHA	19,20-DiHDPA	N.A.		14,15-DiHET-d_{11}
AA	19-HETE	115461-40-0		14,15-DiHET-d_{11}
AA	20-Carboxy-AA	79551-84-1	333.2>297.2	14,15-DiHET-d_{11}
AA	5,6-DHET	213382-49-1	337.2>145.1	14,15-DiHET-d_{11}
EPA	5,6-DiHETE	845673-97-4	335,2>145.1	14,15-DiHET-d_{11}
DHA	7,17-hydroxy-DPA	887752-13-8		14,15-DiHET-d_{11}
AA	8,9-DHET	N.A.	337.2>127.1	14,15-DiHET-d_{11}
LA	9,10-DiHOME	263399-34-4	313.2>201.2*	14,15-DiHET-d_{11}
ALA	9-HOTrE	89886-42-0	293.2>171.1	14,15-DiHET-d_{11}
AA	LTB_3	88099-35-8		14,15-DiHET-d_{11}
AA	*N*-Acetyl-LTE_4	80115-95-3	480.2>333.2	14,15-DiHET-d_{11}
AA	tetranor-12-HETE	121842-79-3	265.2>109.1*	14,15-DiHET-d_{11}

Category	Compound Name	CAS No.	Transition (m/z) [1]	IS
DHA	11-HDHA	87018-59-5	343.2>149.1	5-HETE-d_8
LA	12,13-EpOME	N.A.		5-HETE-d_8
AA	12-HpETE	71774-10-2	335-2>153.1	5-HETE-d_8
AA	12-KETE	108437-64-5	317.2>153.1	5-HETE-d_8
DGLA	15-HETrE	92693-02-2	321.2>221.2	5-HETE-d_8
DHA	17-HpDHA	123673-33-6		5-HETE-d_8
DHA	19,20-EpDPA	N.A.		5-HETE-d_8
DHA	4-HDHA	90906-40-4	343.2>101.1	5-HETE-d_8
AA	5,6-DHET-lactone	213126-92-2	321.2>177.1	5-HETE-d_8
AA	5-HETE	70608-72-9	319.2>115.1	5-HETE-d_8
AA	5-HpETE	71774-08-8	335.2>129.1	5-HETE-d_8
DHA	7-HDHA	90780-55-5	343.2>141.1	5-HETE-d_8
DHA	8-HDHA	90780-54-4	343.2>109.1	5-HETE-d_8
DGLA	8-HETrE	889573-69-7	321.2>157.2*	5-HETE-d_8
LA	9,10-EpOME	N.A.		5-HETE-d_8
AA	9-HETE	79495-85-5		5-HETE-d_8

Category	Compound Name	CAS No.	Transition (m/z) [1]	IS
AA	11-dehydro-2,3-dinor-TXB_2	79250-60-5		6-keto-$PGF_{1\alpha}$-d_4
AA	13,14-dihydro-15-keto-tetranor-PGD_2	1204116-69-7	297.2>109.1	6-keto-$PGF_{1\alpha}$-d_4
AA	13,14-dihydro-15-keto-tetranor-PGE_2	20675-85-8	297.2>109.1	6-keto-$PGF_{1\alpha}$-d_4
AA	13,14-dihydro-15-keto-tetranor-$PGF_{1\alpha}$	24379-94-0	299.2>113.1	6-keto-$PGF_{1\alpha}$-d_4
AA	13,14-dihydro-15-keto-tetranor-$PGF_{1\beta}$	23015-45-4	299.2>113.1	6-keto-$PGF_{1\alpha}$-d_4
AA	18-carboxy-dinor-LTB_4	102674-12-4	337.2>59.1	6-keto-$PGF_{1\alpha}$-d_4
AA	2,3-dinor-11β-$PGF_{2\alpha}$	240405-20-3		6-keto-$PGF_{1\alpha}$-d_4
AA	2,3-dinor-8-iso-$PGF_{2\alpha}$	221664-05-7	325.2>237.2	6-keto-$PGF_{1\alpha}$-d_4
DGLA	2,3-dinor-PGE_1	7046-40-4		6-keto-$PGF_{1\alpha}$-d_4
AA	20-carboxy-LTB_4	80434-82-8	365.2>169.1	6-keto-$PGF_{1\alpha}$-d_4
AA	20-hydroxy-LTB_4	79516-82-8	351.2>195.1	6-keto-$PGF_{1\alpha}$-d_4
AA	20-hydroxy-PGE_2	57930-95-7	367.2>287.2	6-keto-$PGF_{1\alpha}$-d_4
AA	20-hydroxy-$PGF_{2\alpha}$ or 19-hydroxy-$PGF_{2\alpha}$	57930-92-4 or 64625-53-2	369.2>325.2	6-keto-$PGF_{1\alpha}$-d_4
AA	6,15-diketo-13,14-dihydro-$PGF_{1\alpha}$	63983-53-9	369.2>113.1	6-keto-$PGF_{1\alpha}$-d_4
DGLA	6-keto-PGE_1	67786-53-2		6-keto-$PGF_{1\alpha}$-d_4
AA	6-keto-$PGF_{1\alpha}$	58962-34-8	369.3>245.2	6-keto-$PGF_{1\alpha}$-d_4
EPA	8-iso-$PGF_{3\alpha}$	7045-31-0	351.2>307.2	6-keto-$PGF_{1\alpha}$-d_4
EPA	Δ^{17}-6-keto-$PGF_{1\alpha}$	68324-95-8		6-keto-$PGF_{1\alpha}$-d_4
EA	PGD_2-EA	398138-28-8	440.3>271.2	6-keto-$PGF_{1\alpha}$-d_4
EA	PGE_1-EA	210976-81-1	442.3>360.2	6-keto-$PGF_{1\alpha}$-d_4
EA	PGE_2-EA	194935-38-1	440.3>271.2	6-keto-$PGF_{1\alpha}$-d_4
EA	$PGF_{2\alpha}$-EA	353787-70-9	442.3>334.2	6-keto-$PGF_{1\alpha}$-d_4
EPA	Resolvin E_1	552830-51-0		6-keto-$PGF_{1\alpha}$-d_4

Category	Compound Name	CAS No.	Transition (m/z) [1]	IS
AA	AA	506-32-1	303.2>303.2	AA-d_8
DHA	DHA	6217-54-5	327.2>229.2	AA-d_8
EPA	EPA	10417-94-4	301.2>257.2	AA-d_8

Category	Compound Name	CAS No.	Transition (m/z) [1]	IS
DHA	10,17-DiHDHA	871826-47-0	359.2>153.1*	LTB_4-d_4
AA	13,14-dihydro-15-keto PGJ_2	N.A.	333.2>175.1	LTB_4-d_4
AA	13,14-dihydro-15-keto-PGA_2	74872-89-2		LTB_4-d_4
EPA	17,18-DiHETE	N.A.	335.2>247.2	LTB_4-d_4
AA	5,15-DiHETE	82200-87-1	335.2>173.1	LTB_4-d_4
AA	6-*trans*-LTB_4	71652-82-9	335.2>195.1	LTB_4-d_4
AA	8,12-iso-$iPF_{2\alpha}$-VI-1,5-lactone	N.A.	337.3>265.2	LTB_4-d_4
AA	8,15-DiHETE	80234-65-7	335.2>127.1	LTB_4-d_4
AA	LTB_4	71160-24-2	335.2>195.1	LTB_4-d_4
DHA	Maresin 1	1784701-61-6		LTB_4-d_4
DHA	Resolvin D_5	578008-43-2	359.2>199.1*	LTB_4-d_4

Category	Compound Name	CAS No.	Transition (m/z) [1]	IS
AA	11-*trans*-LTC_4	74841-69-3	626.3>189.1	LTC4-d_5
AA	11-*trans*-LTE_4	75715-88-7	440.2>189.1	LTC4-d_5
AA	14,15-LTC_4	75290-60-7	626.3>308.2	LTC4-d_5
AA	14,15-LTE_4	1000852-57-2	440.3>301.2	LTC4-d_5
AA	LTC_4	72025-60-6	626.3>308.2	LTC4-d_5
AA	LTE_4	75715-89-8	440.2>189.1	LTC4-d_5
AA	LTF_4	83851-42-7	569.3>251.2	LTC4-d_5

Category	Compound Name	CAS No.	Transition (m/z) [1]	IS
DGLA	13,14-dihydro-15-keto-PGD_1	1392219-79-2		PGA_2-d_4
DGLA	8-iso-PGA_1	211186-29-7	335.2>235.2	PGA_2-d_4
AA	8-iso-PGA_2	474391-66-7	333.2>271.2	PGA_2-d_4
EPA	LTB_5	80445-66-5		PGA_2-d_4
DGLA	PGA_1	14152-28-4		PGA_2-d_4
AA	PGA_2	13345-50-1	333.2>189.1	PGA_2-d_4
AA	PGB_2	13367-85-6	333.2>175.1	PGA_2-d_4
AA	PGJ_2	60203-57-8	333.2>271.2	PGA_2-d_4
DHA	Resolvin D4	1025684-60-9		PGA_2-d_4

Category	Compound Name	CAS No.	Transition (m/z) [1]	IS
AA	11-β-13,14-dihydro-15-keto-$PGF_{2\alpha}$	107615-77-0	353.2>183.1	PGD_2-d_4
AA	13,14-dihydro-15-keto-PGD_2	59894-07-4	351.2>207.2	PGD_2-d_4
AA	13,14-dihydro-15-keto-PGE_2	363-23-5	351.2>175.1	PGD_2-d_4
AA	13,14-dihydro-15-keto-$PGF_{2\alpha}$	27376-76-7	353.2>113.1	PGD_2-d_4
DGLA	13,14-dihydro-PGE_1	19313-28-1		PGD_2-d_4
AA	13,14-dihydro-$PGF_{1\alpha}$	20592-20-5		PGD_2-d_4
AA	15-keto-PGE_2	26441-05-4	349.2>113.1	PGD_2-d_4
AA	15-keto-$PGF_{1\alpha}$	21562-58-3		PGD_2-d_4
ADA	1-α,1-β-dihomo-$PGF_{2\alpha}$	57944-39-5		PGD_2-d_4
AA	5*S*,14*R*-LXB_4	98049-69-5	355.2>275.2*	PGD_2-d_4
AA	5*S*,6*R*-LXA_4	89663-86-5	351.2>115.1	PGD_2-d_4
AA	5*S*,6*S*-LXA_4	94292-80-5		PGD_2-d_4
EA	LTB_4-EA	877459-63-7	362.3>189.1	PGD_2-d_4
DGLA	PGD_1	17968-82-0	353.2>235.1	PGD_2-d_4
AA	**PGD_2**	41598-07-6	351.2>271.2	PGD_2-d_4
DGLA	PGE_1	745-65-3	353.2>235.2	PGD_2-d_4
AA	PGK_2	275816-51-8		PGD_2-d_4
DHA	Resolvin D_1	872993-05-0	375.2>141.1	PGD_2-d_4
DHA	Resolvin D_2	810668-37-2		PGD_2-d_4

**Category**	**Compound Name**	**CAS No.**	**Transition (m/z)** [1]	**IS**
AA	11-β-PGE_2	38310-90-6		PGE_2-d_4
AA	11-dehydro-TXB_2	67910-12-7	367.2>305.2	PGE_2-d_4
AA	15-keto-$PGF_{2\alpha}$	35850-13-6	351.2>219.2	PGE_2-d_4
DGLA	8-iso-PGE_1	21003-46-3	353.2>235.2	PGE_2-d_4
AA	8-iso-PGE_2	27415-25-4	351.2>271.2	PGE_2-d_4
EPA	LXA_5	110657-98-2	349.2>115.1	PGE_2-d_4
AA	**PGE_2**	363-24-6	351.2>271.2	PGE_2-d_4
DHA	Resolvin D_3	916888-47-6		PGE_2-d_4

**Category**	**Compound Name**	**CAS No.**	**Transition (m/z)** [1]	**IS**
AA	tetranor-PGAM	52510-53-9	309.1>163.1*	tetranor-PGEM-d_6
AA	tetranor-PGDM	70803-91-7	327.2>309.2	tetranor-PGEM-d_6
AA	tetranor-PGEM	24769-56-0	327.2>309.2	tetranor-PGEM-d_6
AA	tetranor-PGFM	23109-94-6	329.2>311.2	tetranor-PGEM-d_6
AA	tetranor-PGJM	1352751-83-7		tetranor-PGEM-d_6

**Category**	**Compound Name**	**CAS No.**	**Transition (m/z)** [1]	**IS**
EDA	15-KEDE	105835-44-7	323.2>207.2	OEA-d_4
EA	AEA	94421-68-8	348.3>62.1	OEA-d_4
EA	OEA	111-58-0	326.3>62.1	OEA-d_4

**Category**	**Compound Name**	**CAS No.**	**Transition (m/z)** [1]	**IS**
	**Azelaoyl-PAF**	354583-69-0	650.4>201.2*	PAF-d_4
	**PAF**	74389-68-7	568.4>59.1*	PAF-d_4

Category	Compound Name	CAS No.	Transition (m/z) [1]	IS
AA	11-β-$PGF_{2α}$	38432-87-0	353.2>193.1	$PGF_{2α}$-d_4
EPA	11-dehydro-TXB_3	129228-55-3		$PGF_{2α}$-d_4
AA	5-$iPF_{2α}$-VI	180469-63-0	353.2>115.1	$PGF_{2α}$-d_4
AA	8-iso-13,14-dihydro-15-keto-$PGF_{2α}$	191919-02-5	353.2>183.1	$PGF_{2α}$-d_4
AA	8-iso-15*R*-$PGF_{2α}$	214748-65-9	353.2>193.1	$PGF_{2α}$-d_4
AA	8-iso-15-keto-$PGF_{2α}$	191919-01-4	351.2>219.2	$PGF_{2α}$-d_4
DGLA	8-iso-$PGF_{1α}$	26771-96-0	355.2>293.2	$PGF_{2α}$-d_4
AA	**8-iso-$PGF_{2α}$**	27415-26-5	353.2>193.1	$PGF_{2α}$-d_4
AA	$iPF_{2α}$-IV	331962-00-6	353.2>127.1	$PGF_{2α}$-d_4
EPA	PGD_3	71902-47-1	349.2>269.2	$PGF_{2α}$-d_4
EPA	PGE_3	802-31-3	349.2>269.2	$PGF_{2α}$-d_4
DGLA	$PGF_{1α}$	745-62-0	355.2>211.1*	$PGF_{2α}$-d_4
AA	$PGF_{2α}$	551-11-1	353.2>193.1	$PGF_{2α}$-d_4
EPA	$PGF_{3α}$	745-64-2	351.2>193.1	$PGF_{2α}$-d_4

Category	Compound Name	CAS No.	Transition (m/z) [1]	IS
AA	11-*trans*-LTD_4	79768-40-4		LTD_4-d_5
AA	LTD_4	73836-78-9	497.3>189.1	LTD_4-d_5

Category	Compound Name	CAS No.	Transition (m/z) [1]	IS
DGLA	2,3-dinor-TXB_1	196493-76-2		TXB_2-d_4
AA	2,3-dinor-TXB_2	63250-09-9		TXB_2-d_4
DGLA	TXB_1	64626-32-0	371.2>171.2*	TXB_2-d_4
AA	TXB_2	54397-85-2	369.2>195.1	TXB_2-d_4
EPA	TXB_3	71953-80-5	367.2>169.1	TXB_2-d_4

Data analysis

LabSolutions Ver. 5.56 (Shimadzu) software was used for the data processing, and then, Traverse MS (Reifycs Inc., Tokyo, Japan) software was used for the MRM data analysis in this study (https://www.shimadzu.com/an/lcms/traverse_ms.html).

Chromatogram

Representative chromatogram data were indicated in Supplementary Method 3. Blue and Orange line indicate product ion. Either line was selected for monitor ion. Closed rectangle (▼) indicates sample peaks detected. Peak area values were indicated in the right of each chromatogram.

Reference

1. Yamada, M., *et al.* A comprehensive quantification method for eicosanoids and related compounds by using liquid chromatography/mass spectrometry with high speed continuous ionization polarity switching. *J. Chromatogr. B Analyt. Technol. Biomed. Life Sci.* **995-996**, 74-84 (2015).

002 tetranor-PGEM-d_6 (IS)

Retention time: 3.4

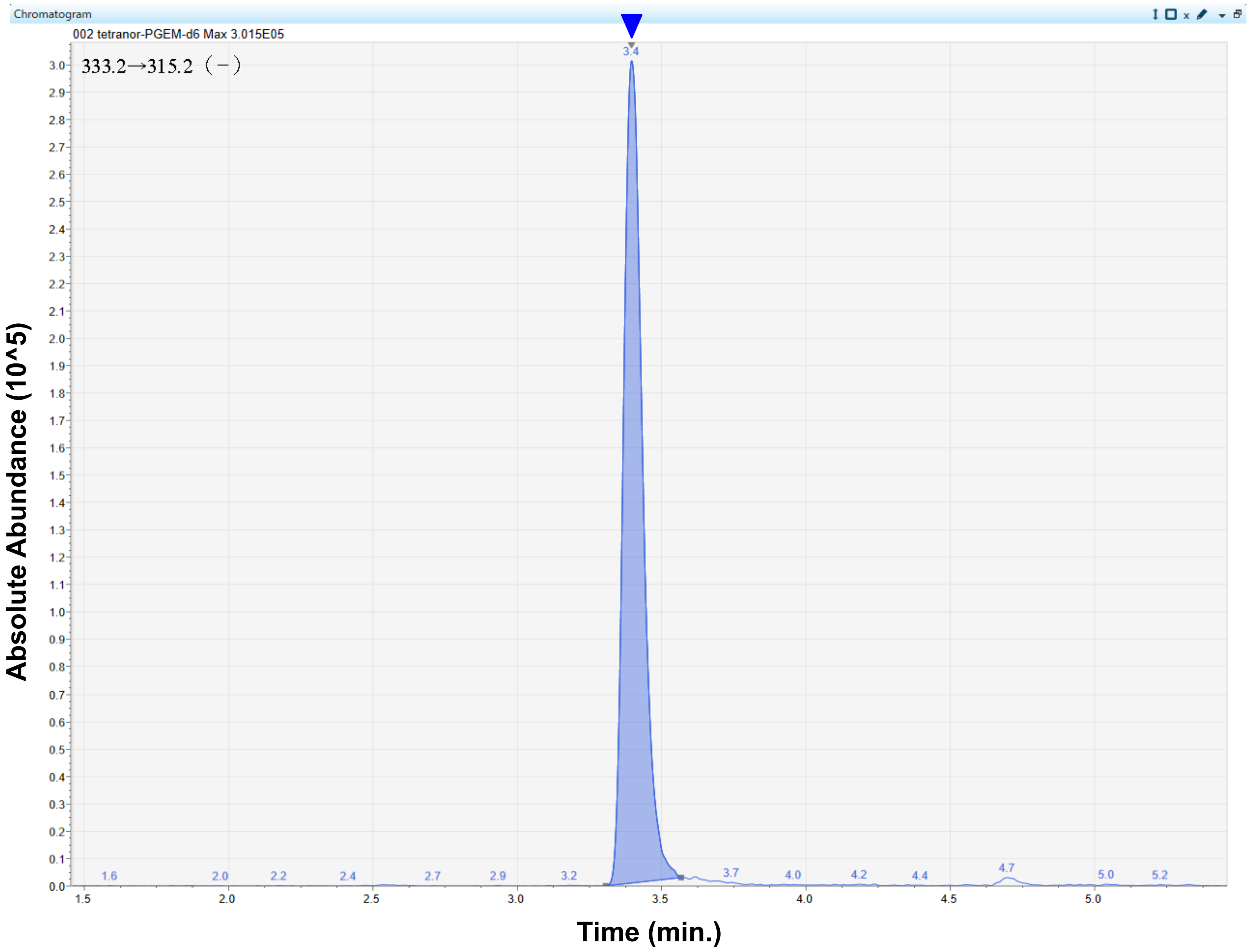

006 tetranor-PGAM

Retention time: 4.8

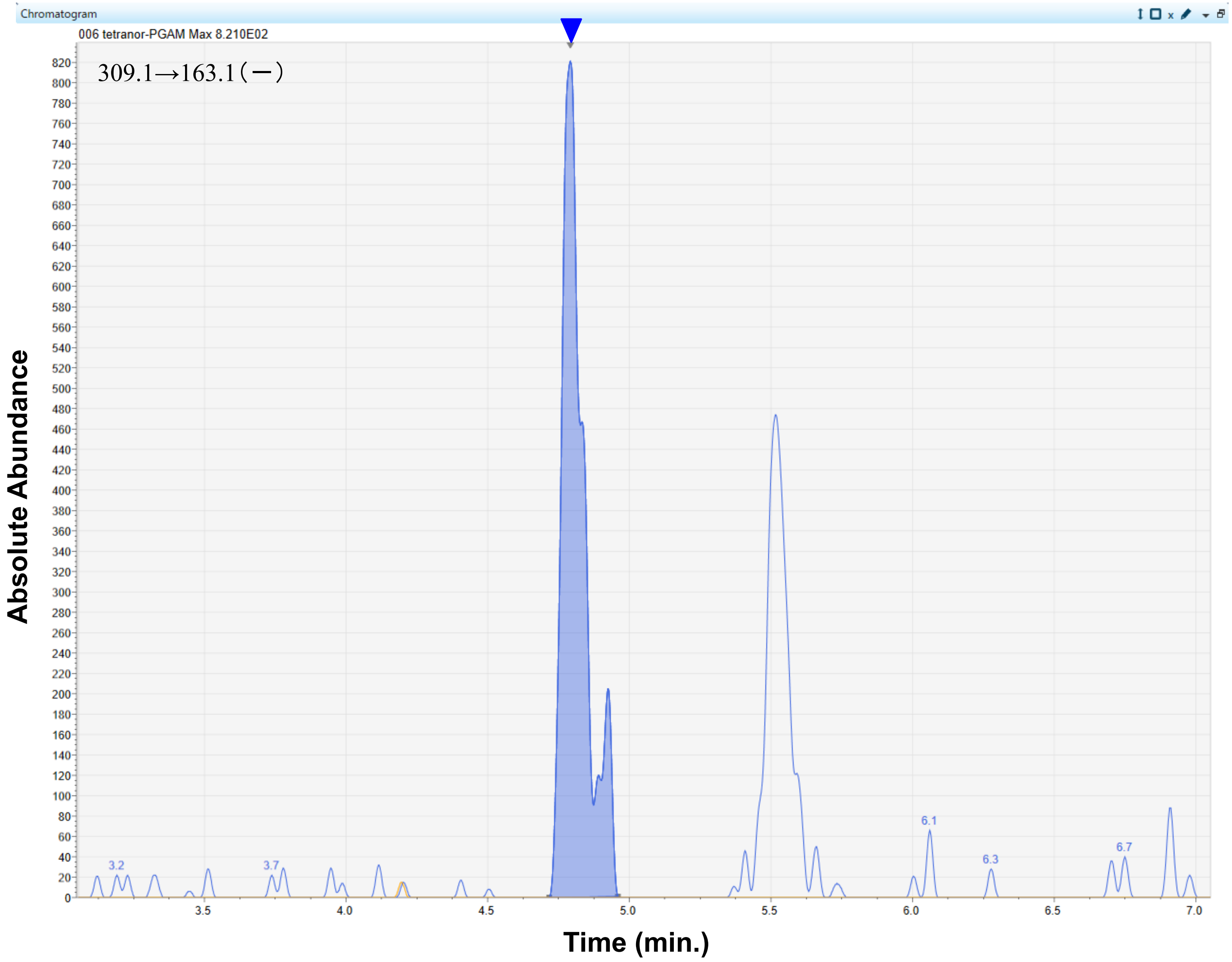

017 6-keto-PGF$_{1\alpha}$-*d*$_4$ (IS)

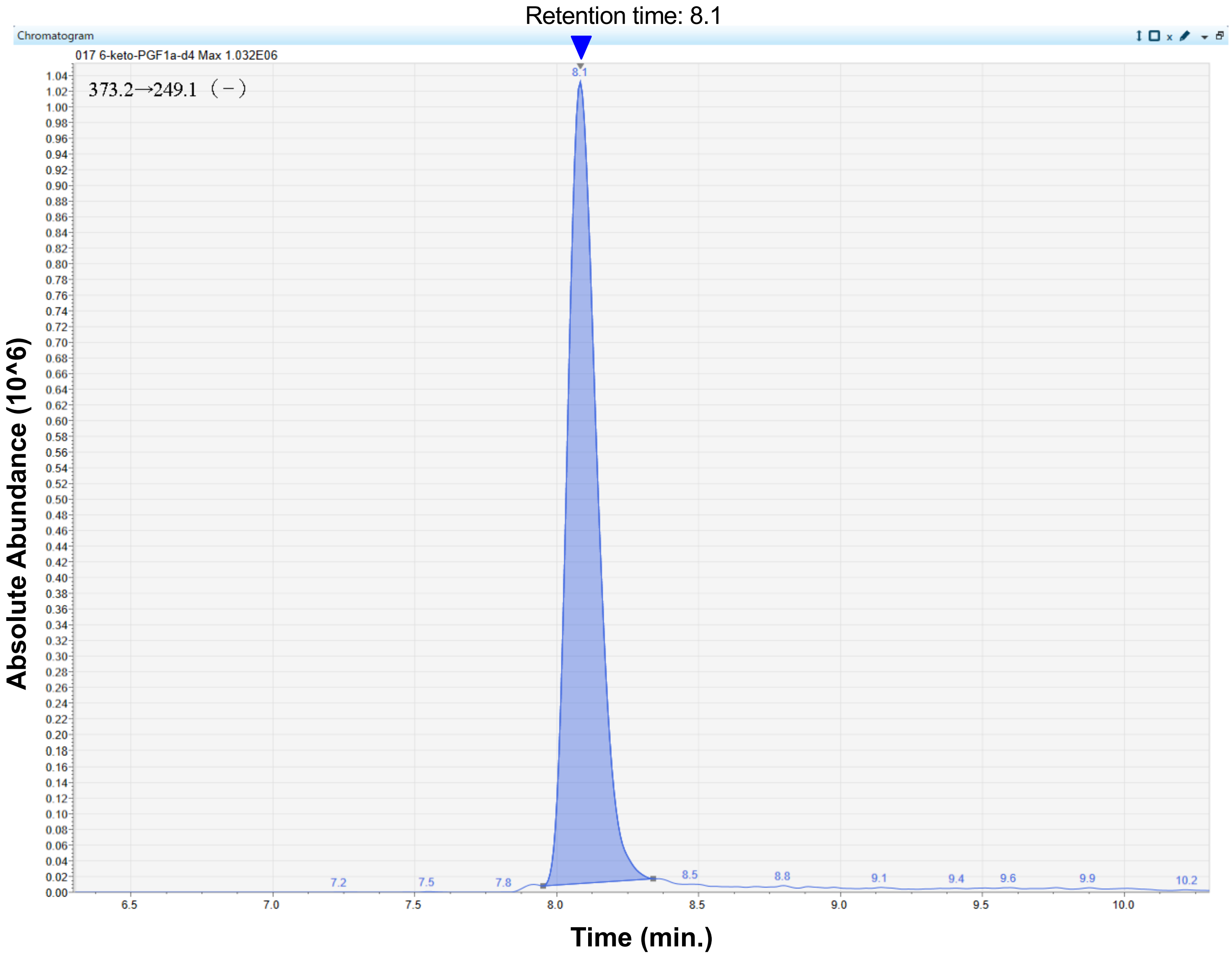

018 6-keto-$PGF_{1\alpha}$

Retention time: 8.1

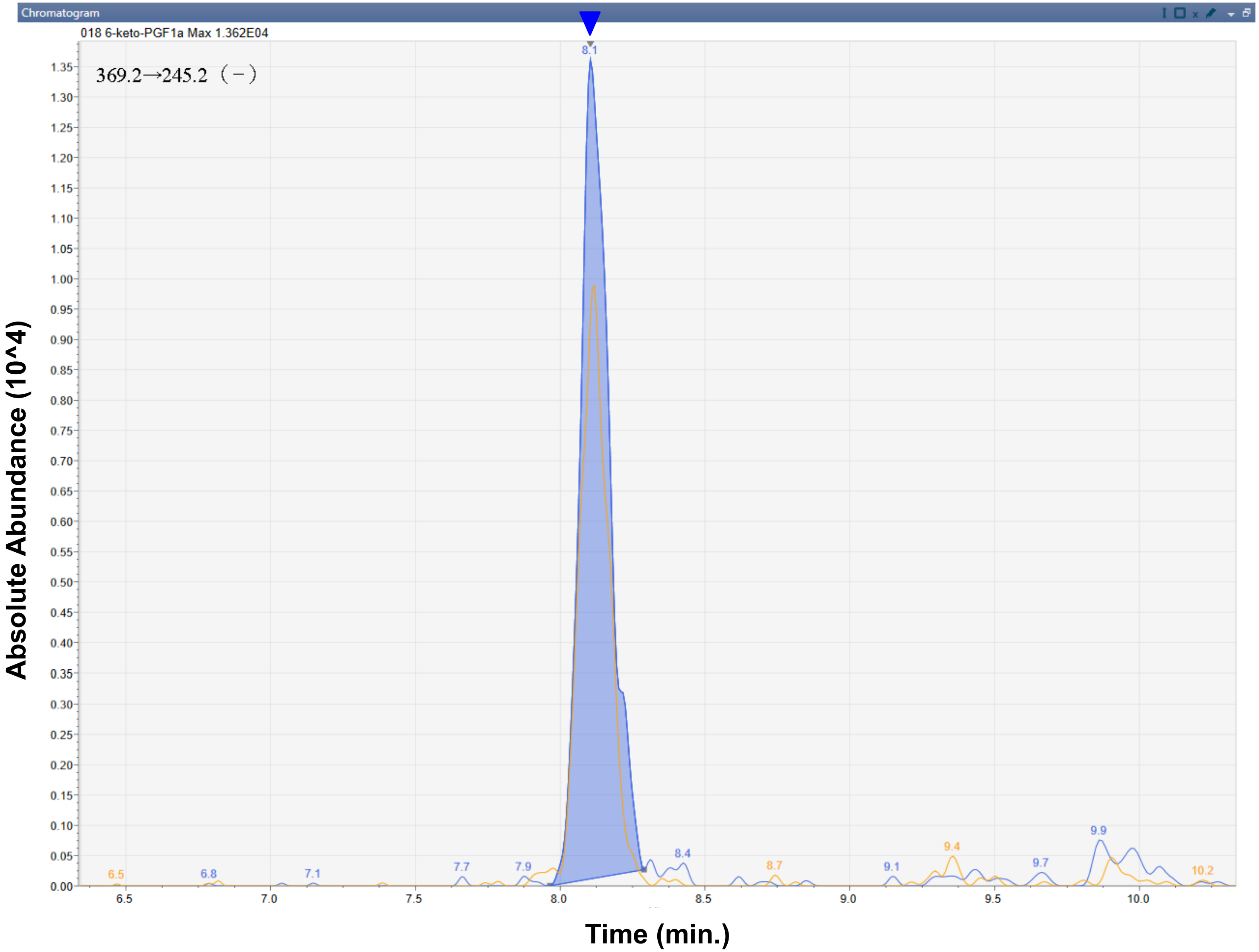

038 8-*iso*-PGF$_{2\alpha}$

Retention time: 9.8

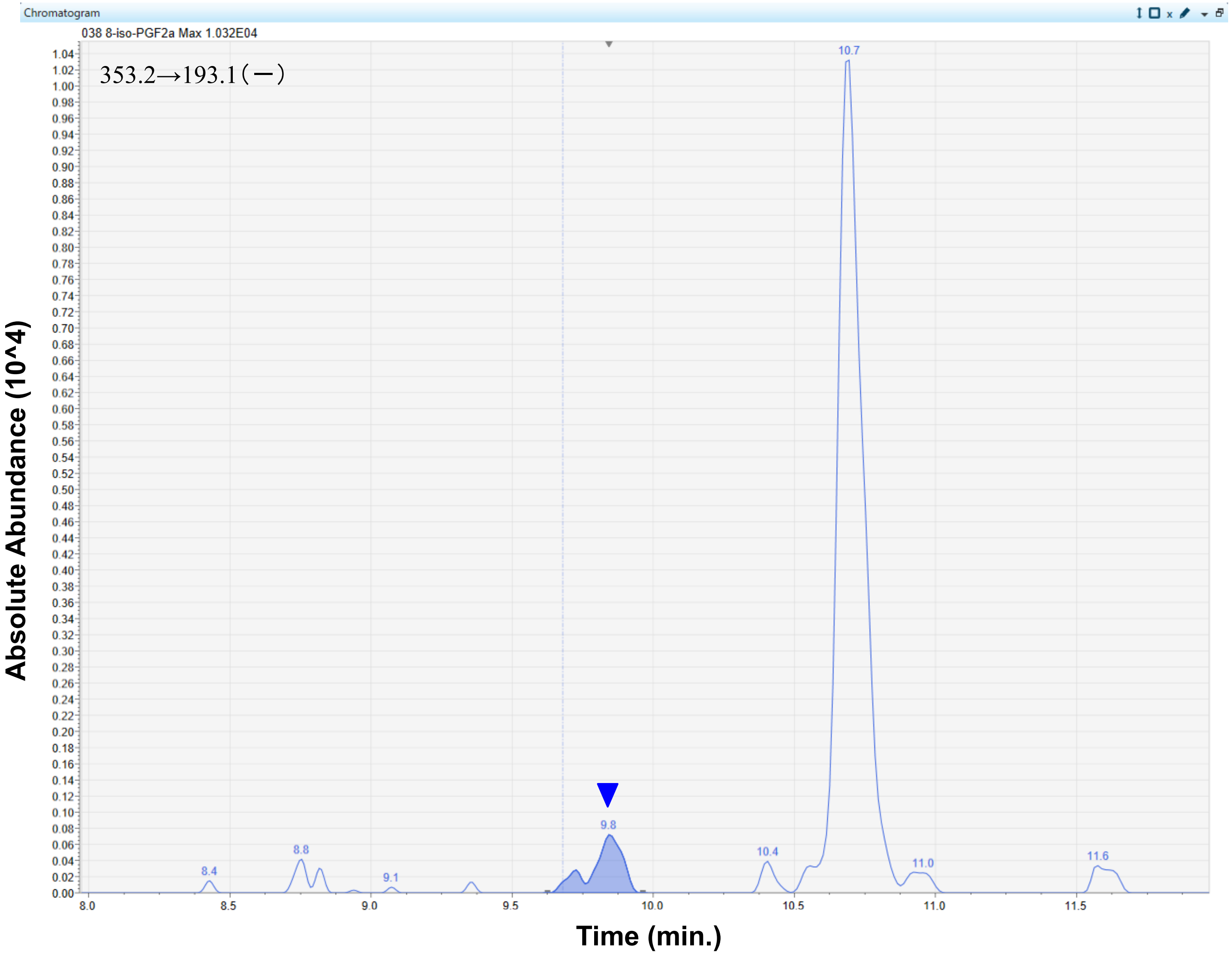

039 TXB_2-d_4 (IS)

Retention time: 9.9

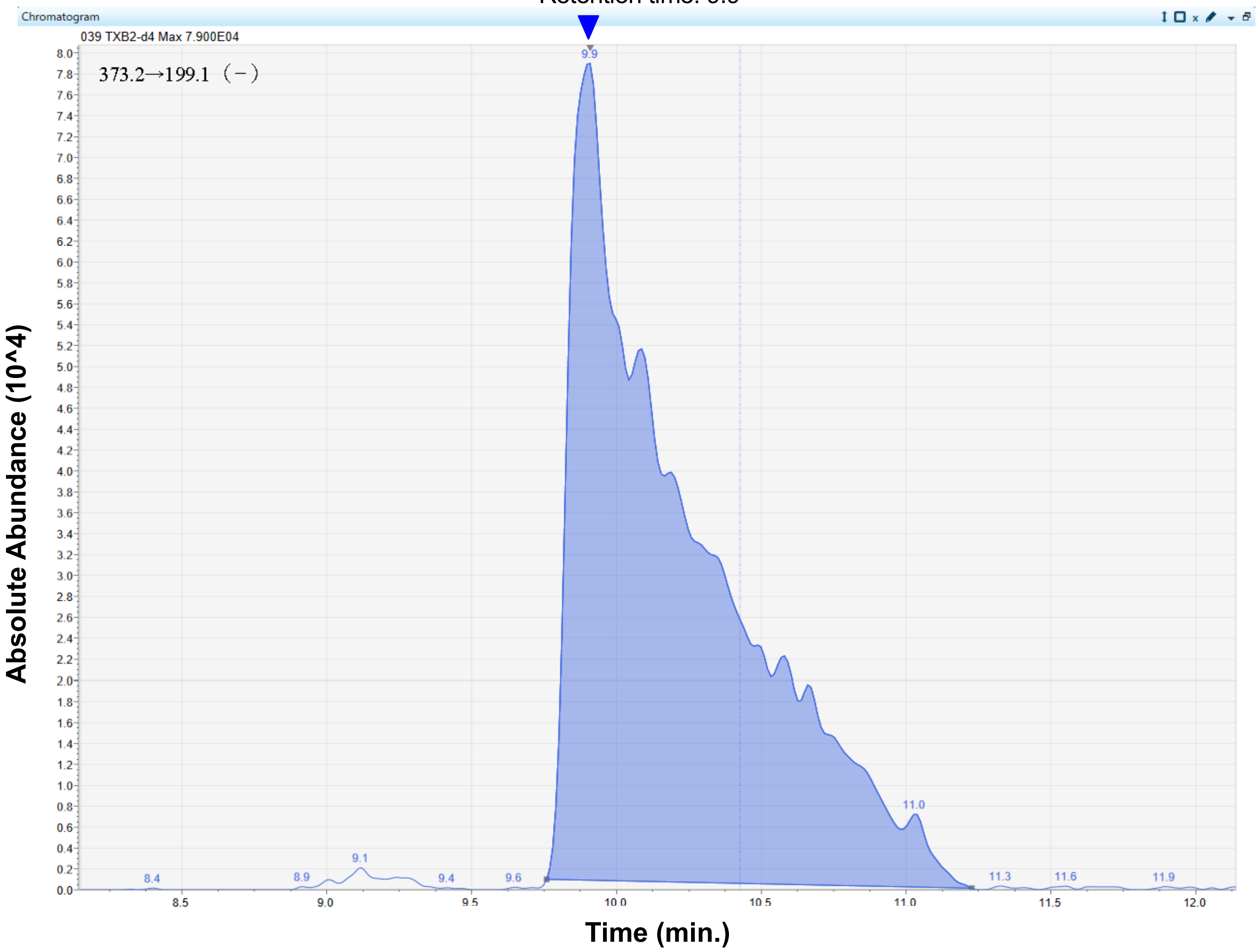

041 TXB_2

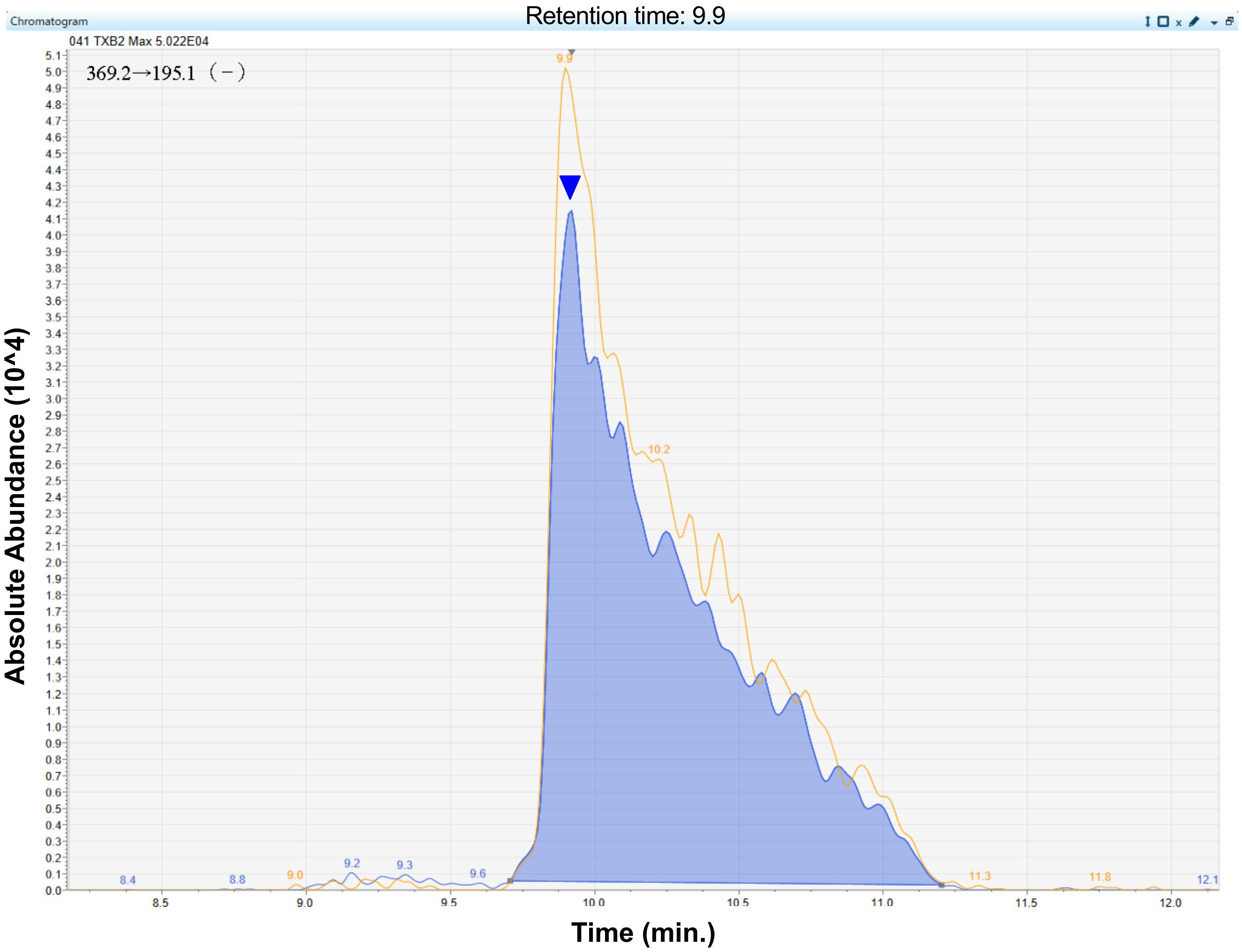
Retention time: 9.9
Chromatogram
041 TXB2 Max 5.022E04
369.2→195.1 (−)
Absolute Abundance (10^4)
Time (min.)
9.9
10.2
8.4
8.8
9.0
9.2
9.3
9.6
11.3
11.8
12.1

046 PGD_3

Retention time: 10.2

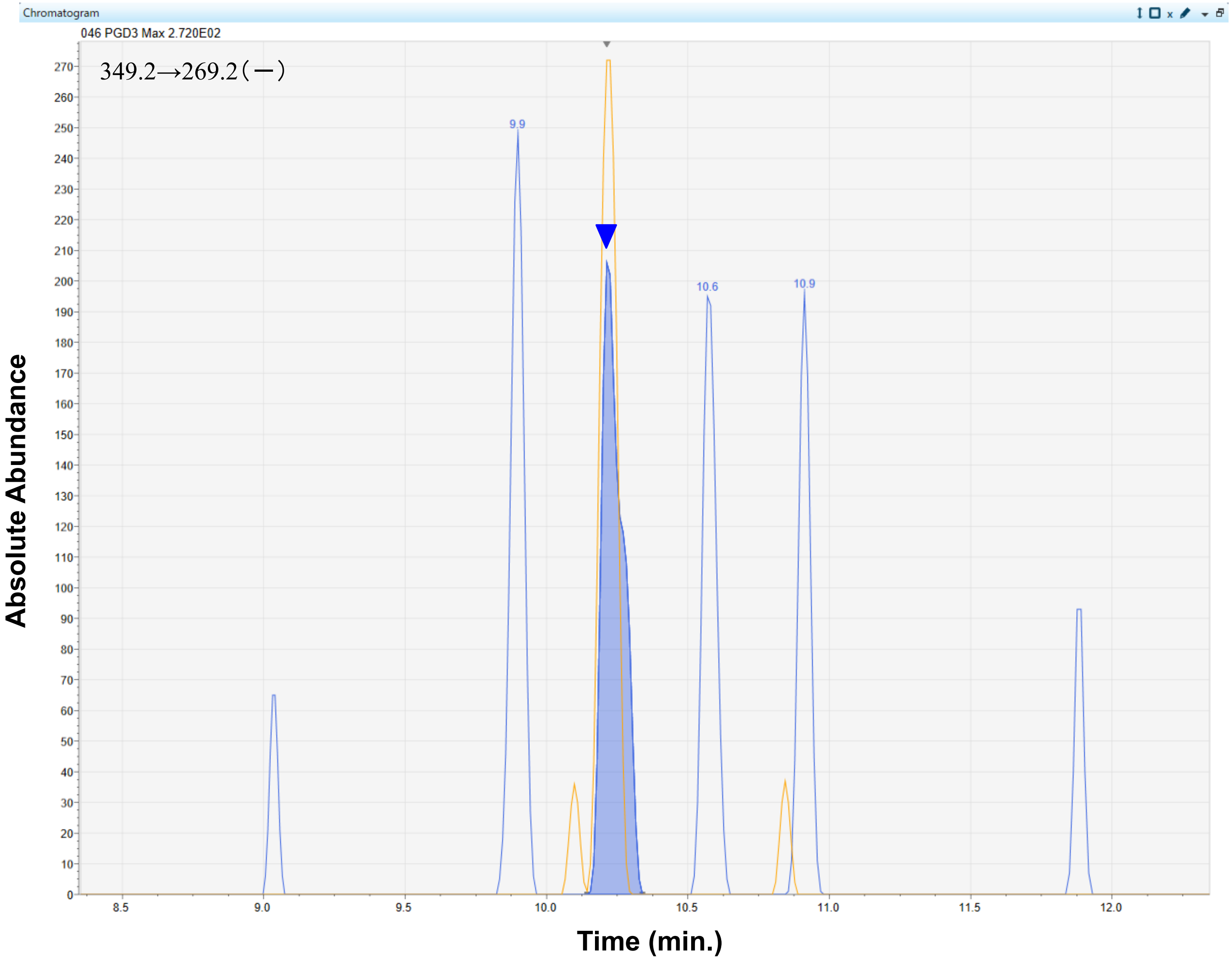

048 PGF$_{2\alpha}$-d_4 (IS)

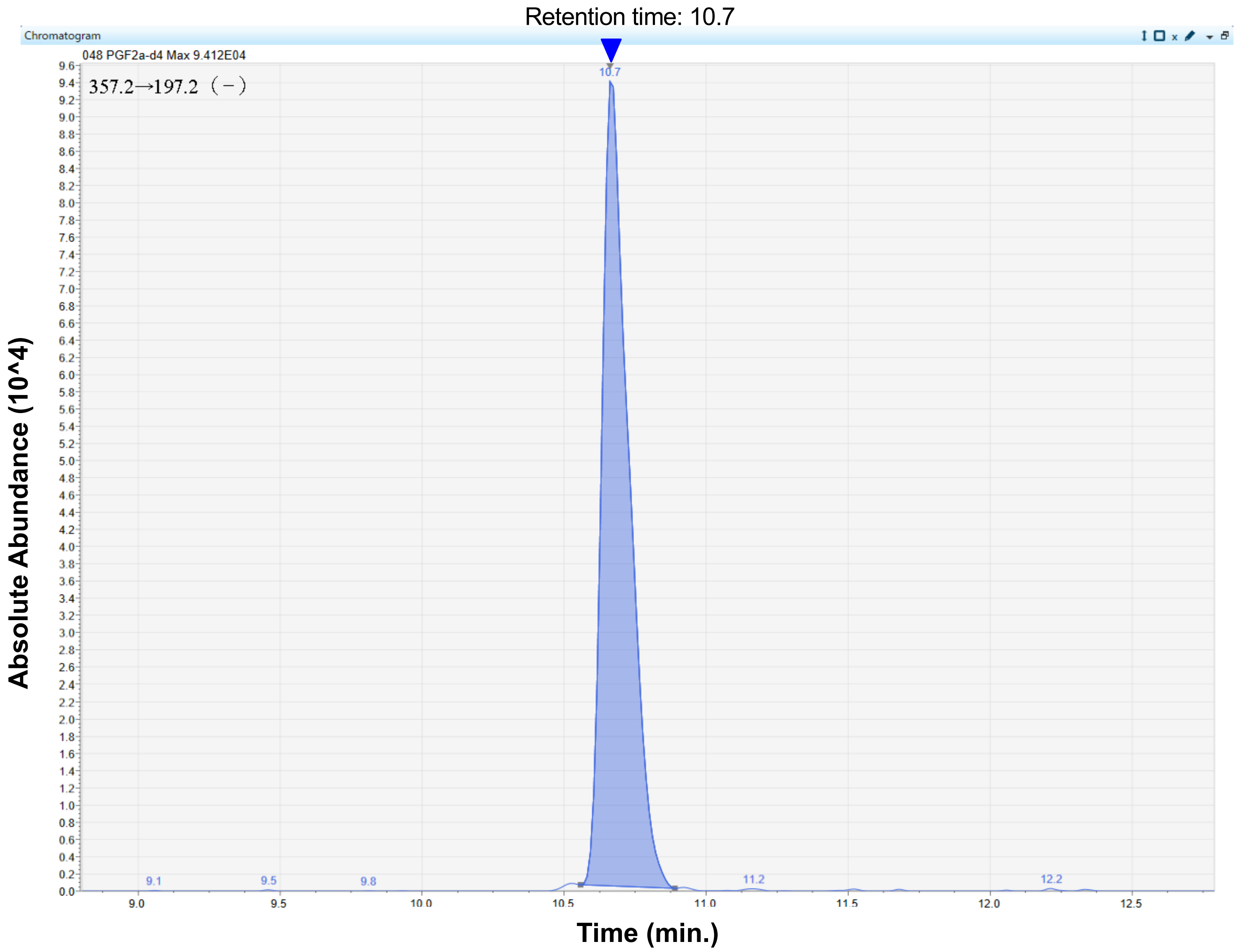

049 $PGF_{2\alpha}$

Retention time: 10.7

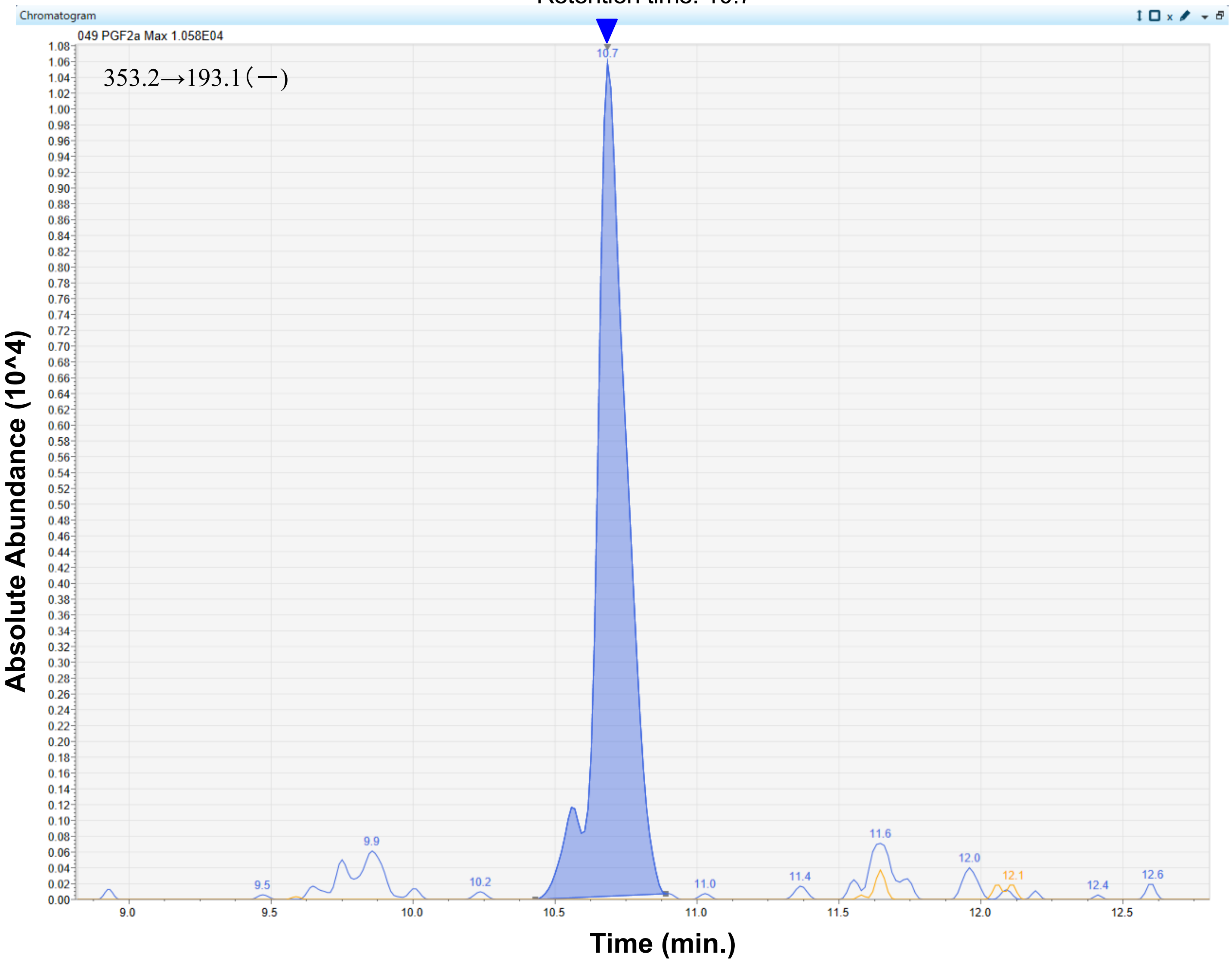

050 $PGF_{1\alpha}$

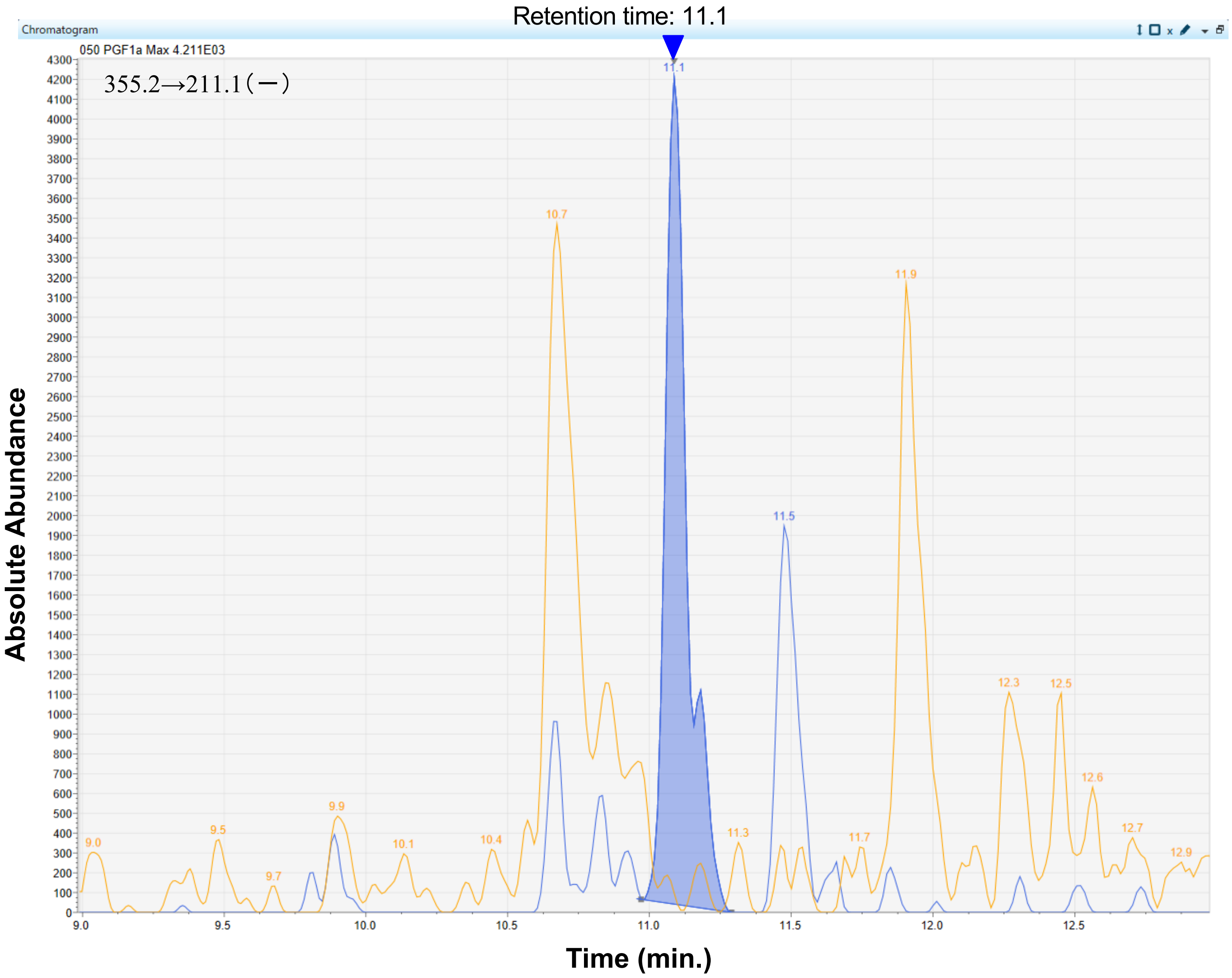

054 PGE_2-d_4 (IS)

Retention time: 11.1

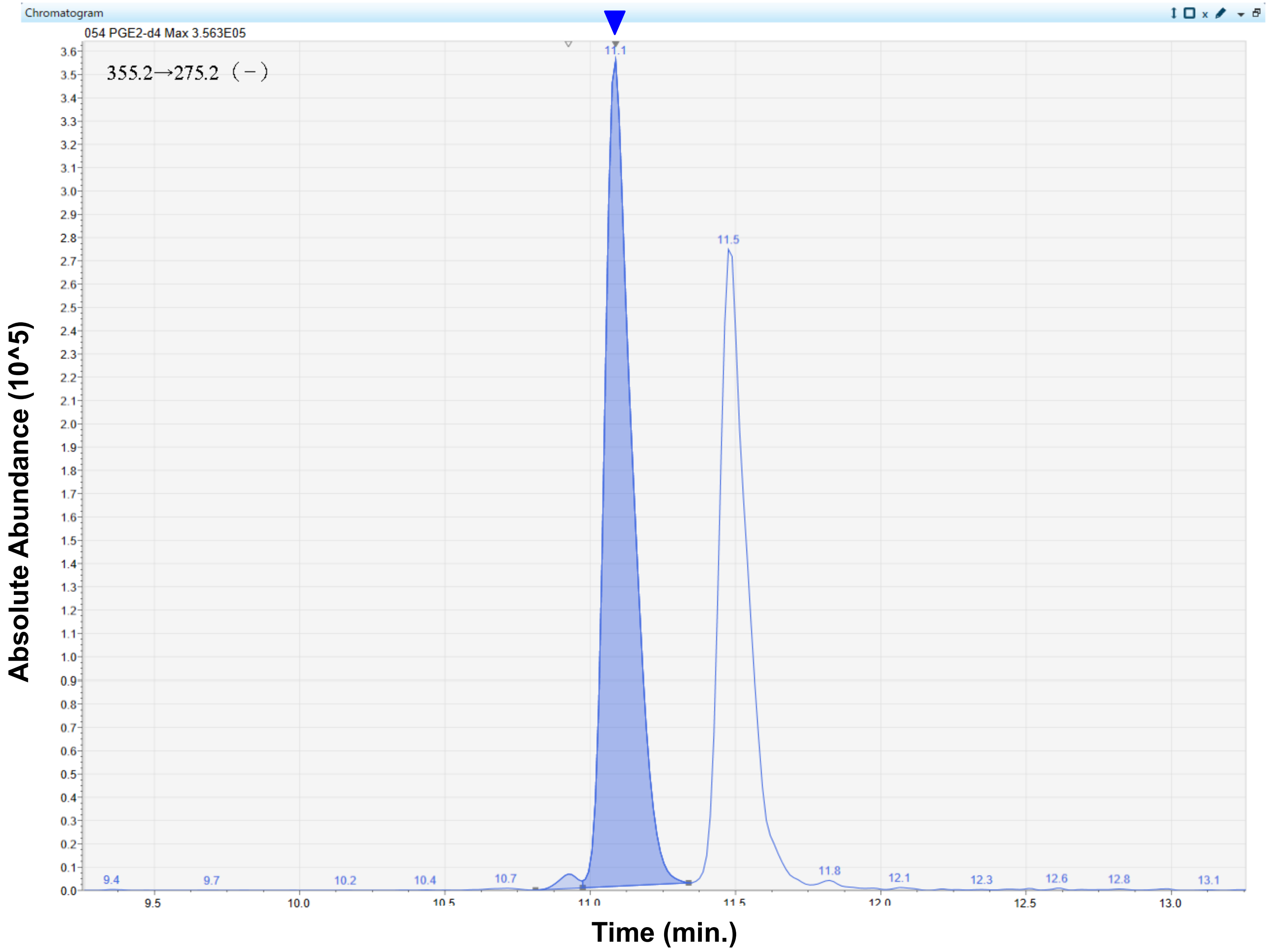

055 PGE_2

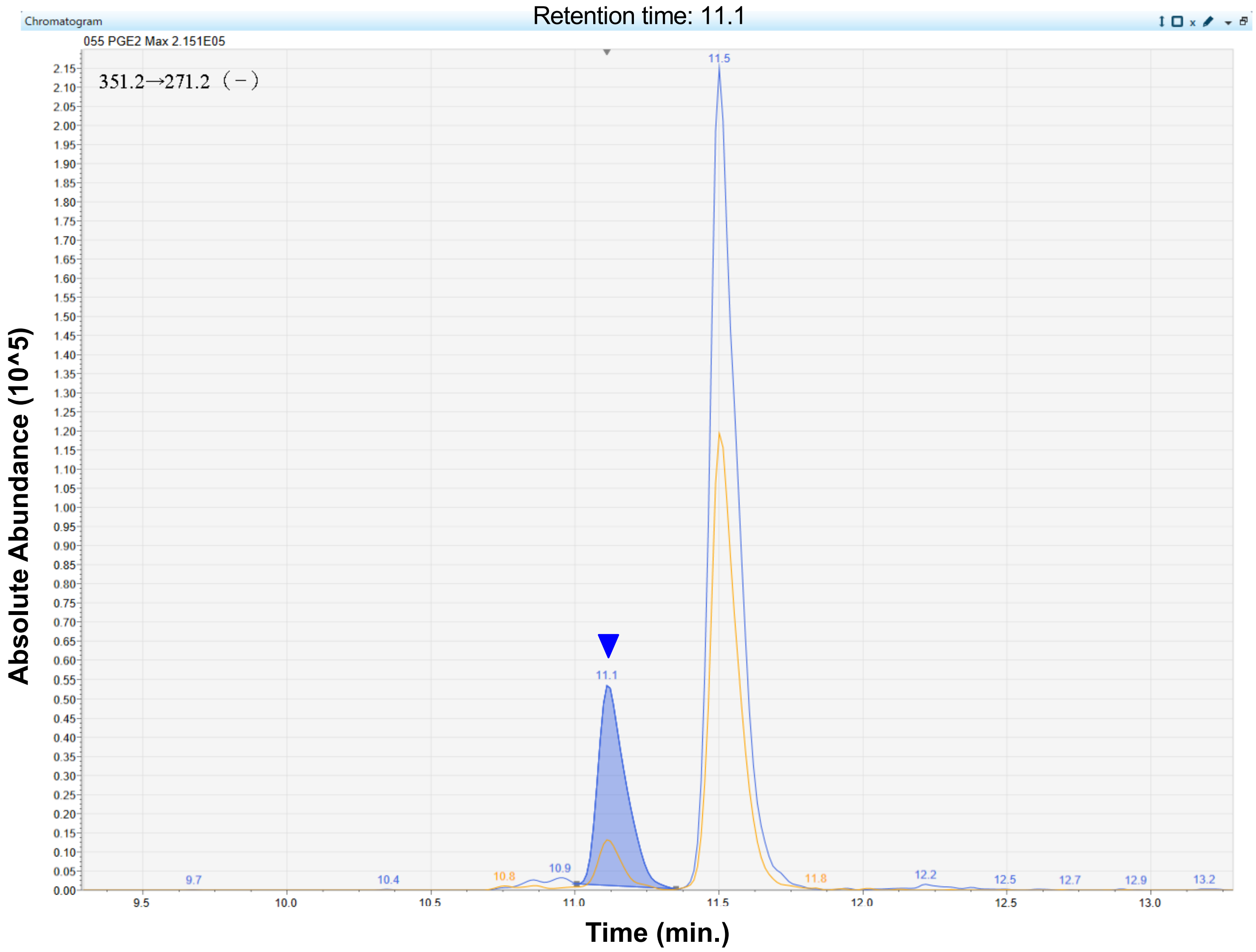
Retention time: 11.1
Chromatogram
055 PGE2 Max 2.151E05
351.2→271.2 (−)
Absolute Abundance (10^5)
Time (min.)
11.1
11.5
10.8
10.9
9.7
10.4
11.8
12.2
12.5
12.7
12.9
13.2
2.15
2.10
2.05
2.00
1.95
1.90
1.85
1.80
1.75
1.70
1.65
1.60
1.55
1.50
1.45
1.40
1.35
1.30
1.25
1.20
1.15
1.10
1.05
1.00
0.95
0.90
0.85
0.80
0.75
0.70
0.65
0.60
0.55
0.50
0.45
0.40
0.35
0.30
0.25
0.20
0.15
0.10
0.05
0.00
9.5
10.0
10.5
11.0
11.5
12.0
12.5
13.0

063 PGD_2-d_4 (IS)

Retention time: 11.5

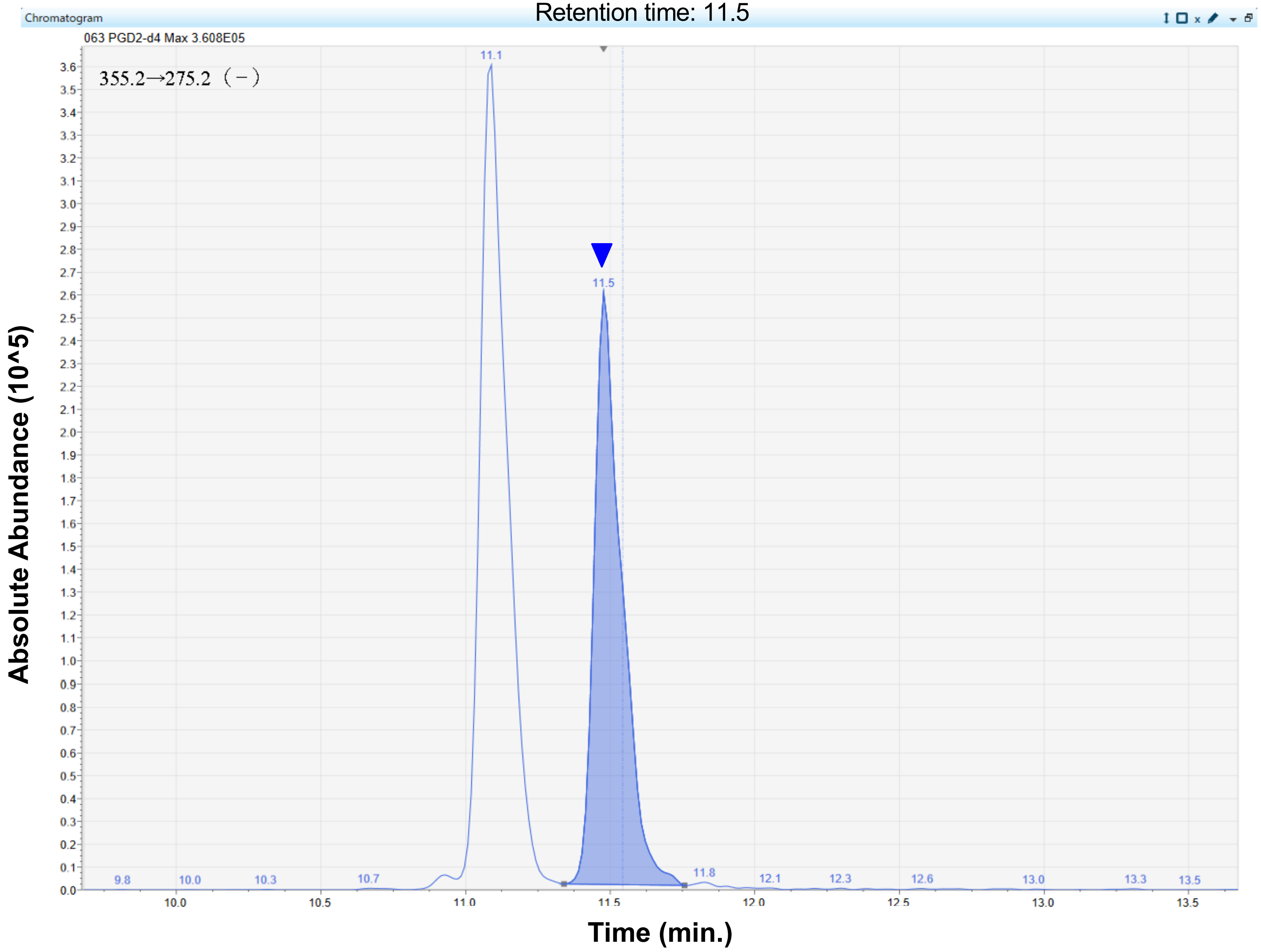

064 PGE_1

Retention time: 11.6

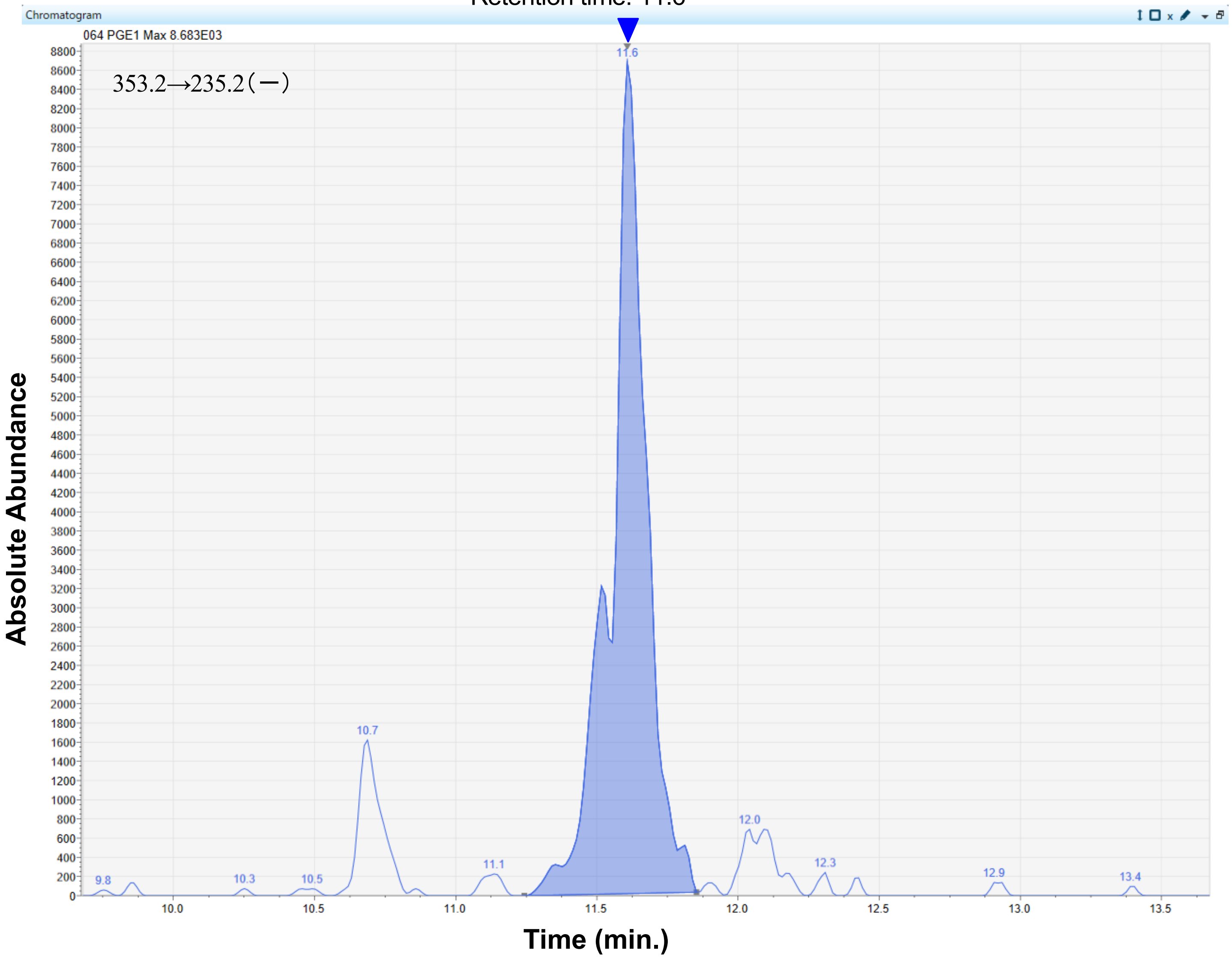

065 PGD_2

Retention time: 11.5

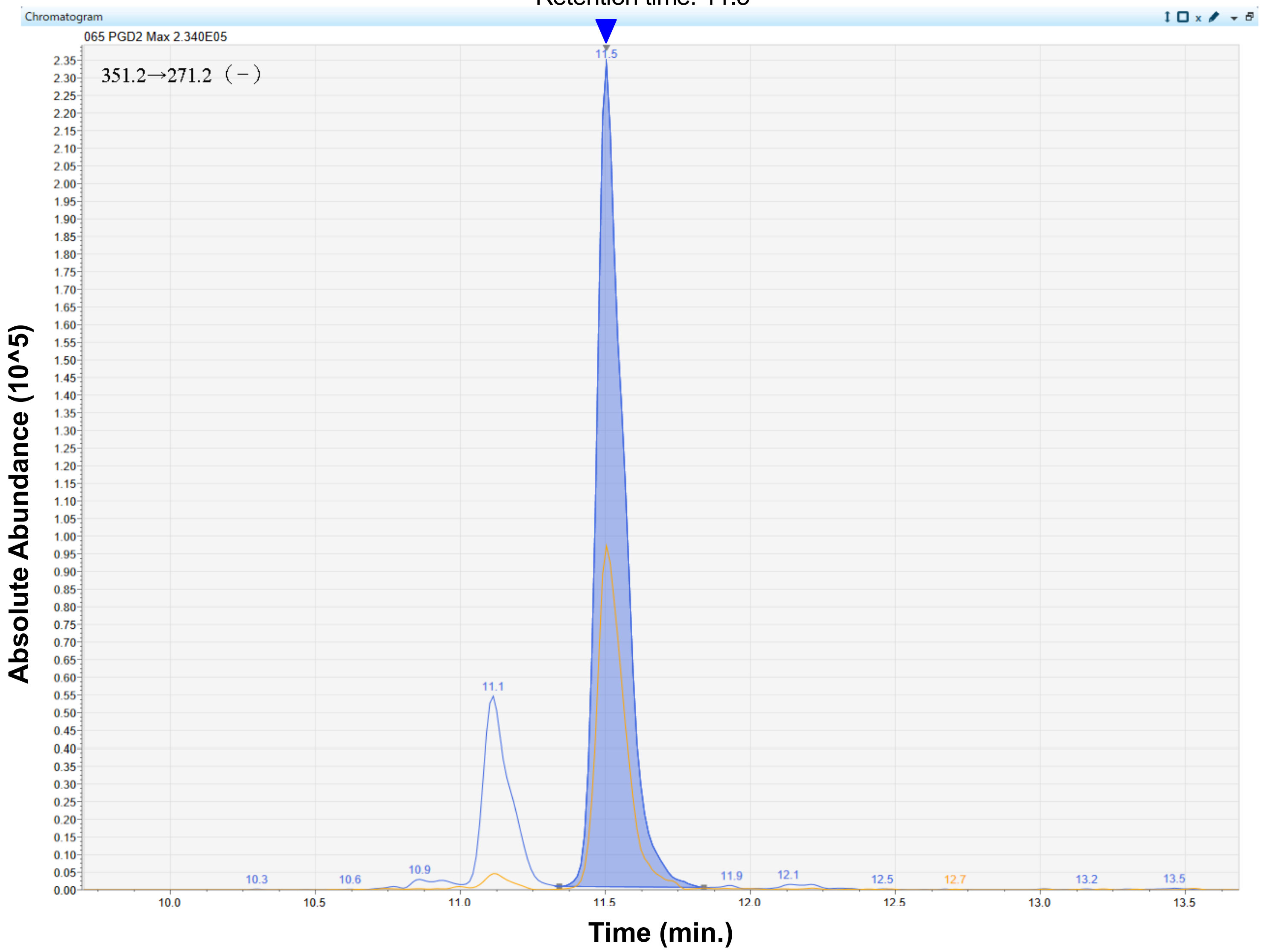

065 PGD_1

Retention time: 11.6

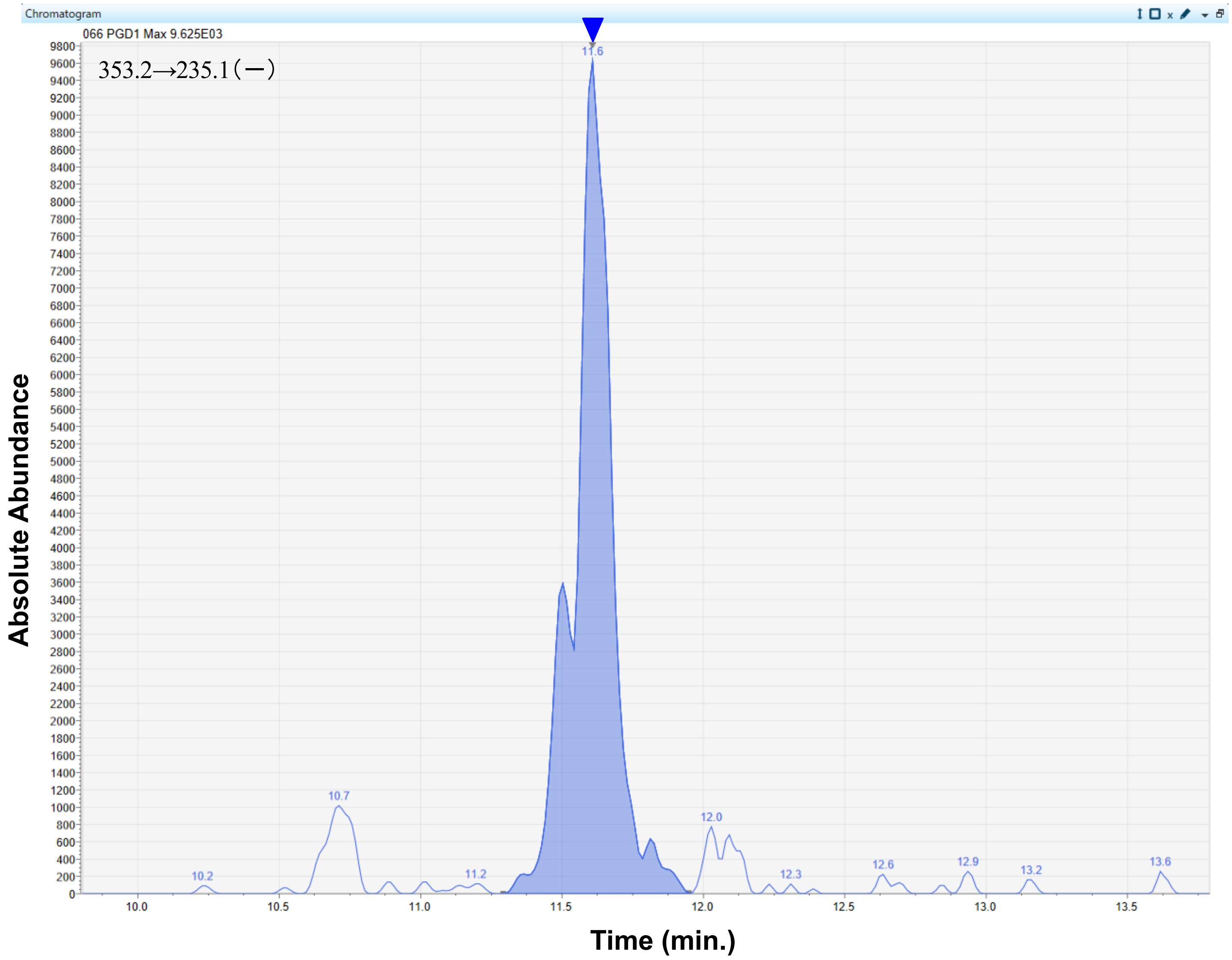

073 14,15-LTC_4

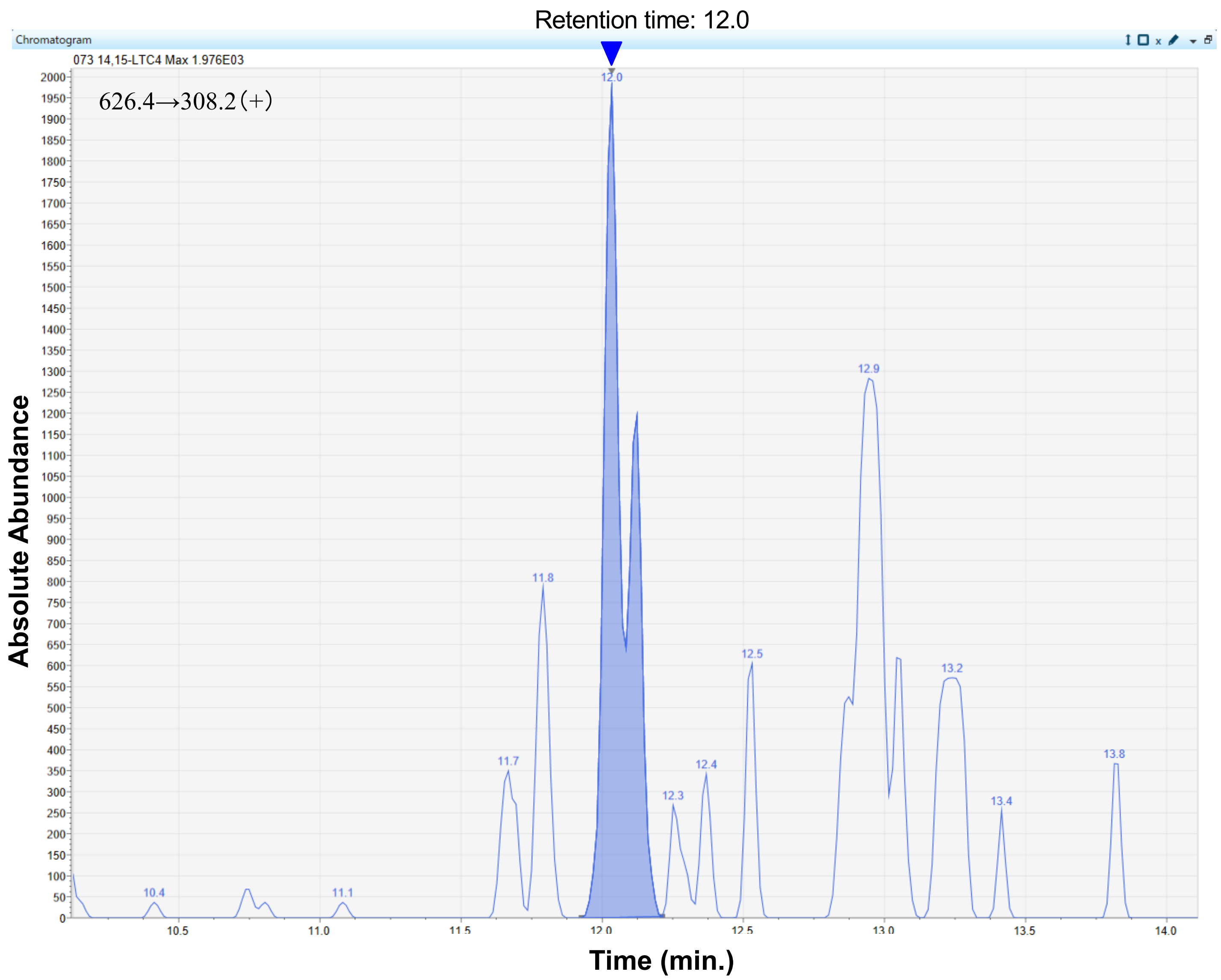

082 LTB_4-EA

Retention time: 13.3

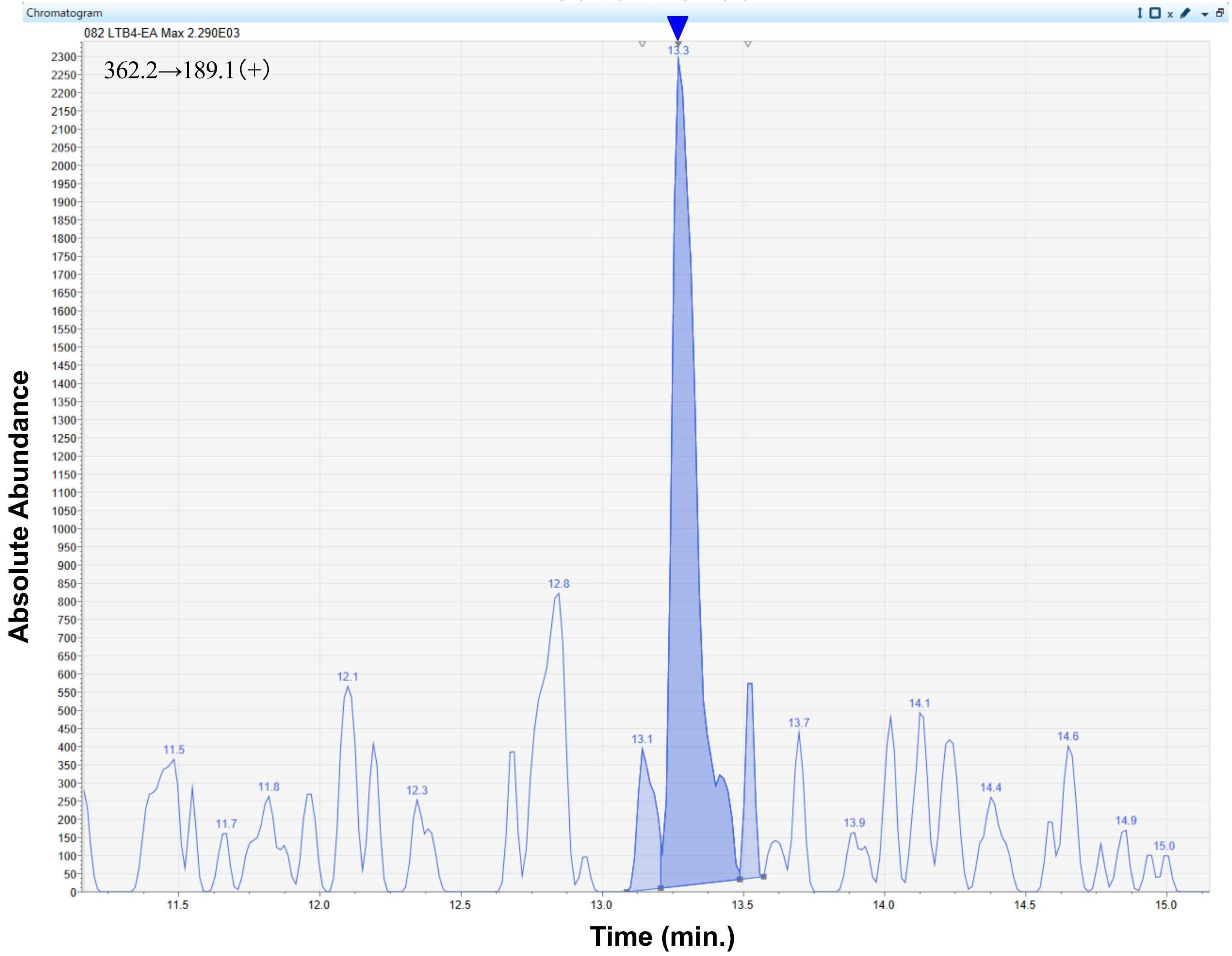

083 LTC_4-*d_5* (IS)

Retention time: 12.9

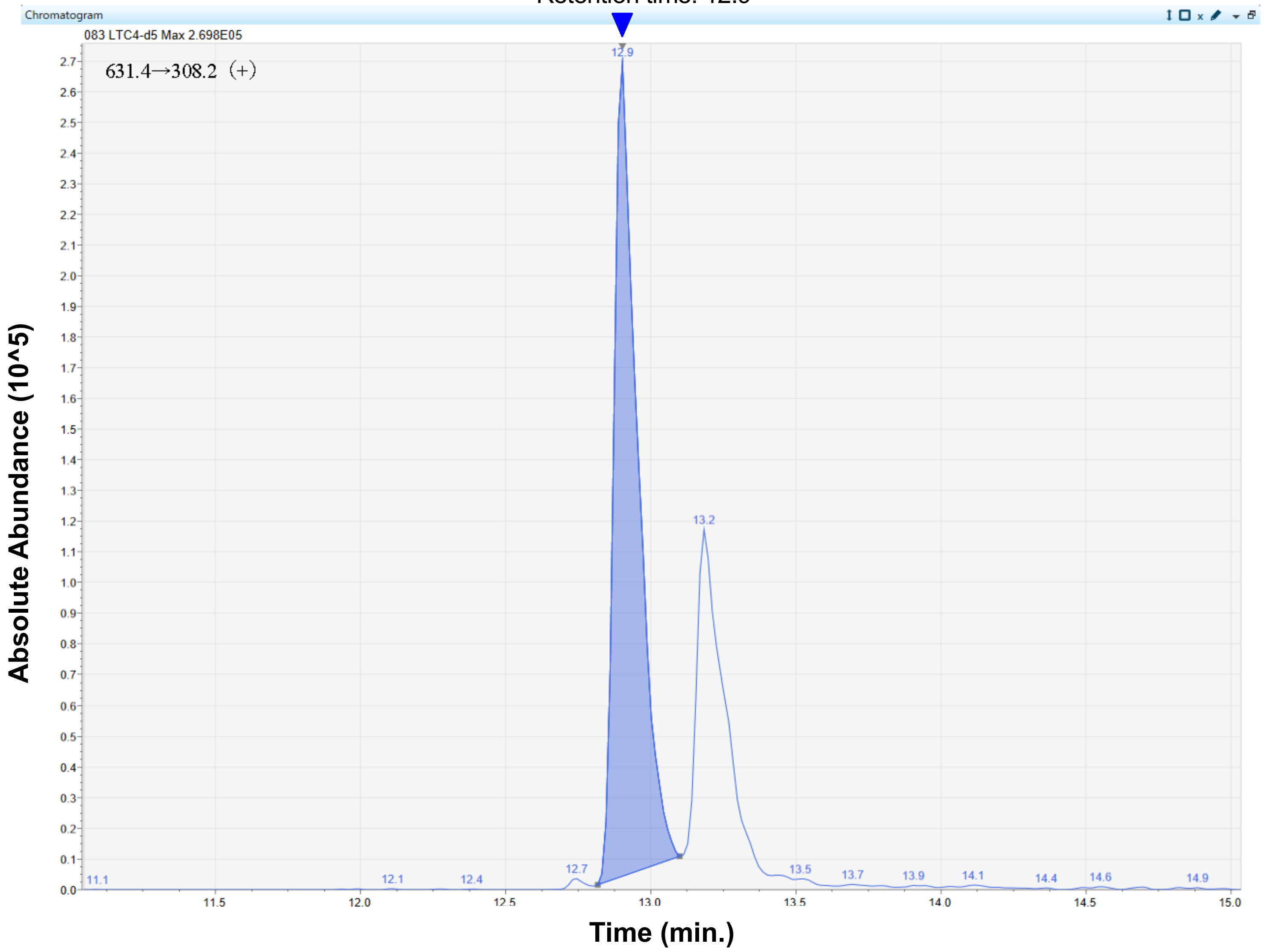

085 11-*trans*-LTC_4

Retention time: 12.9

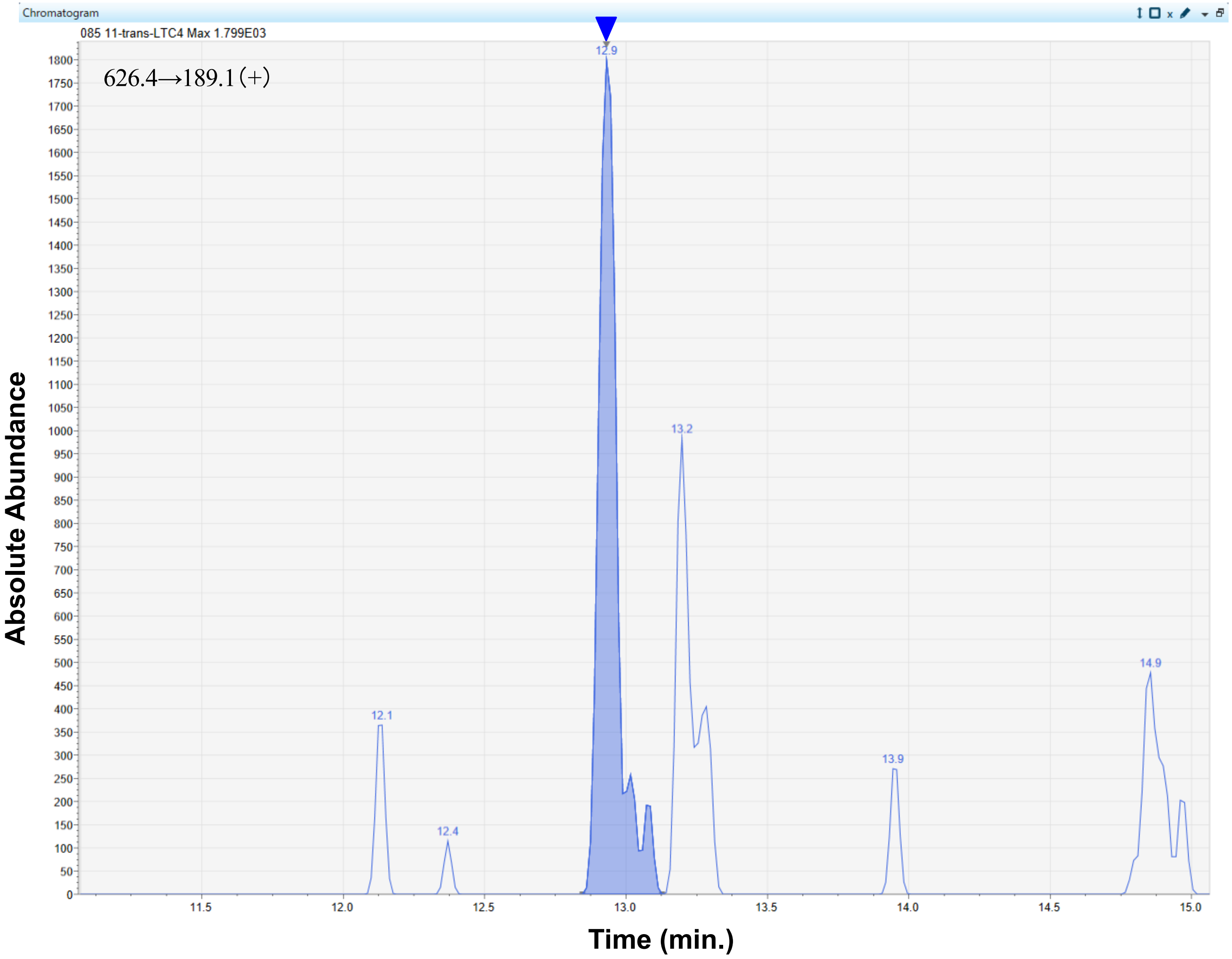

086 LTD_4-d_5 (IS)

Retention time: 12.8

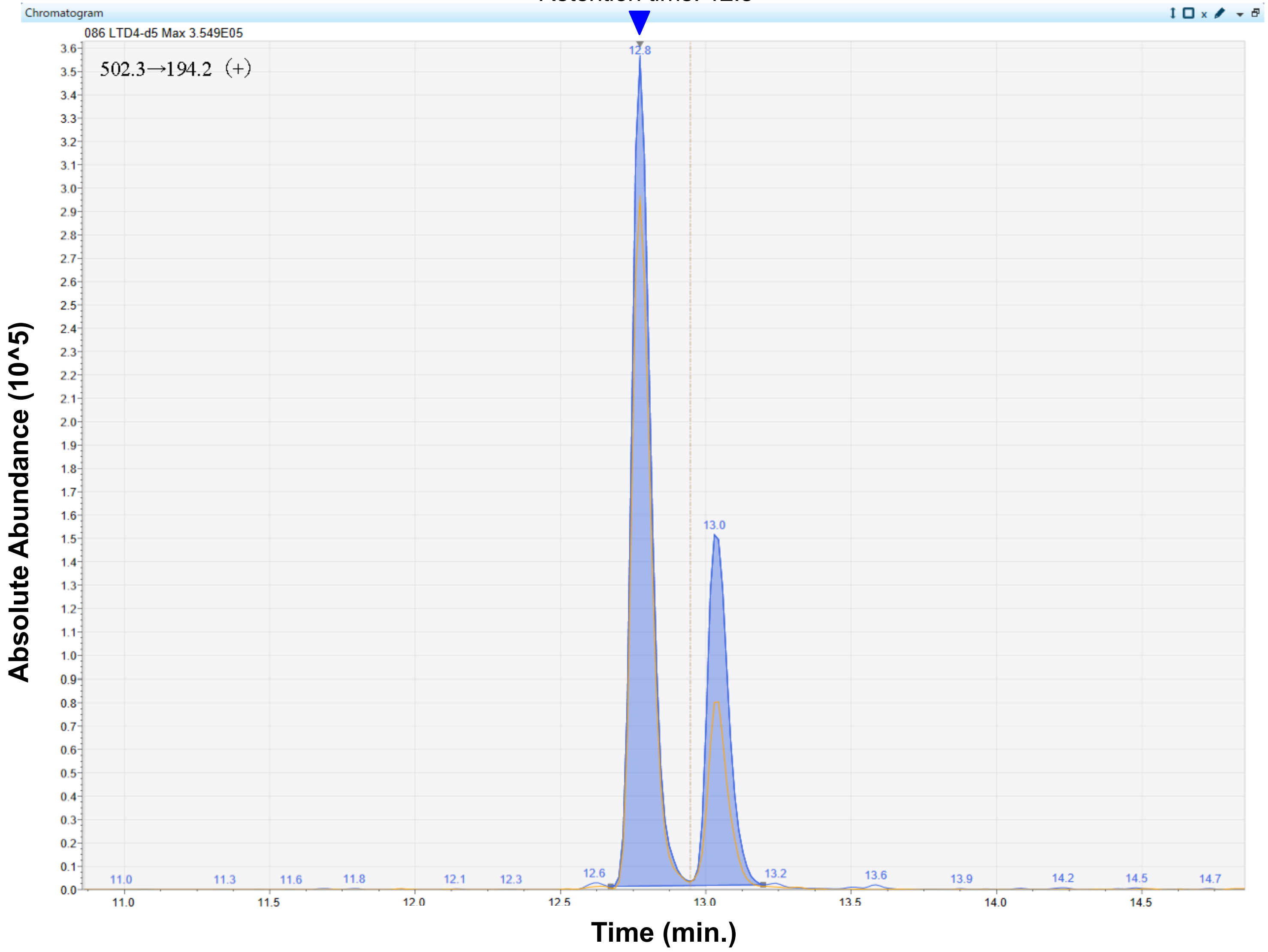

094 8-*iso*-PGA_1

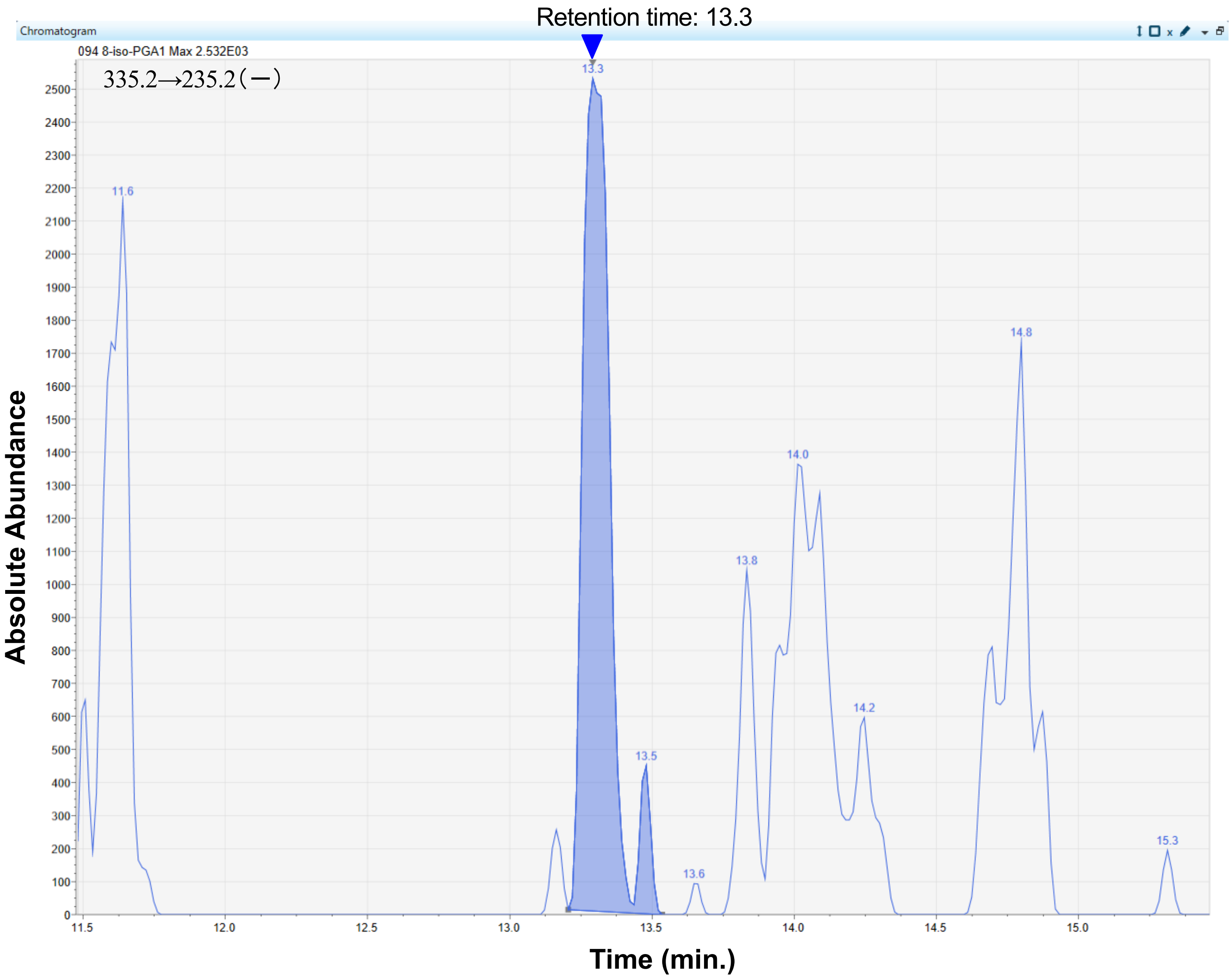

095 PGA_2-d_4 (IS)

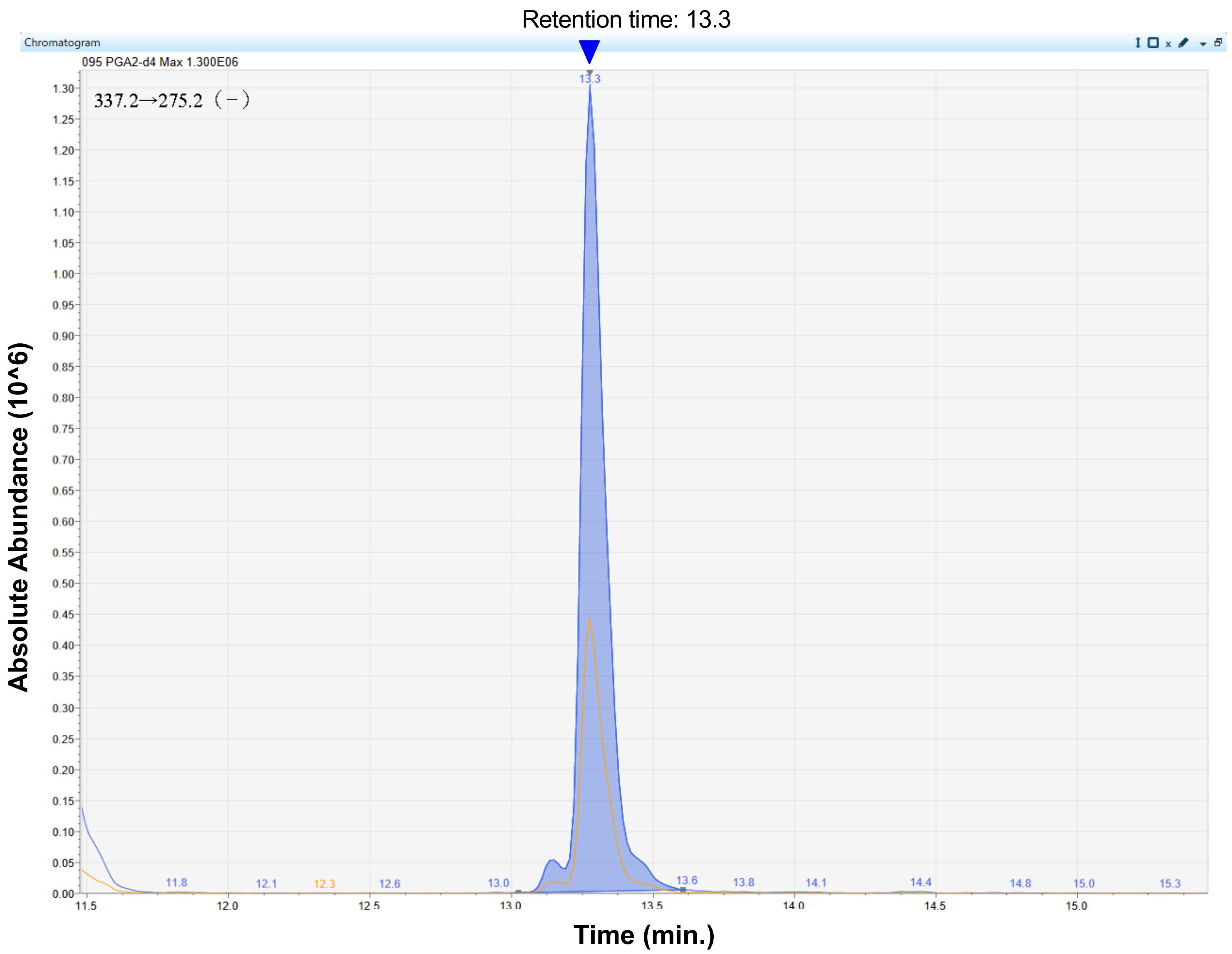

096 PGA_2

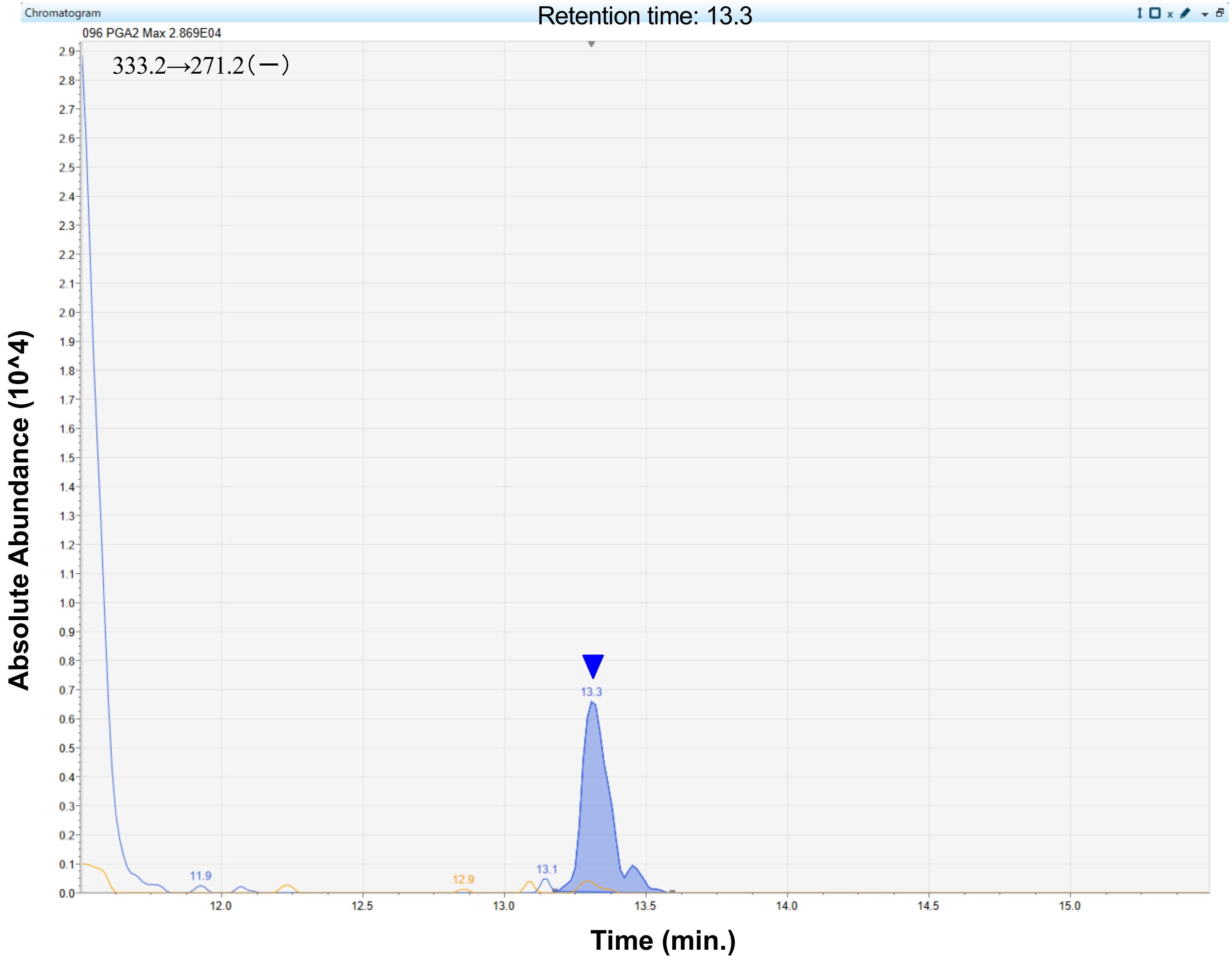
Chromatogram
Retention time: 13.3
096 PGA2 Max 2.869E04
333.2→271.2 (−)
Absolute Abundance (10^4)
13.3
13.1
11.9
12.9
Time (min.)

100 PGB_2

Retention time: 13.3

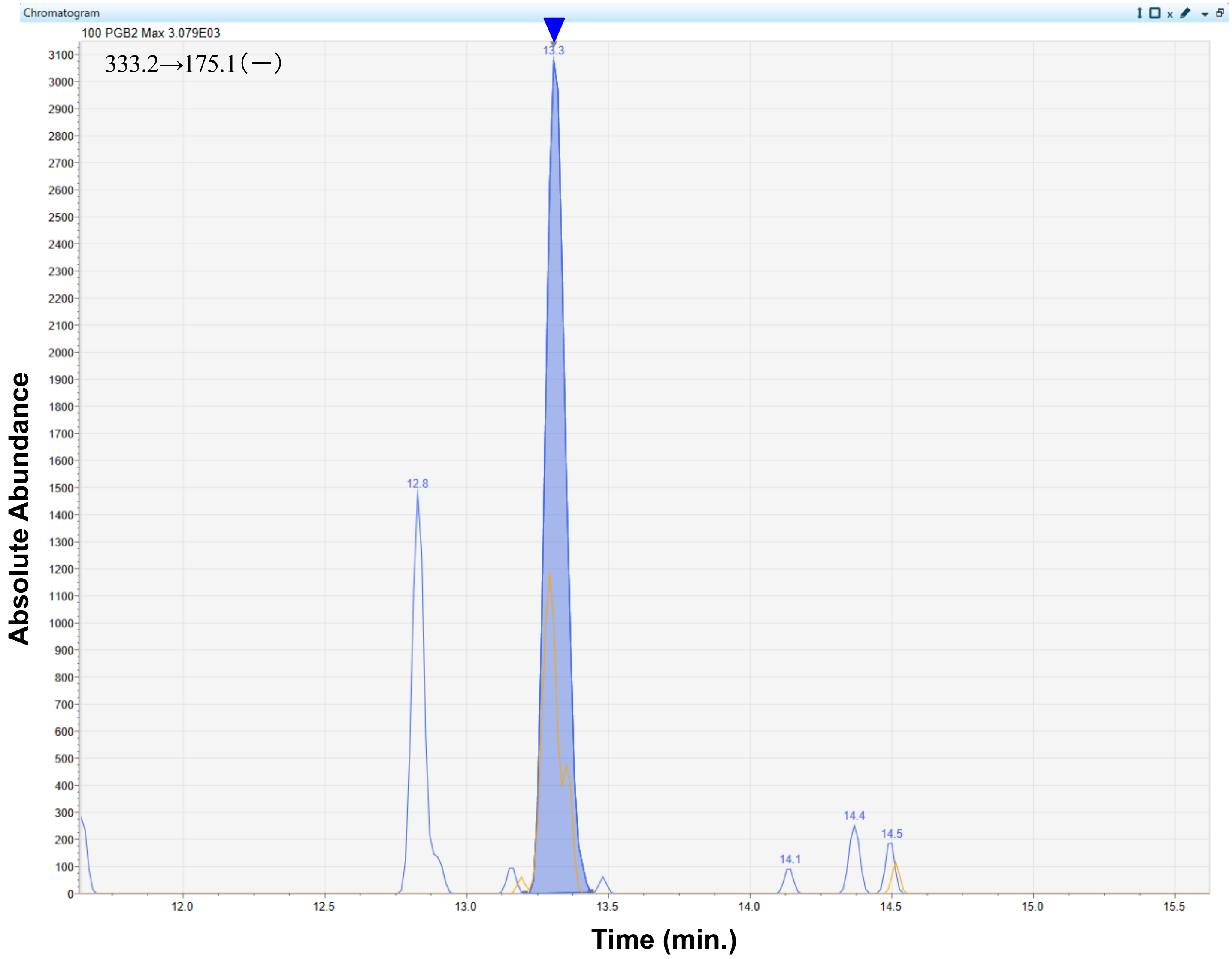

105 6-*trans*-LTB$_4$

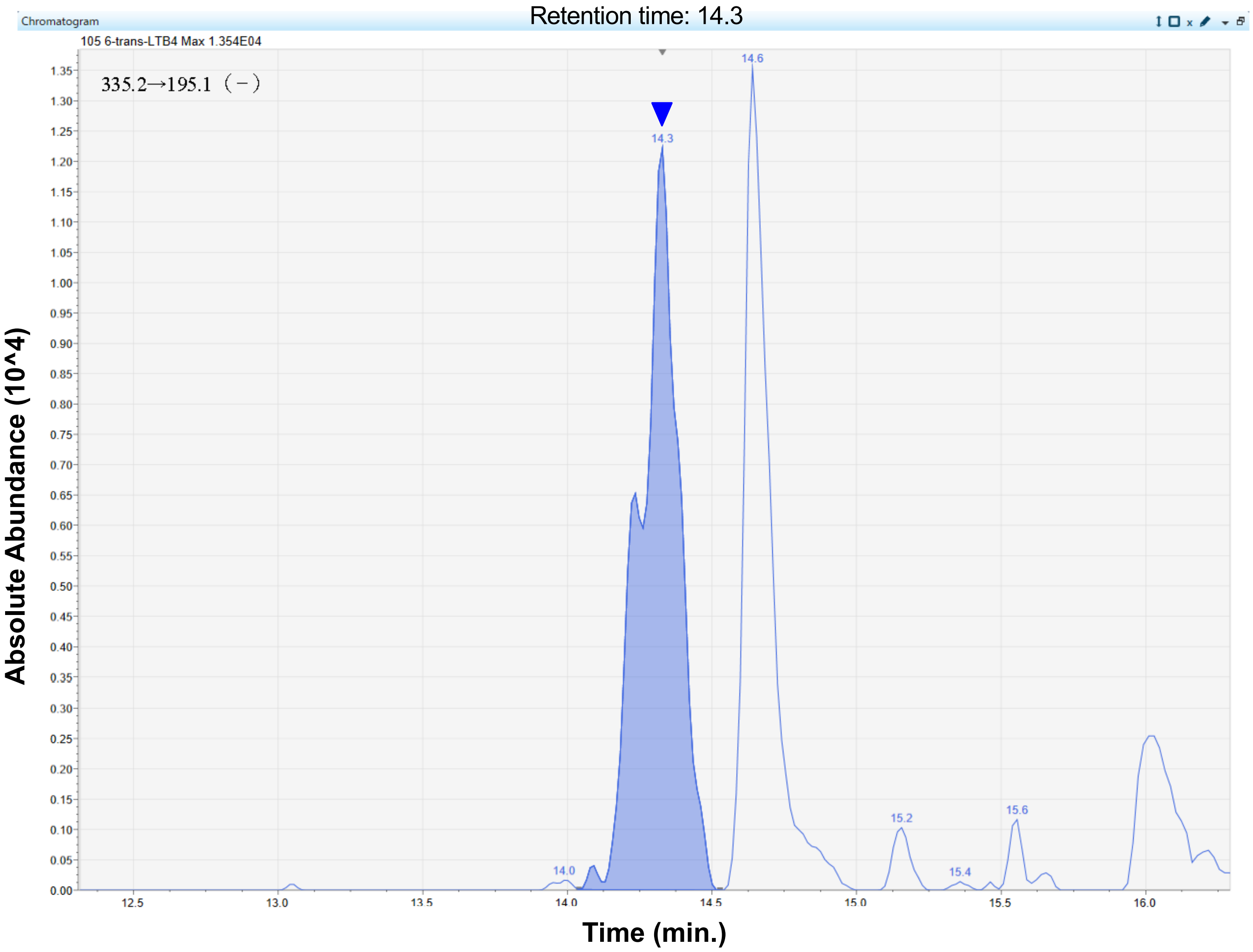

106 5,15-DiHETE

Retention time: 14.2

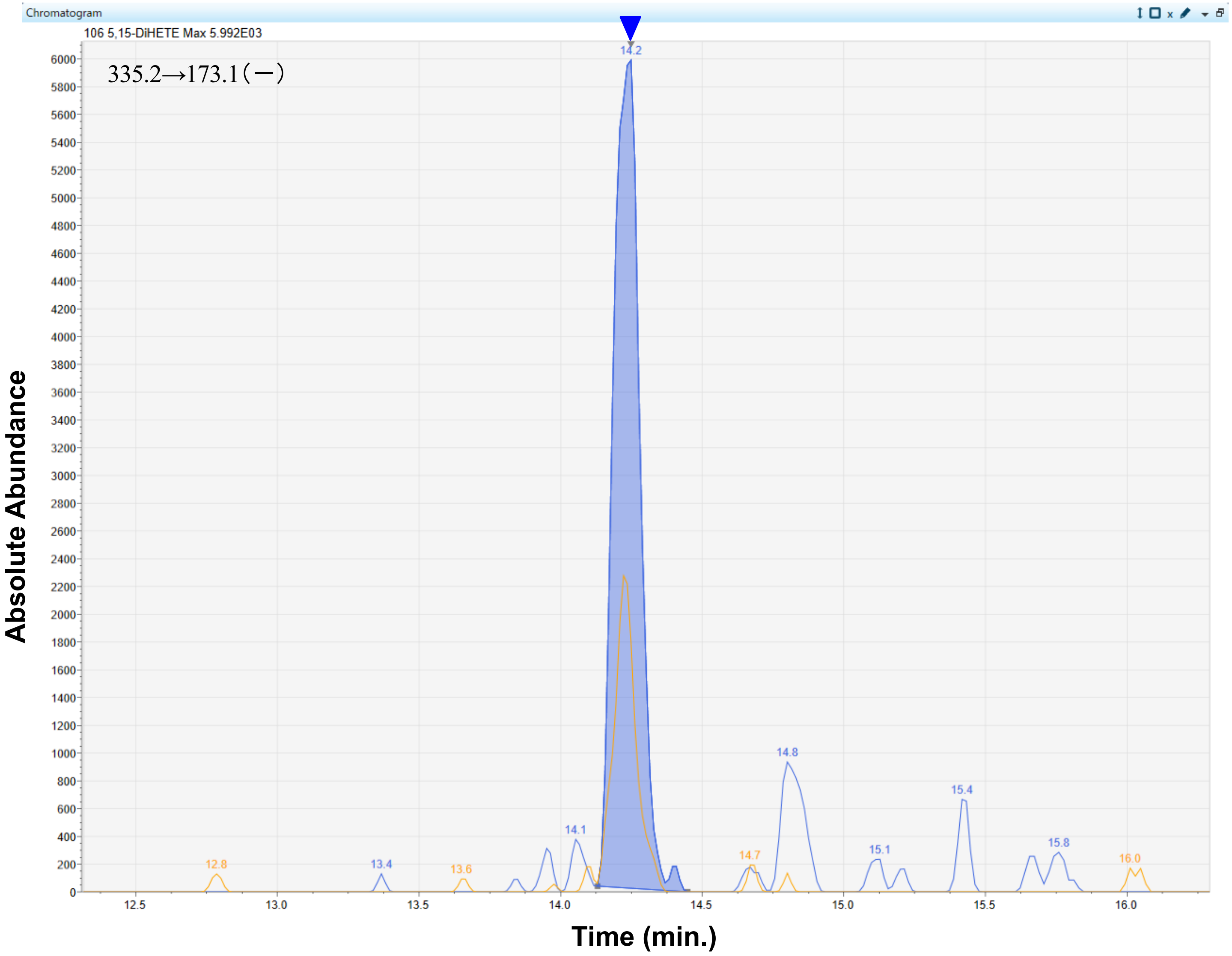

109 LTB_4-d_4 (IS)

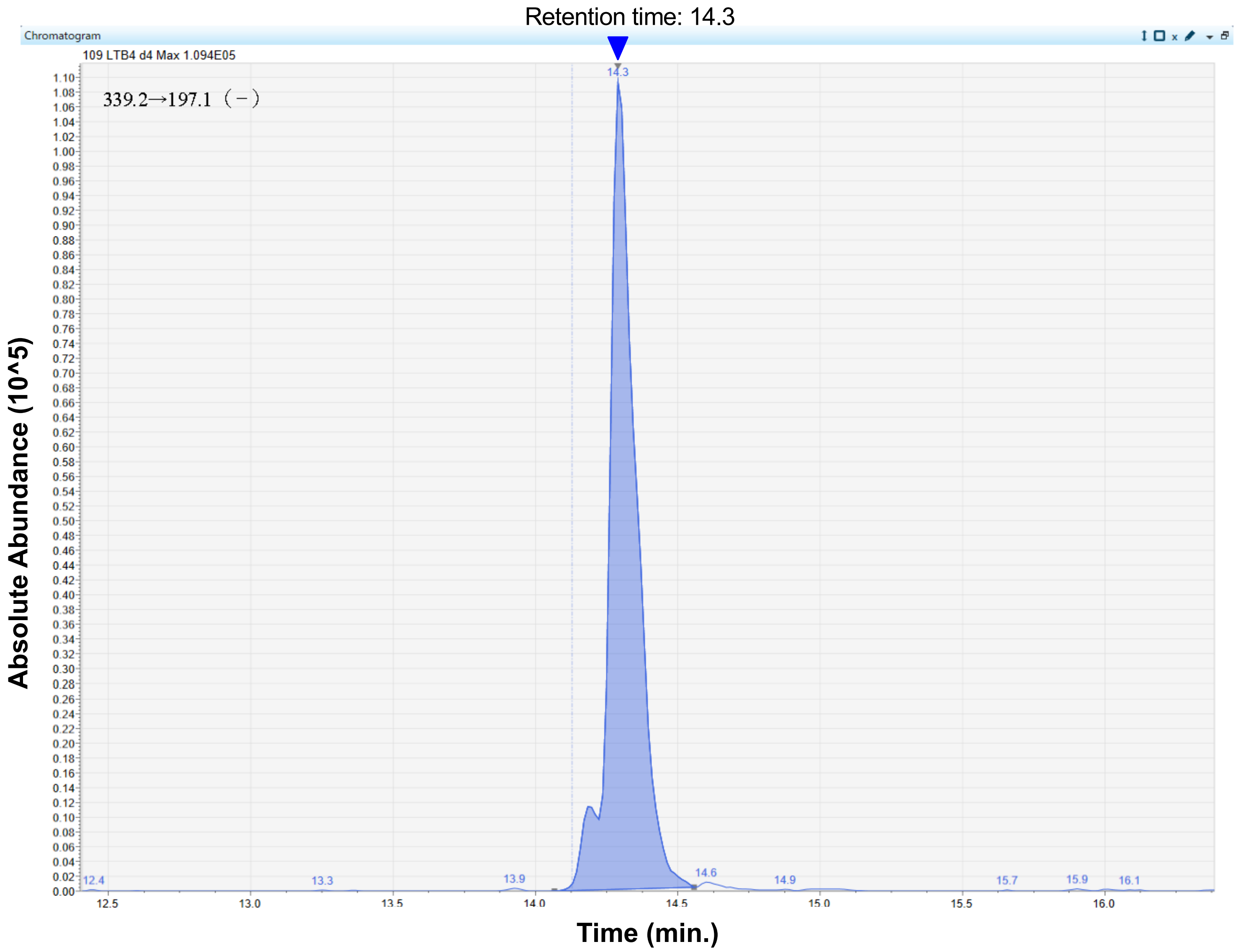

110 LTB_4

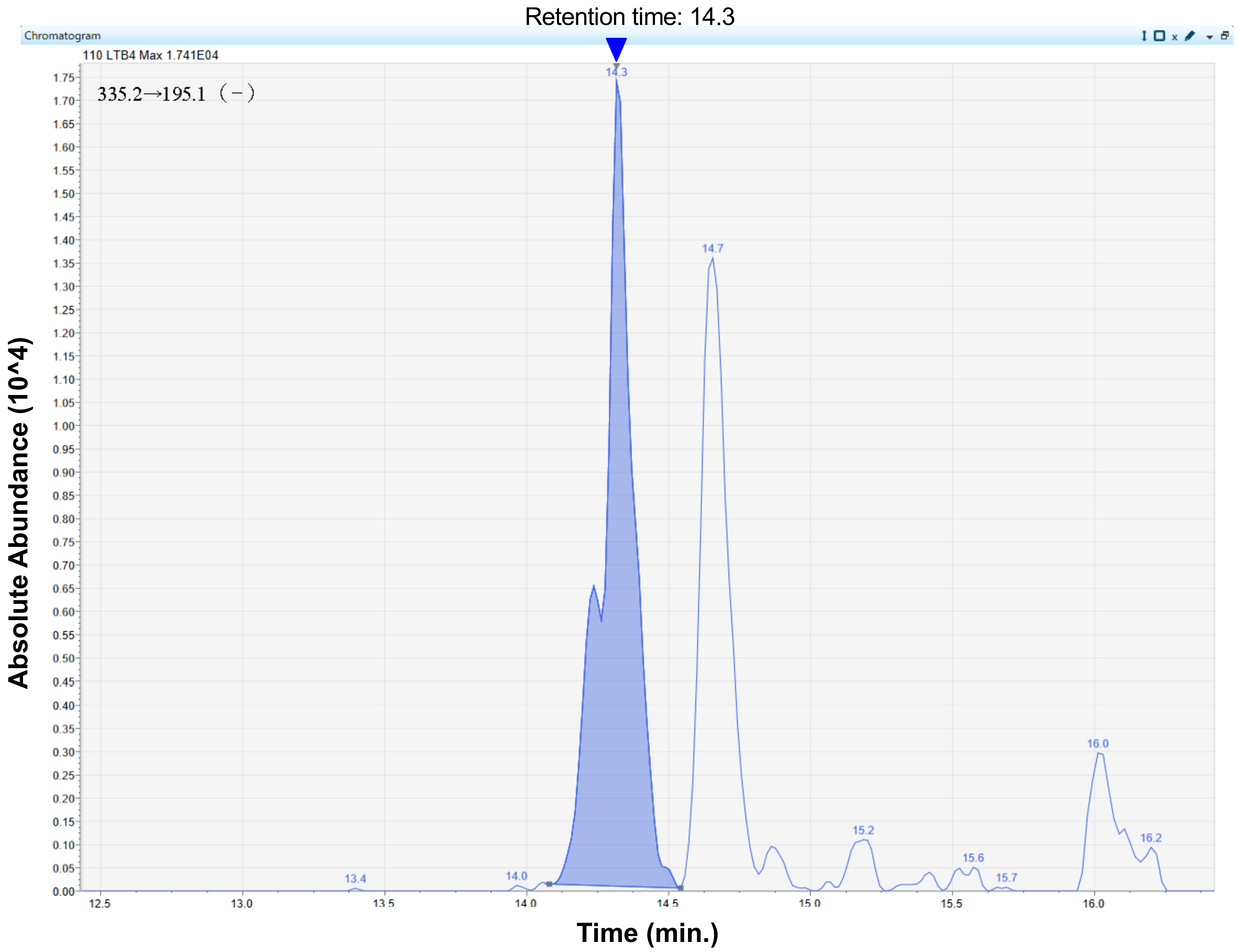
Retention time: 14.3
Chromatogram
110 LTB4 Max 1.741E04
335.2→195.1 (−)
14.3
14.7
13.4
14.0
15.2
15.6
15.7
16.0
16.2
Absolute Abundance (10^4)
Time (min.)
1.75
1.70
1.65
1.60
1.55
1.50
1.45
1.40
1.35
1.30
1.25
1.20
1.15
1.10
1.05
1.00
0.95
0.90
0.85
0.80
0.75
0.70
0.65
0.60
0.55
0.50
0.45
0.40
0.35
0.30
0.25
0.20
0.15
0.10
0.05
0.00
12.5
13.0
13.5
14.0
14.5
15.0
15.5
16.0

120 tetranor-12-HETE

Retention time: 15.0

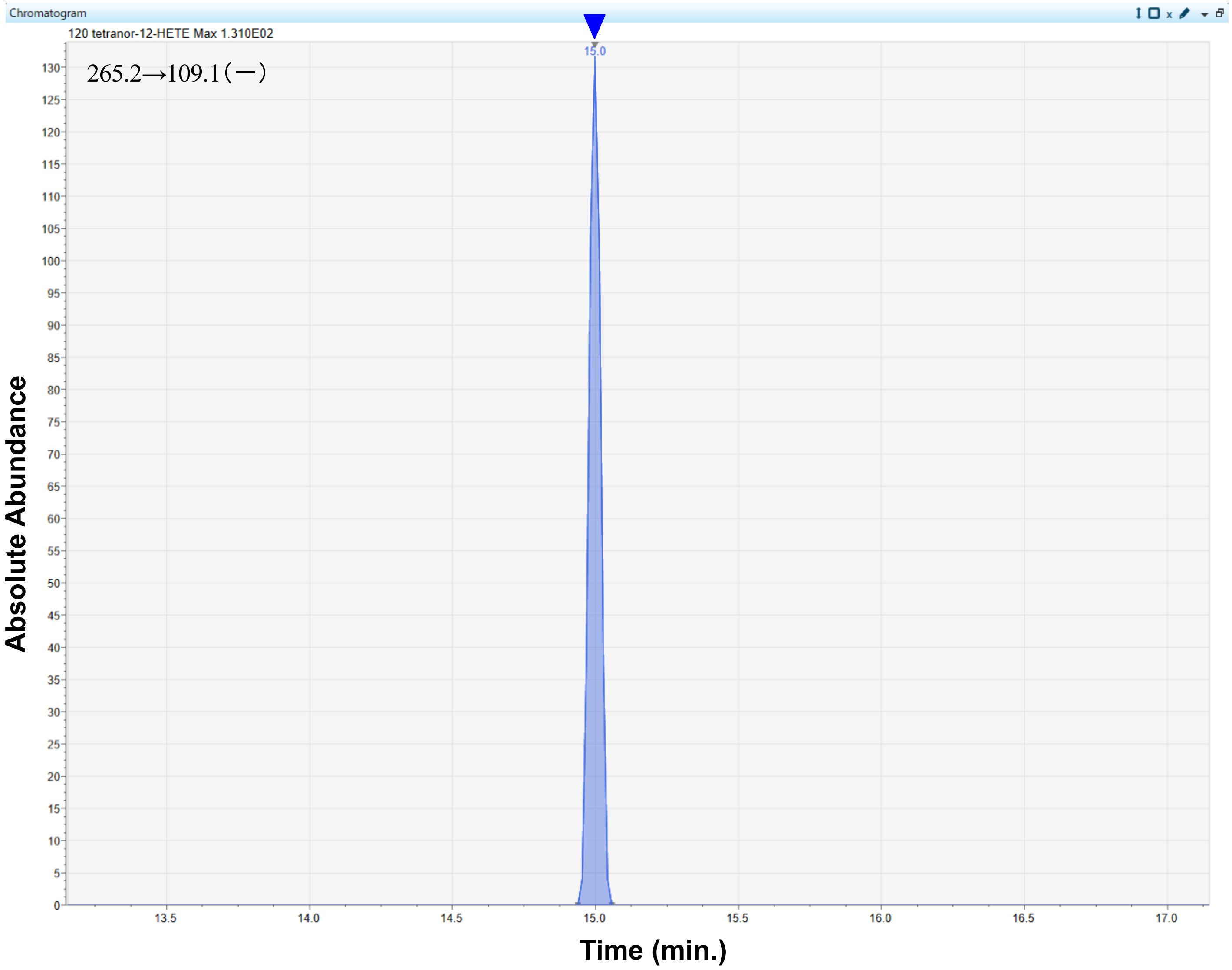

122 14,15-DiHET-d_{11} (IS)

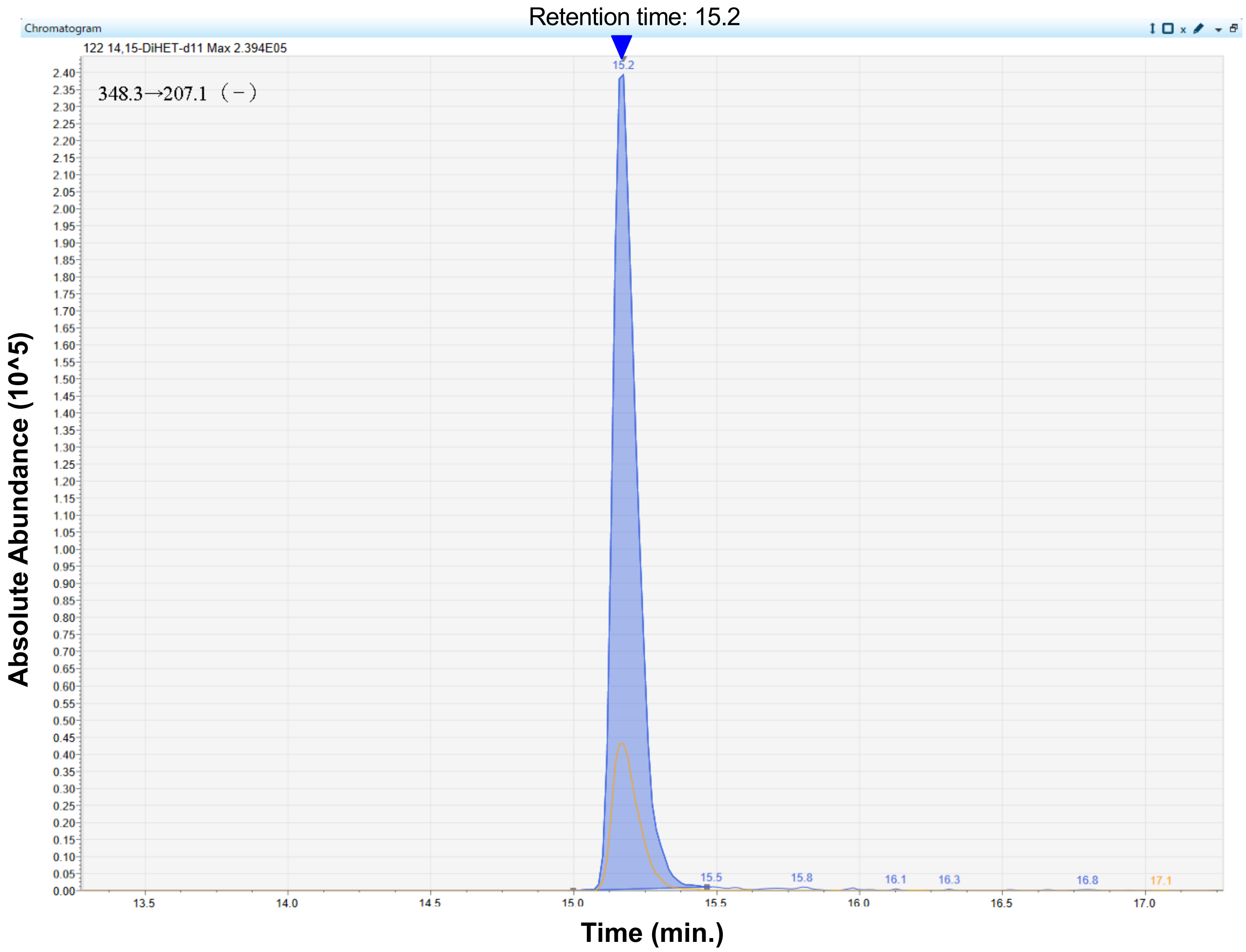

125 14,15-DHET

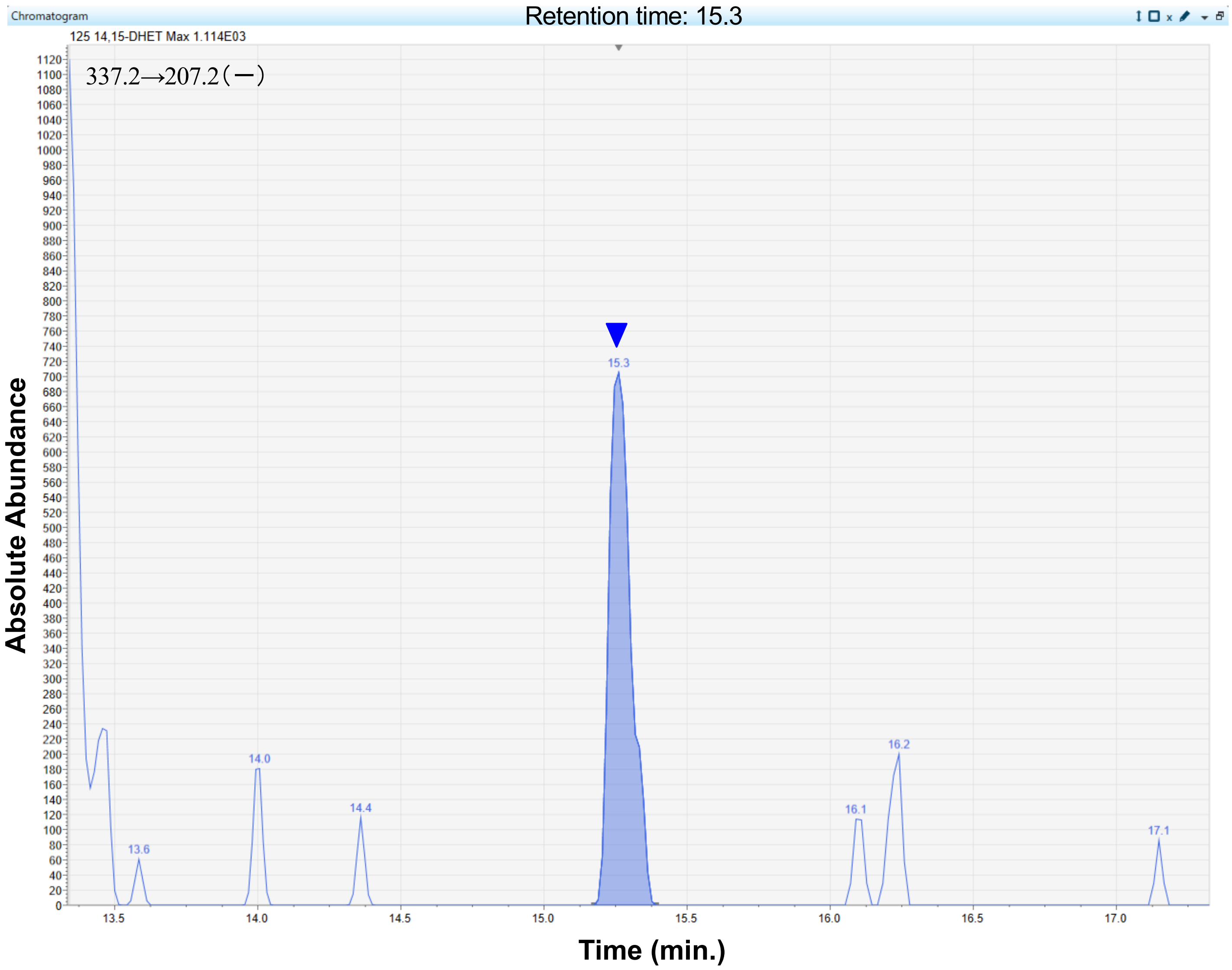

126 12-HHT

Retention time: 15.3

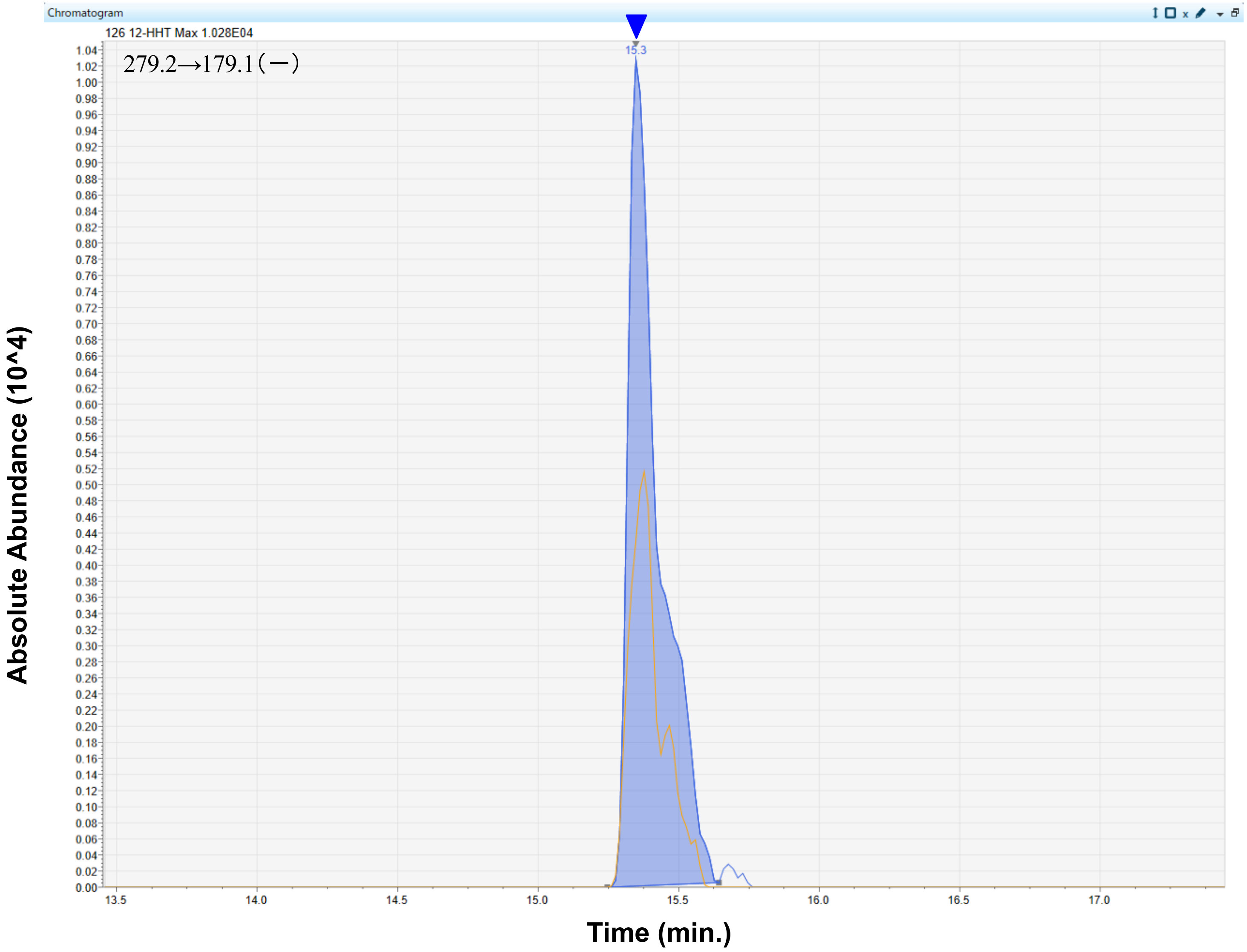

133 13-HOTrE

Retention time: 16.0

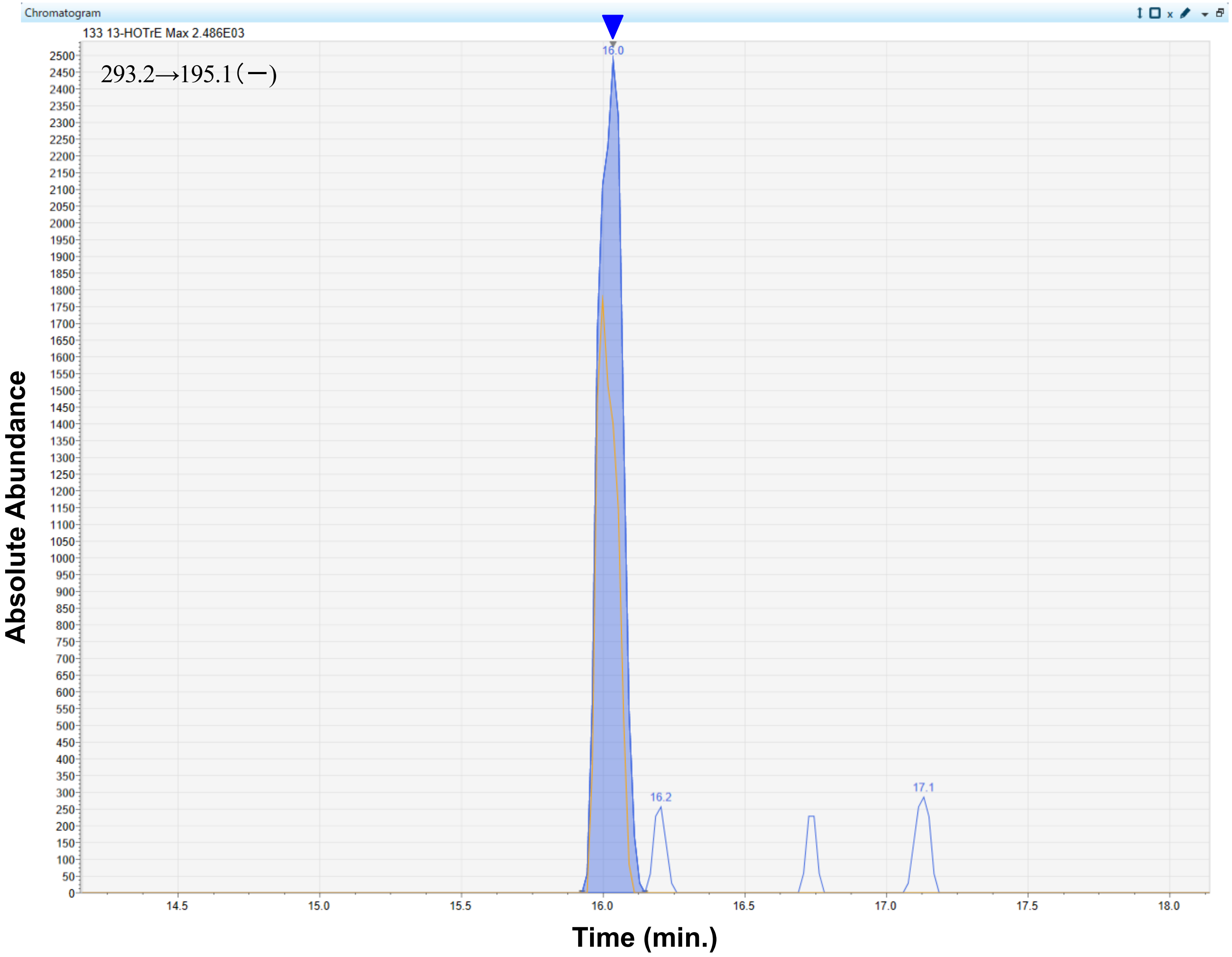

139 15-HEPE

Retention time: 16.4

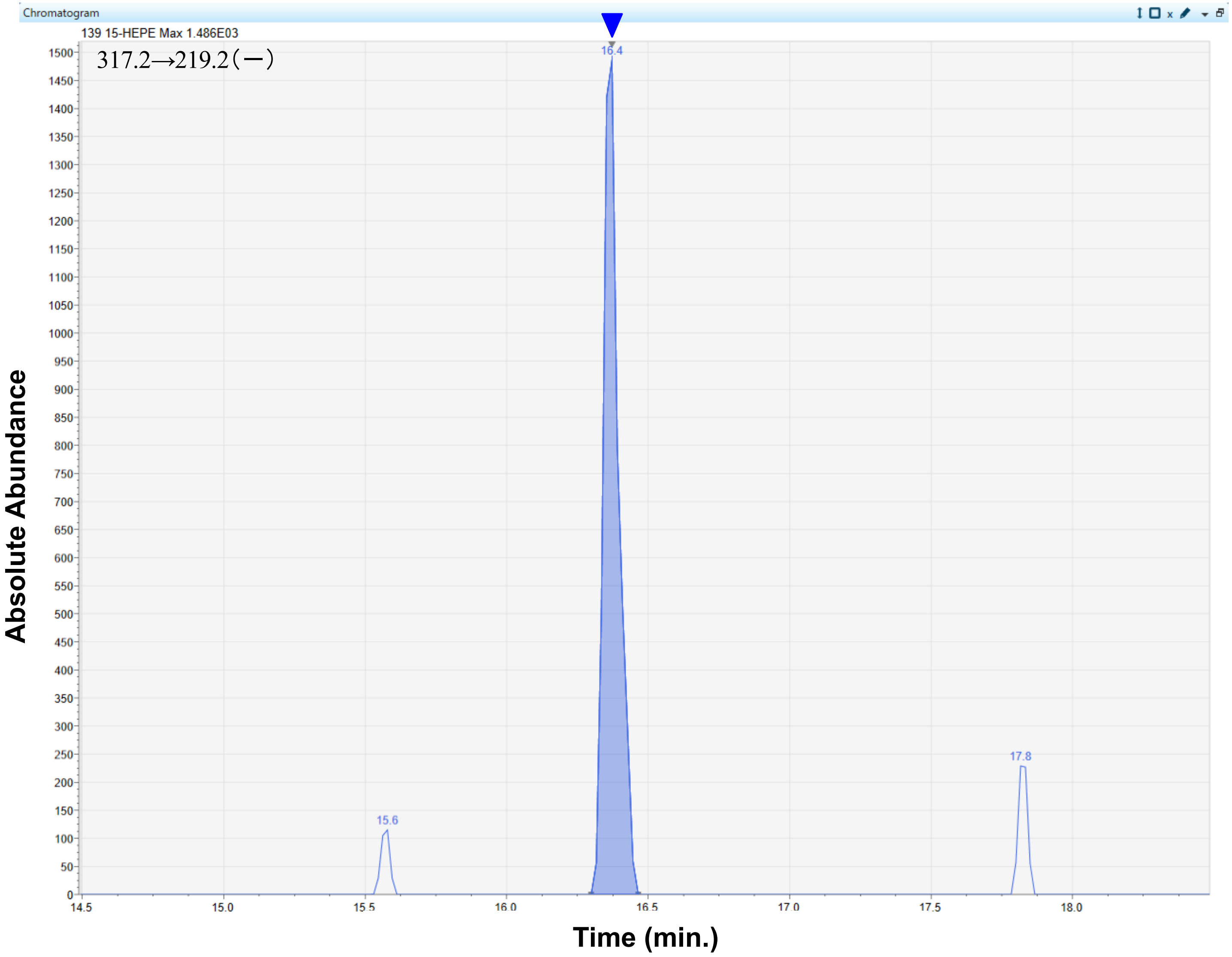

147 12-HEPE

Retention time: 16.5

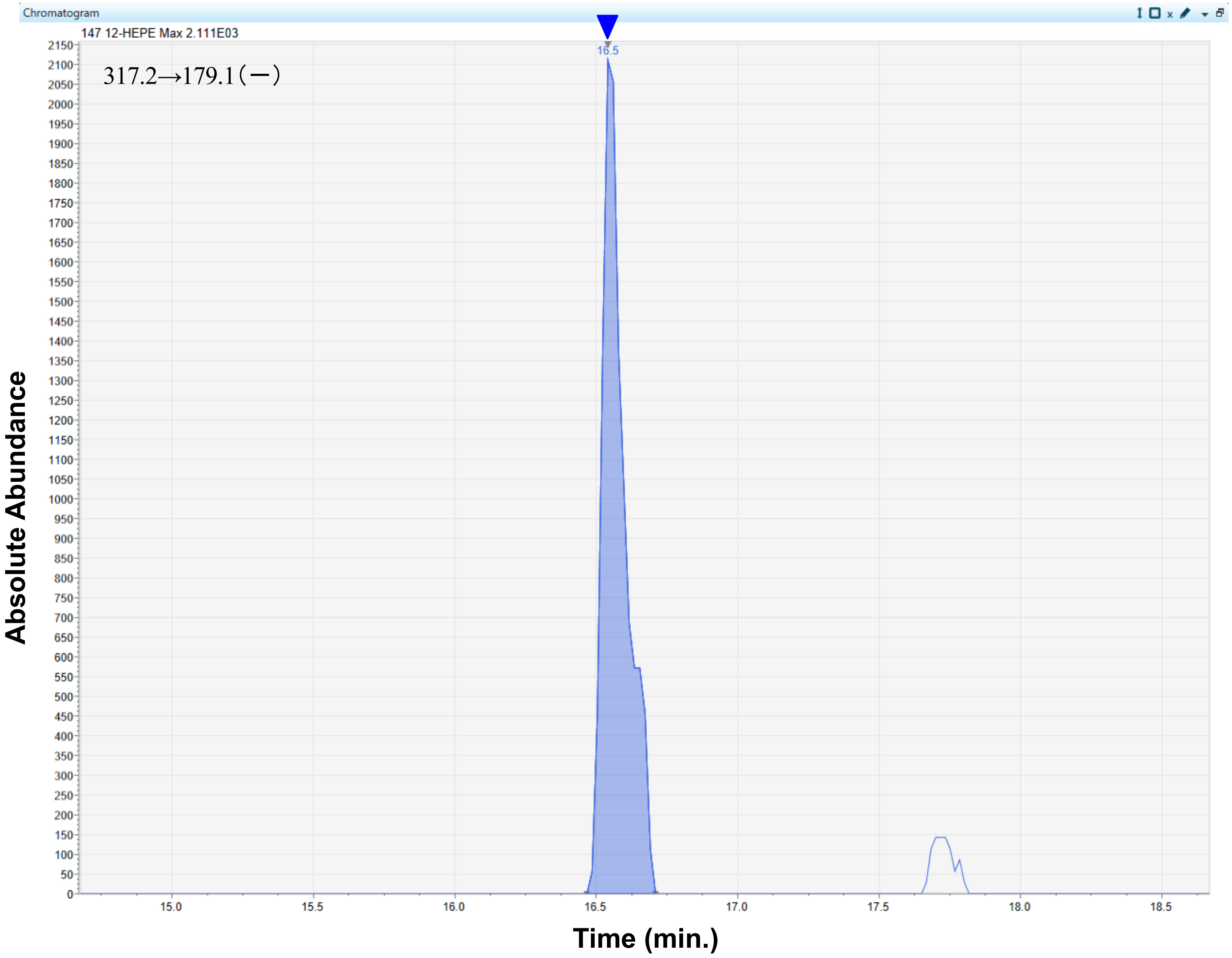

151 Lyso-PAF

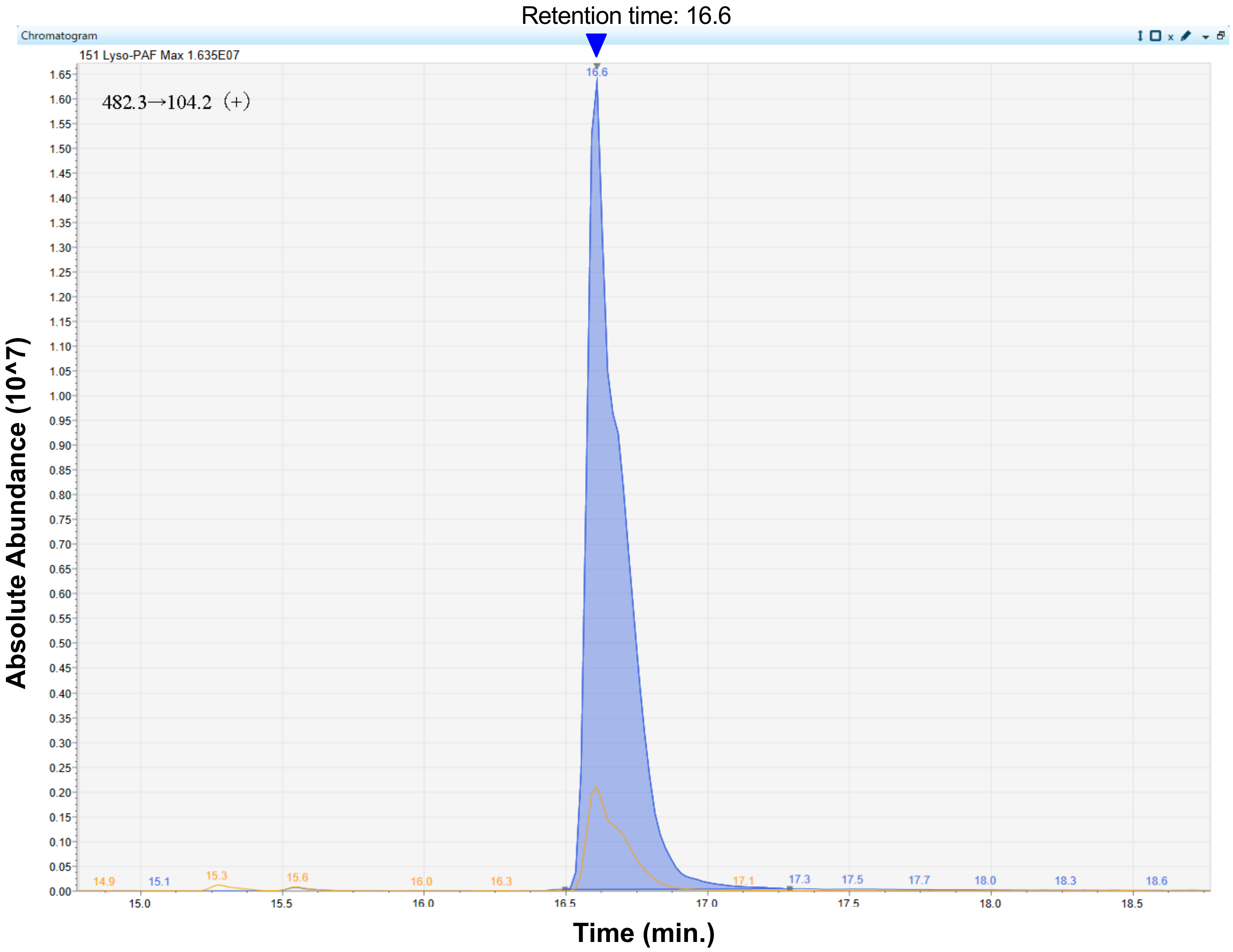
Retention time: 16.6
Chromatogram
151 Lyso-PAF Max 1.635E07
482.3→104.2 (+)
16.6
Absolute Abundance (10^7)
1.65
1.60
1.55
1.50
1.45
1.40
1.35
1.30
1.25
1.20
1.15
1.10
1.05
1.00
0.95
0.90
0.85
0.80
0.75
0.70
0.65
0.60
0.55
0.50
0.45
0.40
0.35
0.30
0.25
0.20
0.15
0.10
0.05
0.00
14.9
15.1
15.3
15.6
16.0
16.3
17.1
17.3
17.5
17.7
18.0
18.3
18.6
15.0
15.5
16.0
16.5
17.0
17.5
18.0
18.5
Time (min.)

153 13-HODE

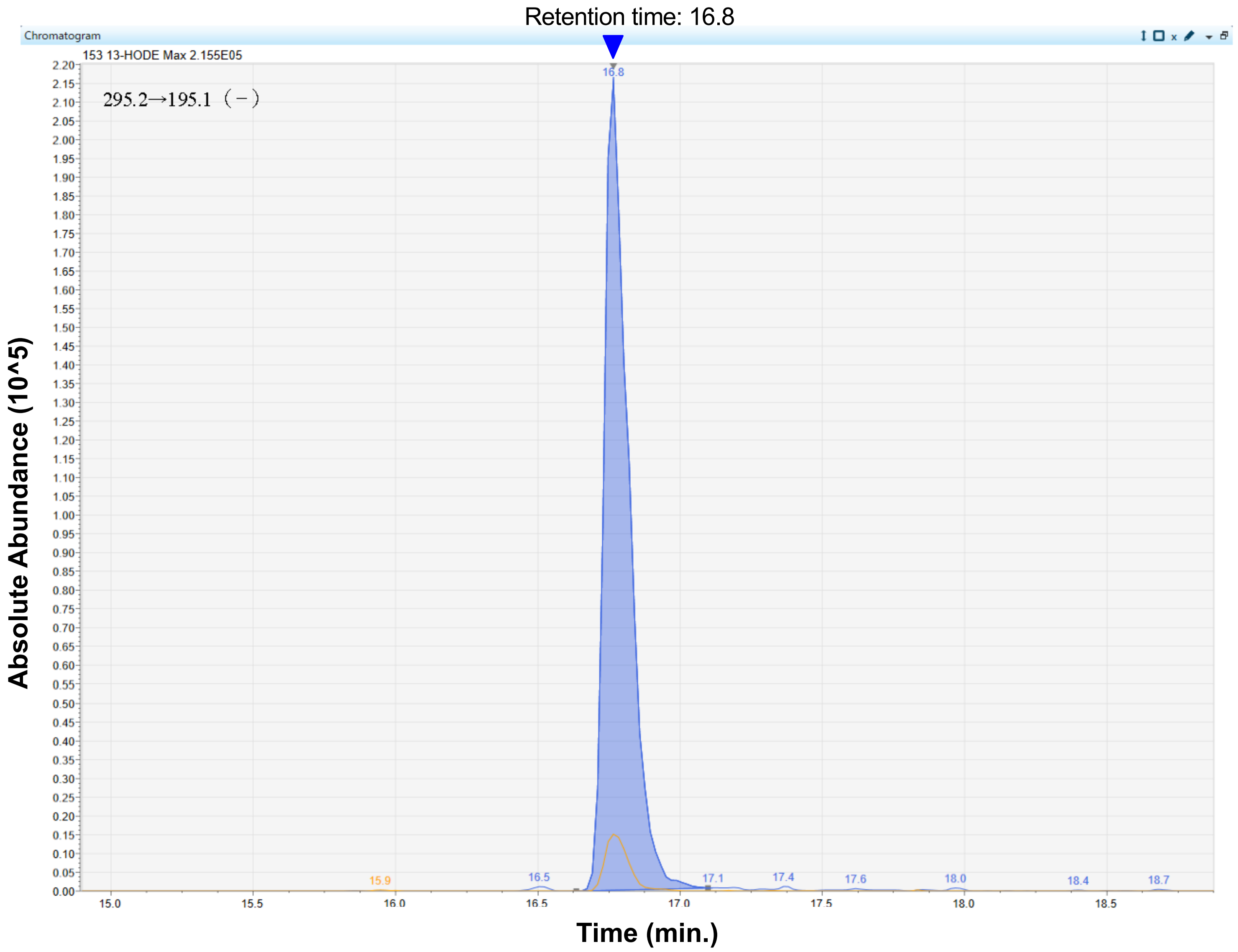
Retention time: 16.8
Chromatogram
153 13-HODE Max 2.155E05
295.2→195.1 (−)
16.8
15.9
16.5
17.1
17.4
17.6
18.0
18.4
18.7
Absolute Abundance (10^5)
Time (min.)

154 9-HODE

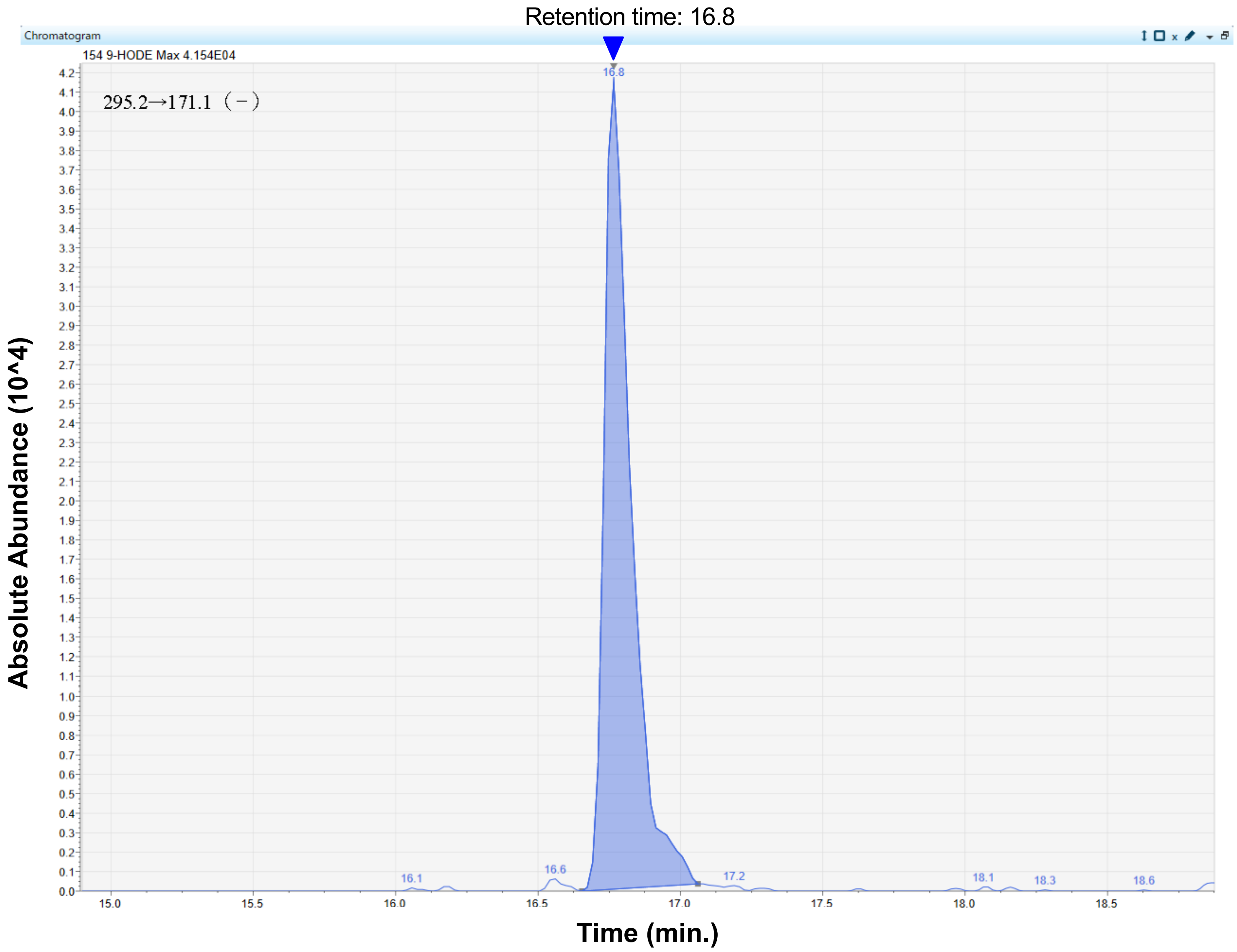
Retention time: 16.8
Chromatogram
154 9-HODE Max 4.154E04
295.2→171.1 (−)
16.8
16.1
16.6
17.2
18.1
18.3
18.6
Absolute Abundance (10^4)
Time (min.)

157 15-HETE-d_8 (IS)

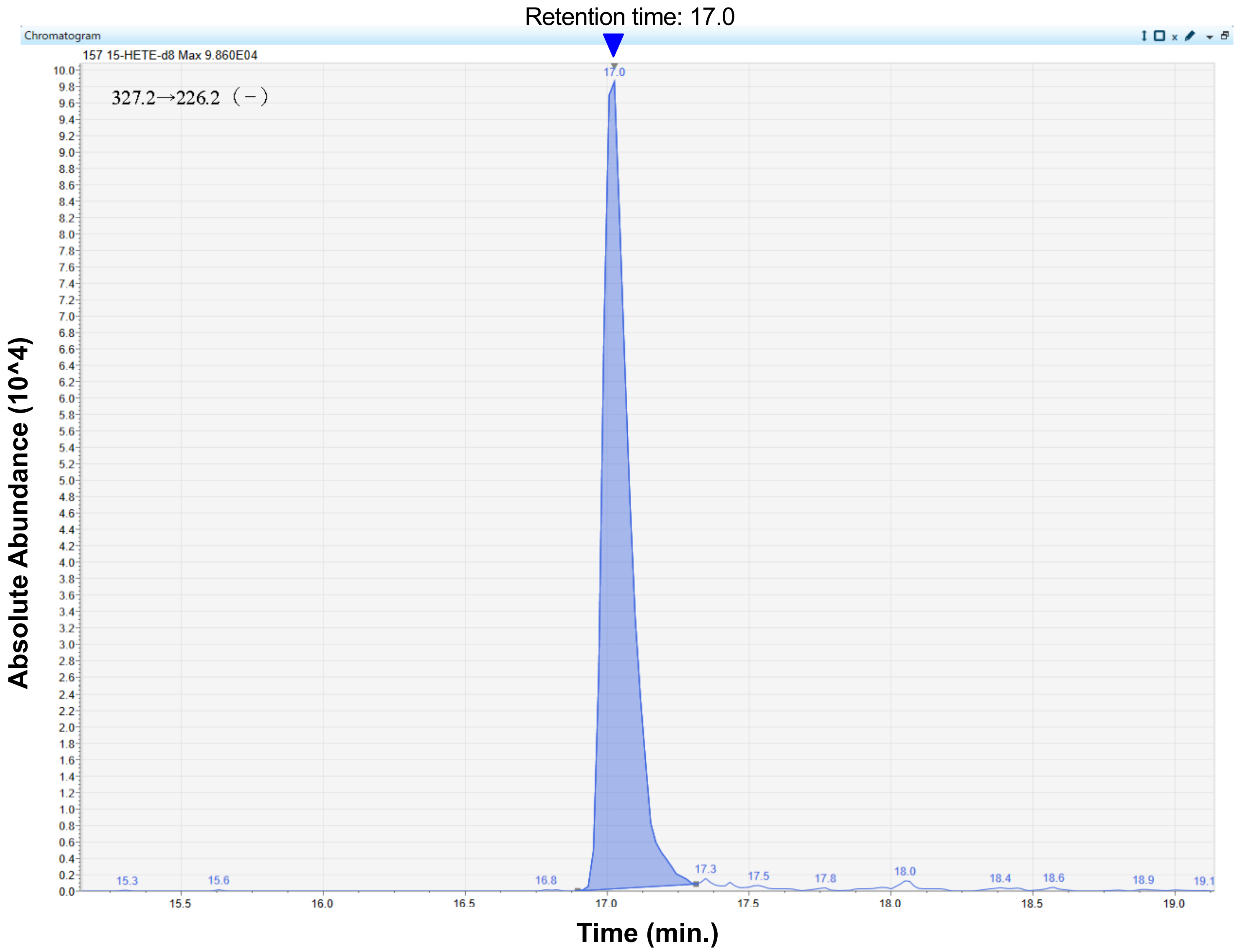

159 15-HETE

Retention time: 17.1

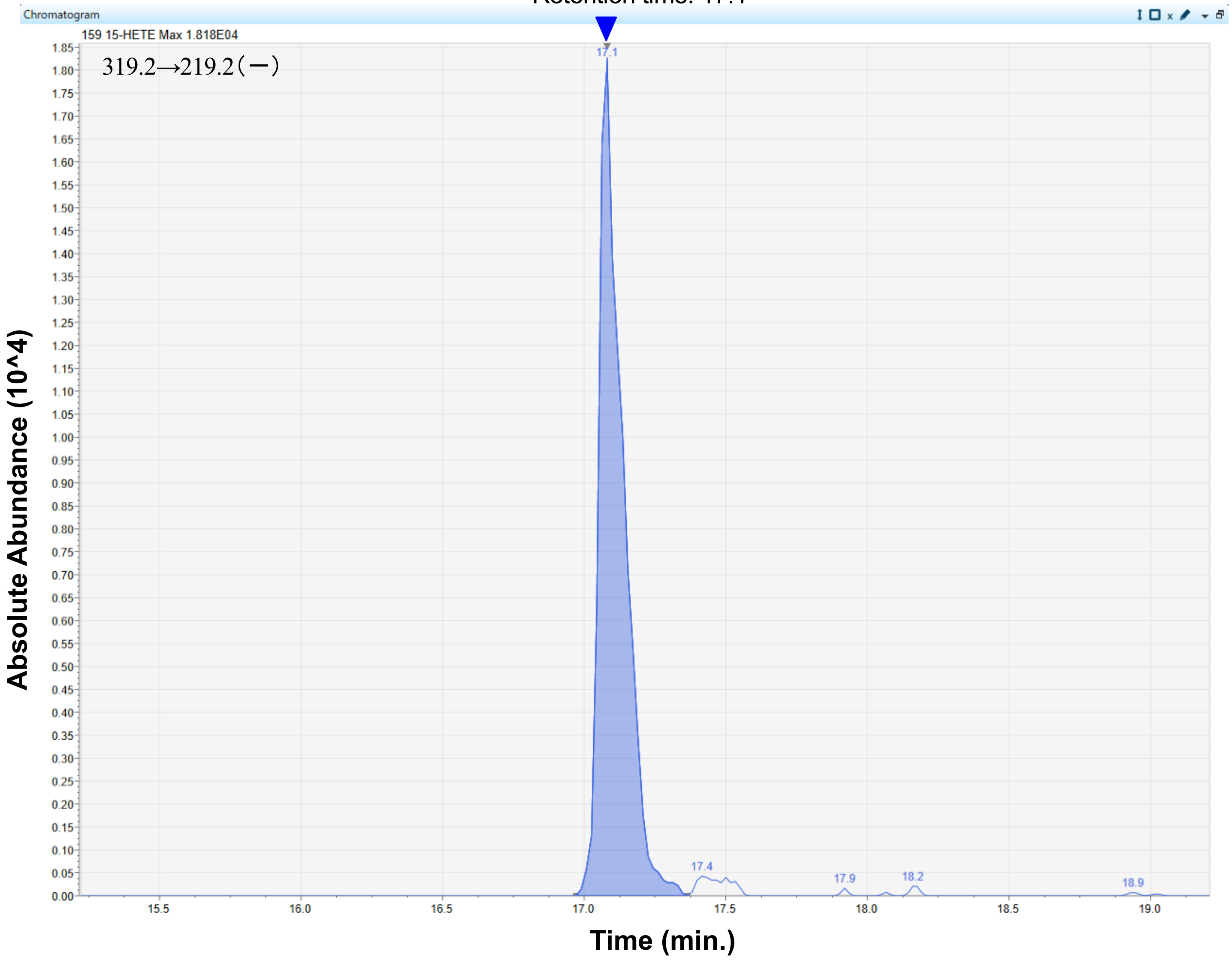

162 13-KODE

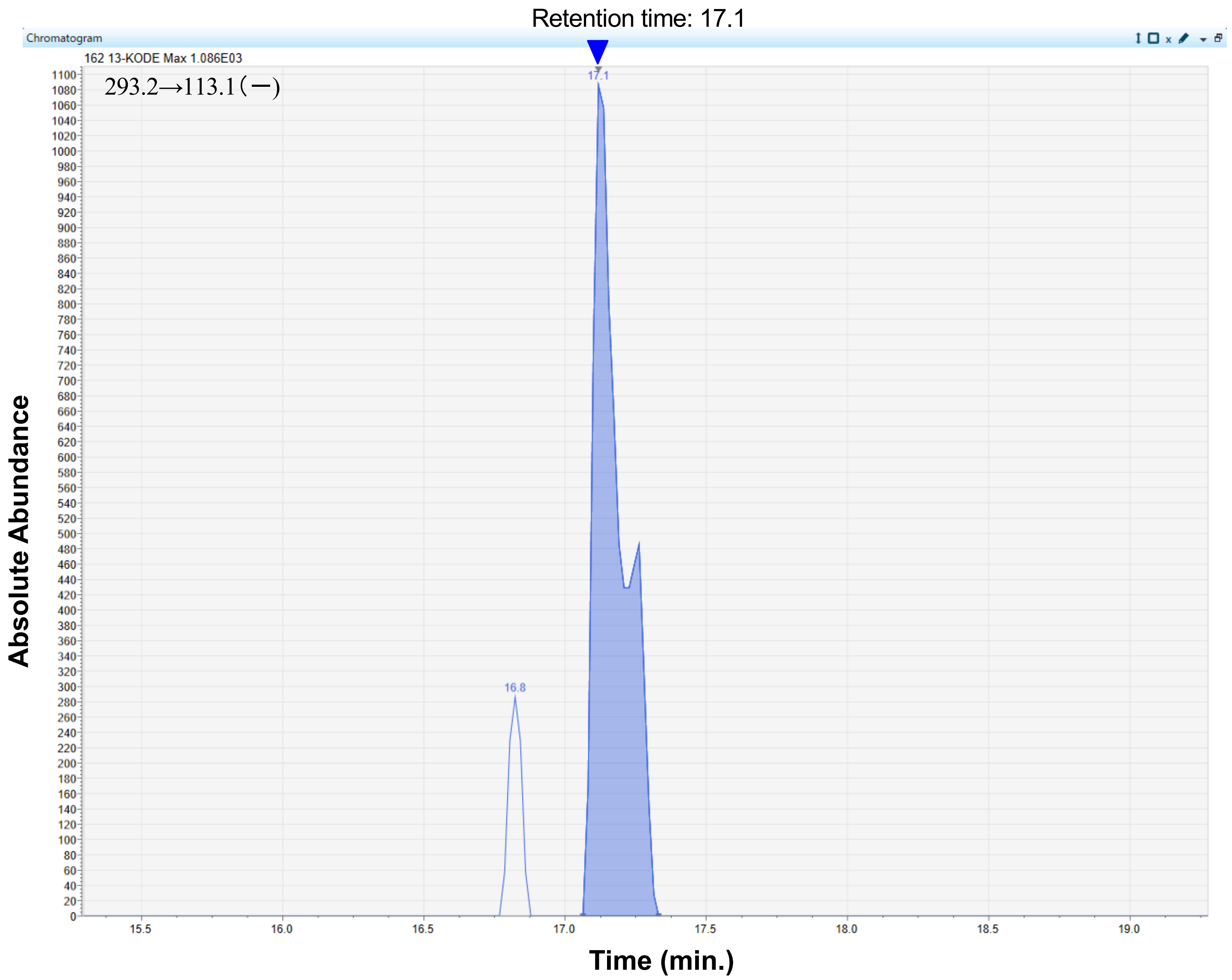
Retention time: 17.1
Chromatogram
162 13-KODE Max 1.086E03
293.2→113.1 (−)
17.1
16.8
Absolute Abundance
Time (min.)
15.5
16.0
16.5
17.0
17.5
18.0
18.5
19.0

164 16-HDHA

Retention time: 17.4

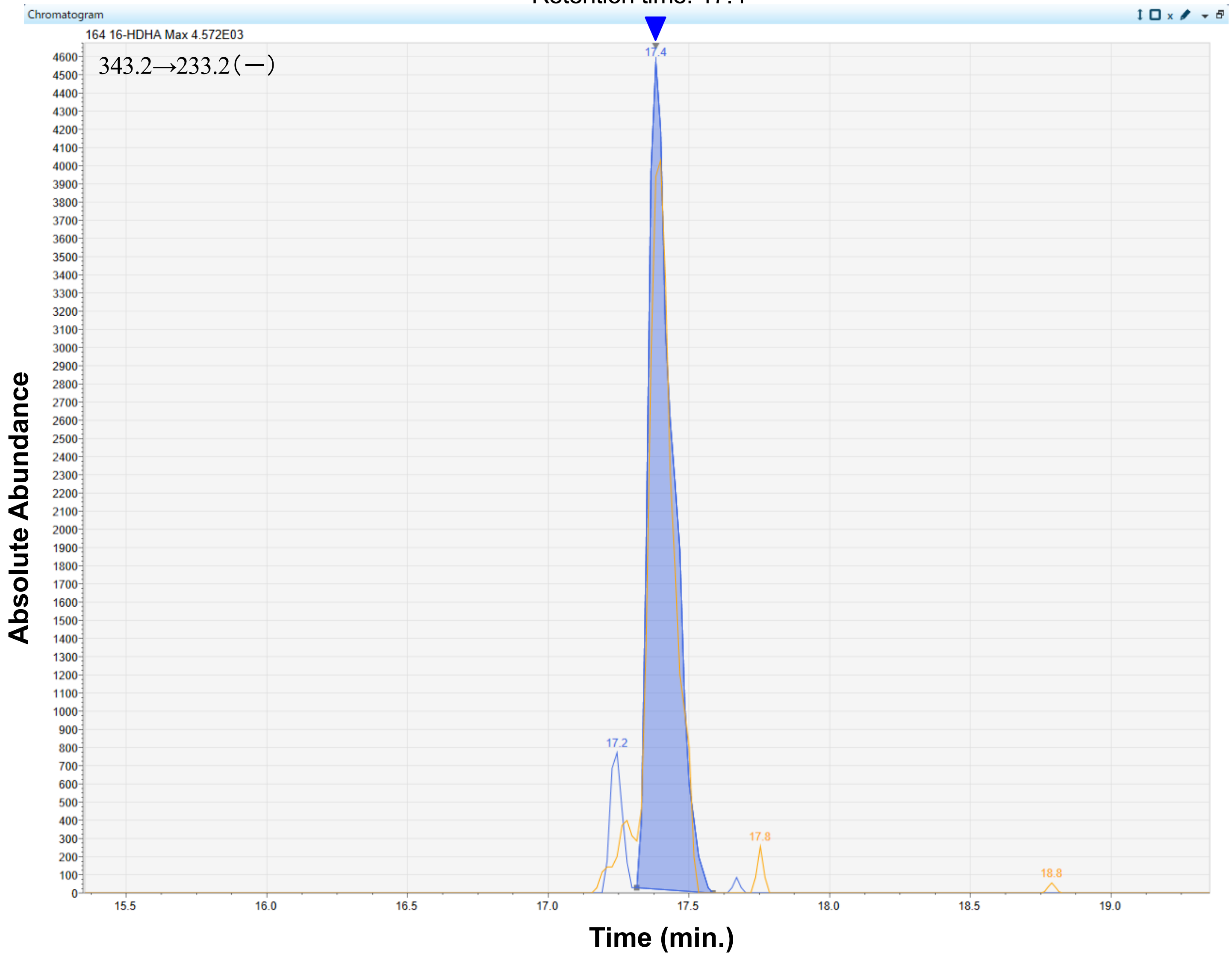

165 17-HDHA

Retention time: 17.3

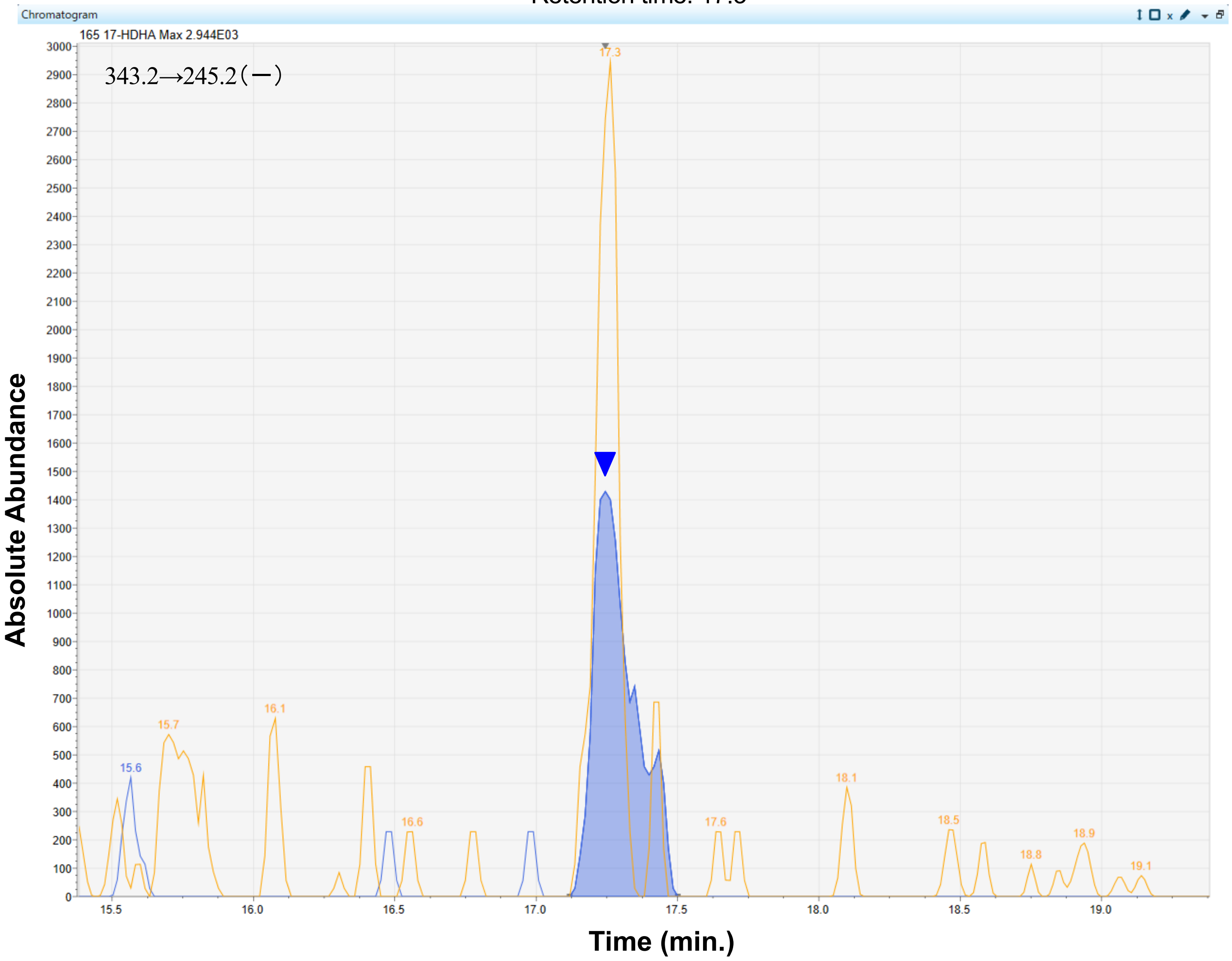

166 9-KODE

Retention time: 17.3

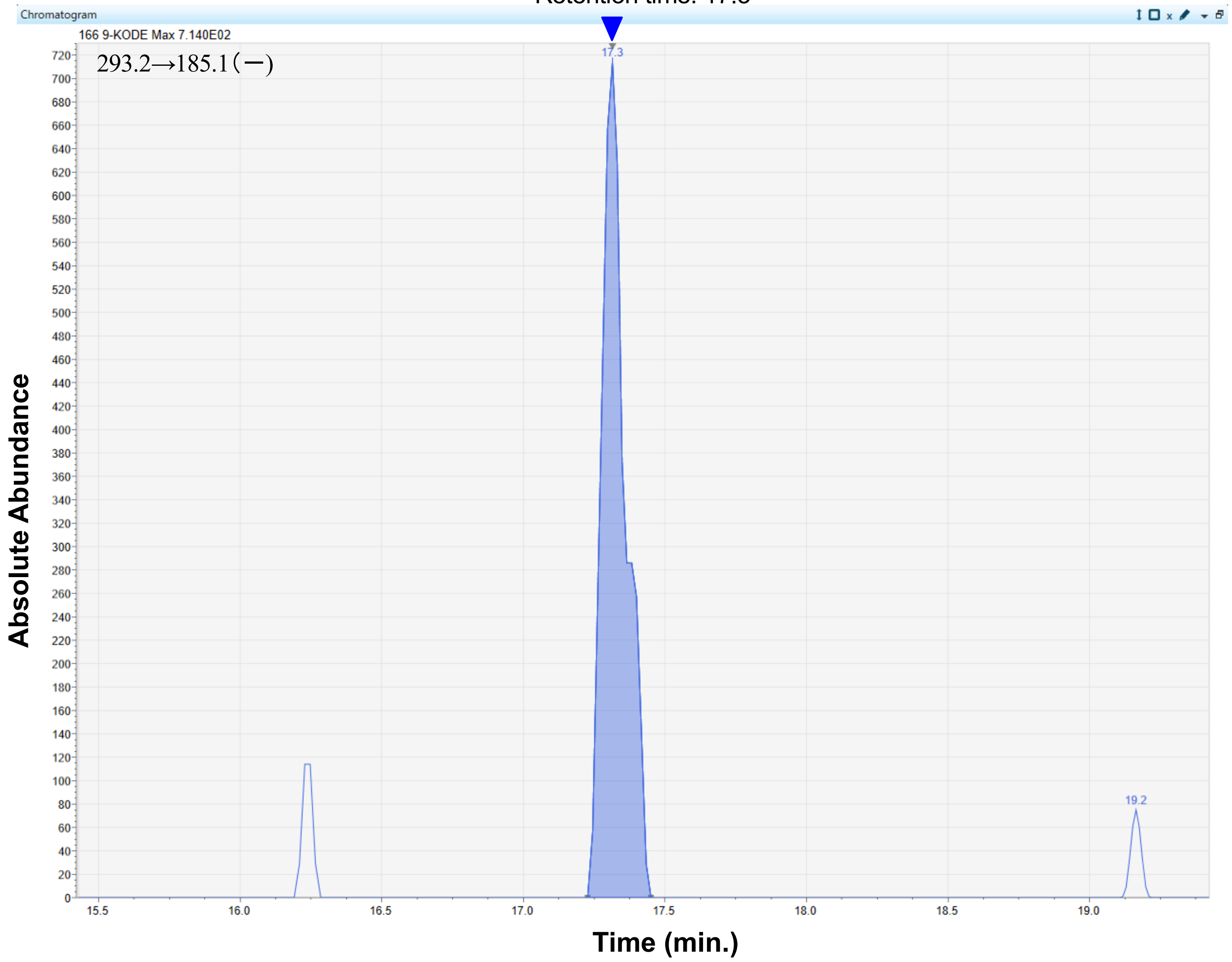

167 11-HETE

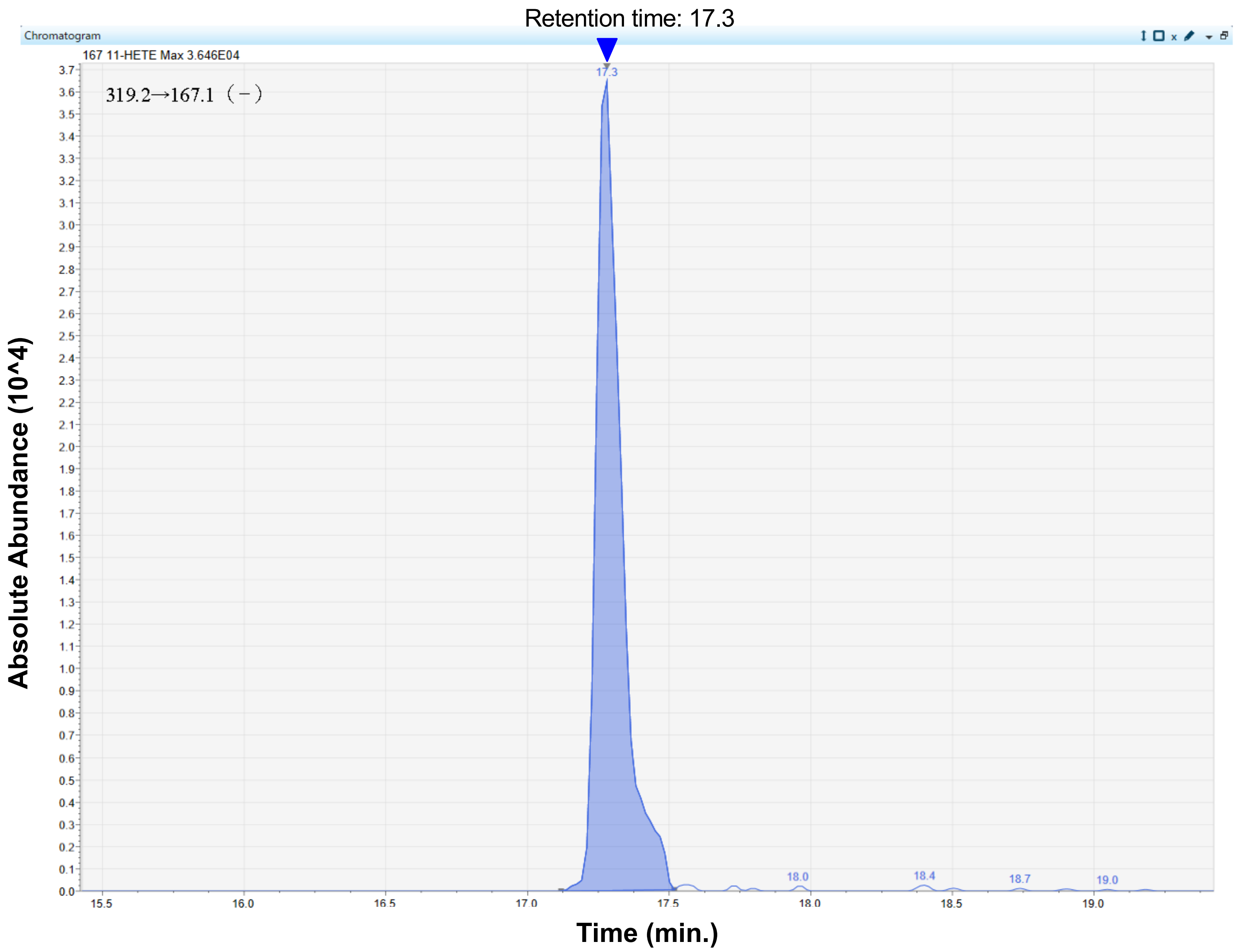
Retention time: 17.3
Chromatogram
167 11-HETE Max 3.646E04
319.2→167.1 (−)
17.3
18.0
18.4
18.7
19.0
Absolute Abundance (10^4)
Time (min.)

169 10-HDHA

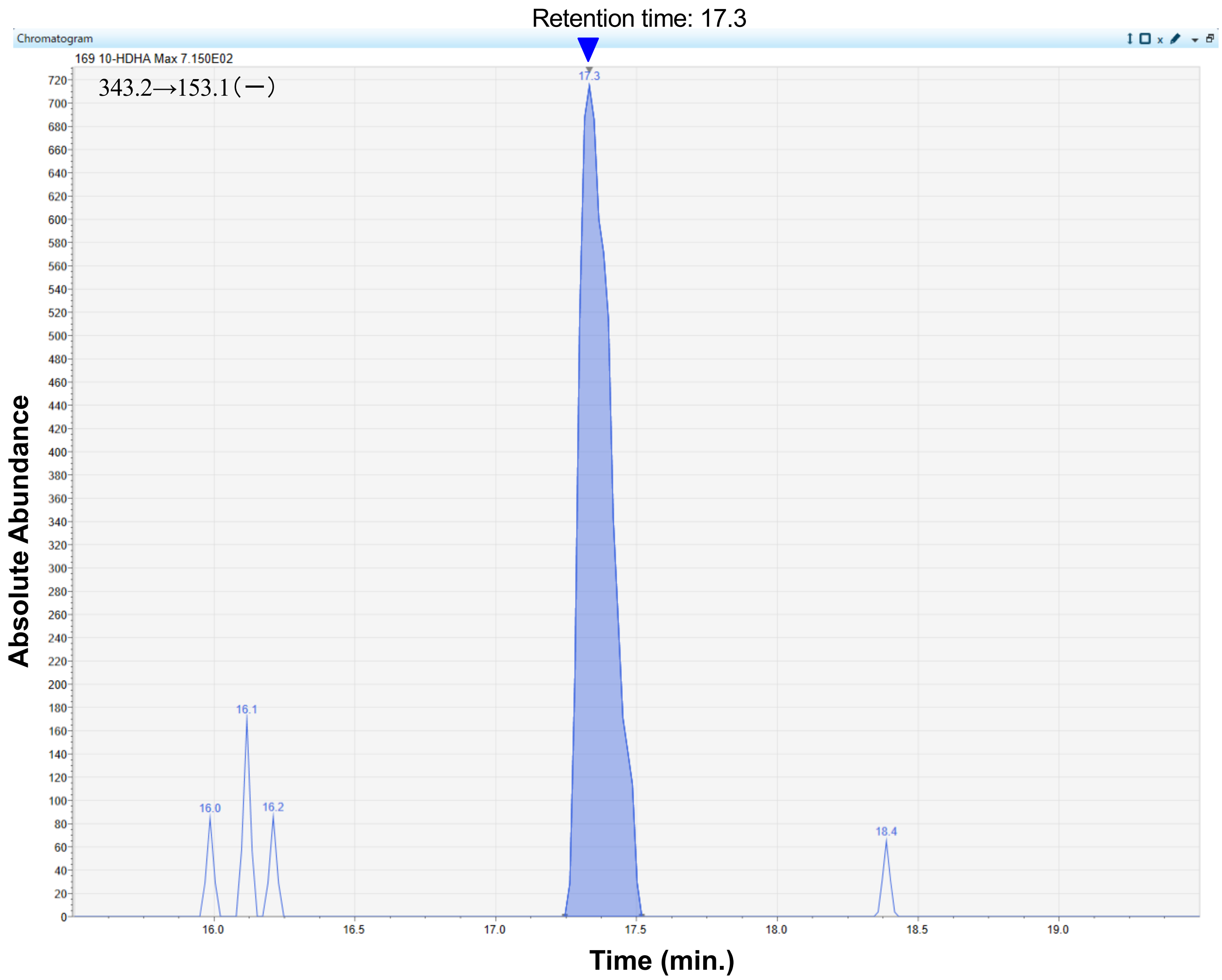
Retention time: 17.3
Chromatogram
169 10-HDHA Max 7.150E02
343.2→153.1 (−)
17.3
16.0
16.1
16.2
18.4
Absolute Abundance
Time (min.)

170 8-HETE

Retention time: 17.4

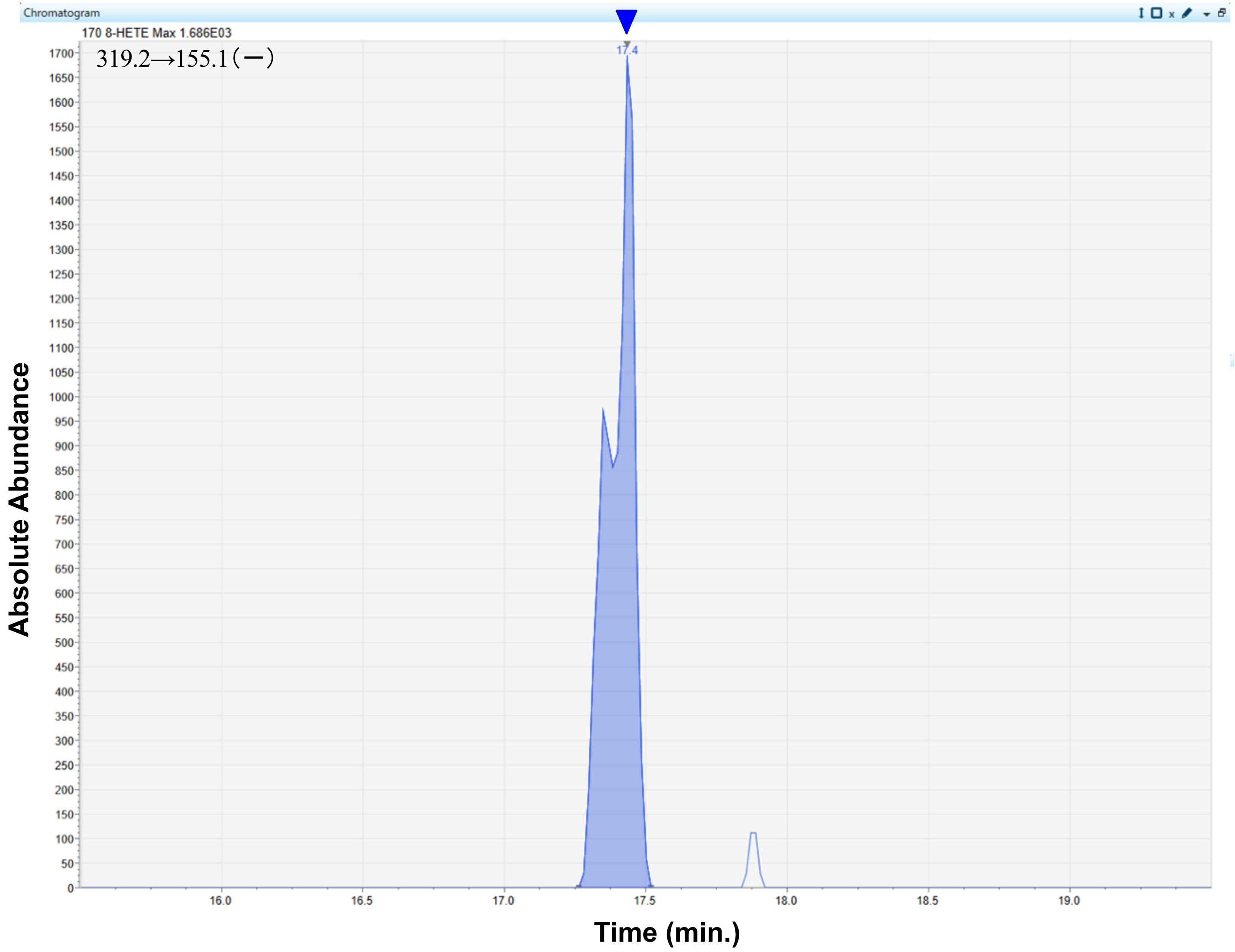

171 12-HETE-d_8 (IS)

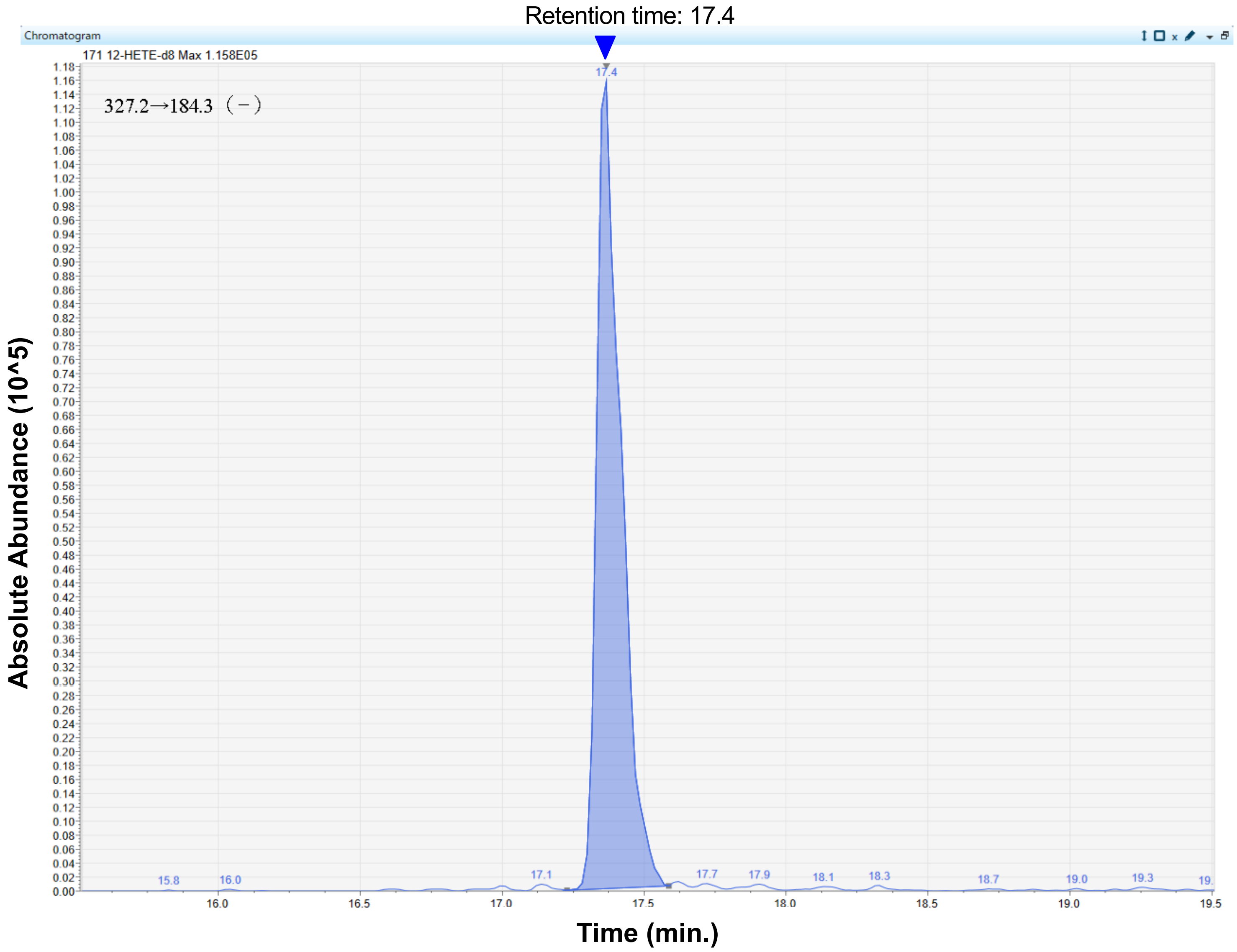

172 14-HDHA

Retention time: 17.4

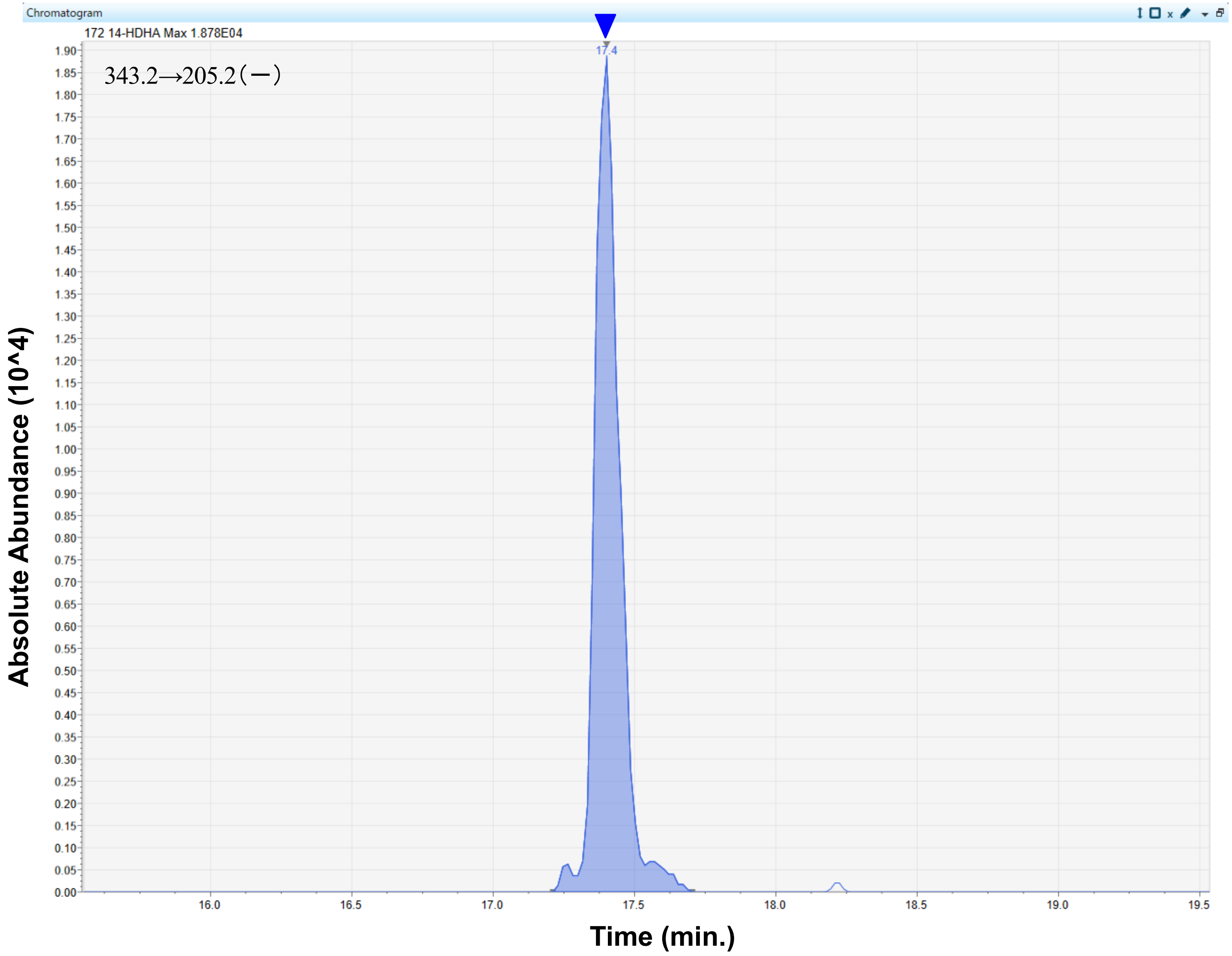

176 12-HETE

Retention time: 17.4

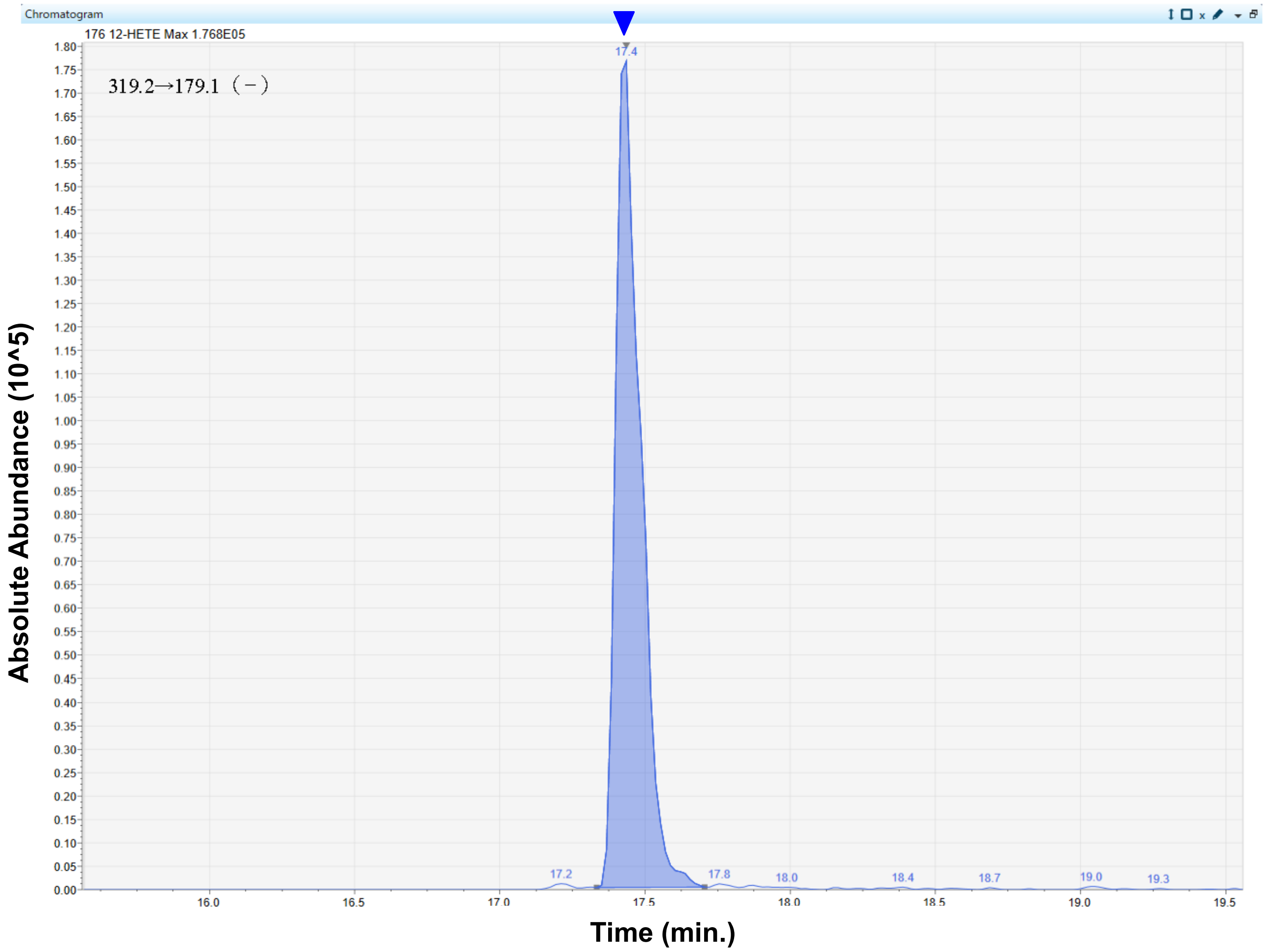

180 5-HETE-d_8 (IS)

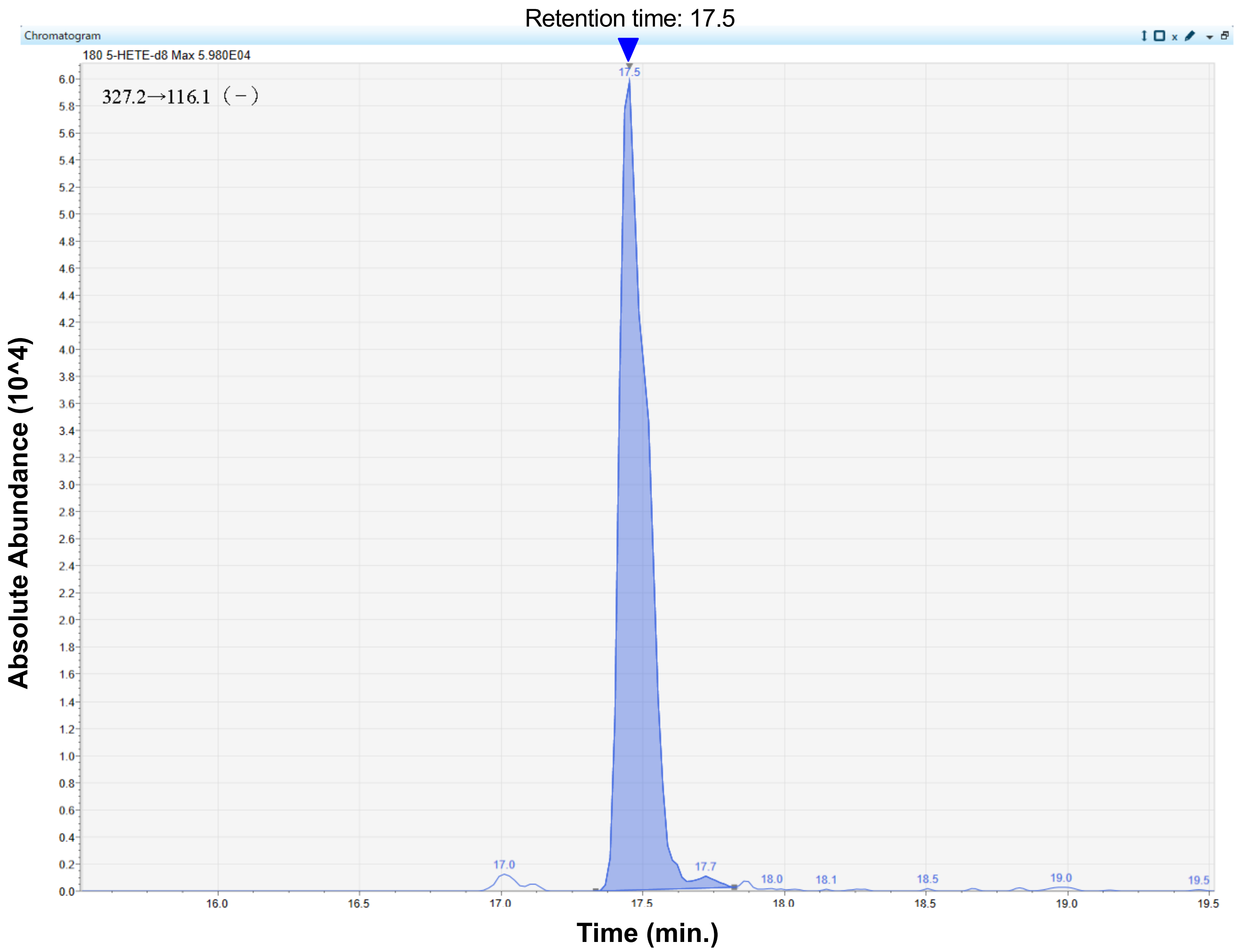

181 8-HDHA

Retention time: 17.4

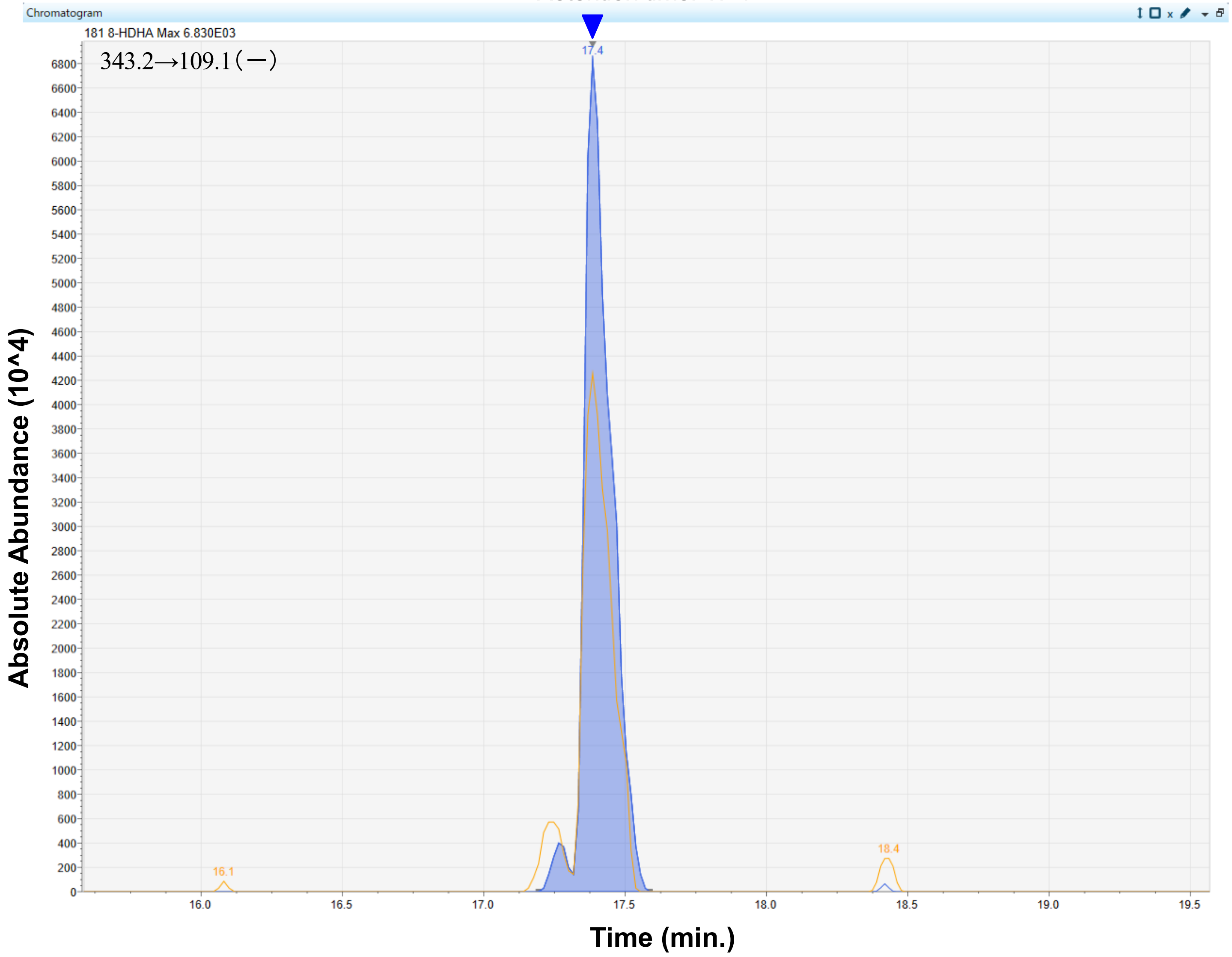

182 5-HETE

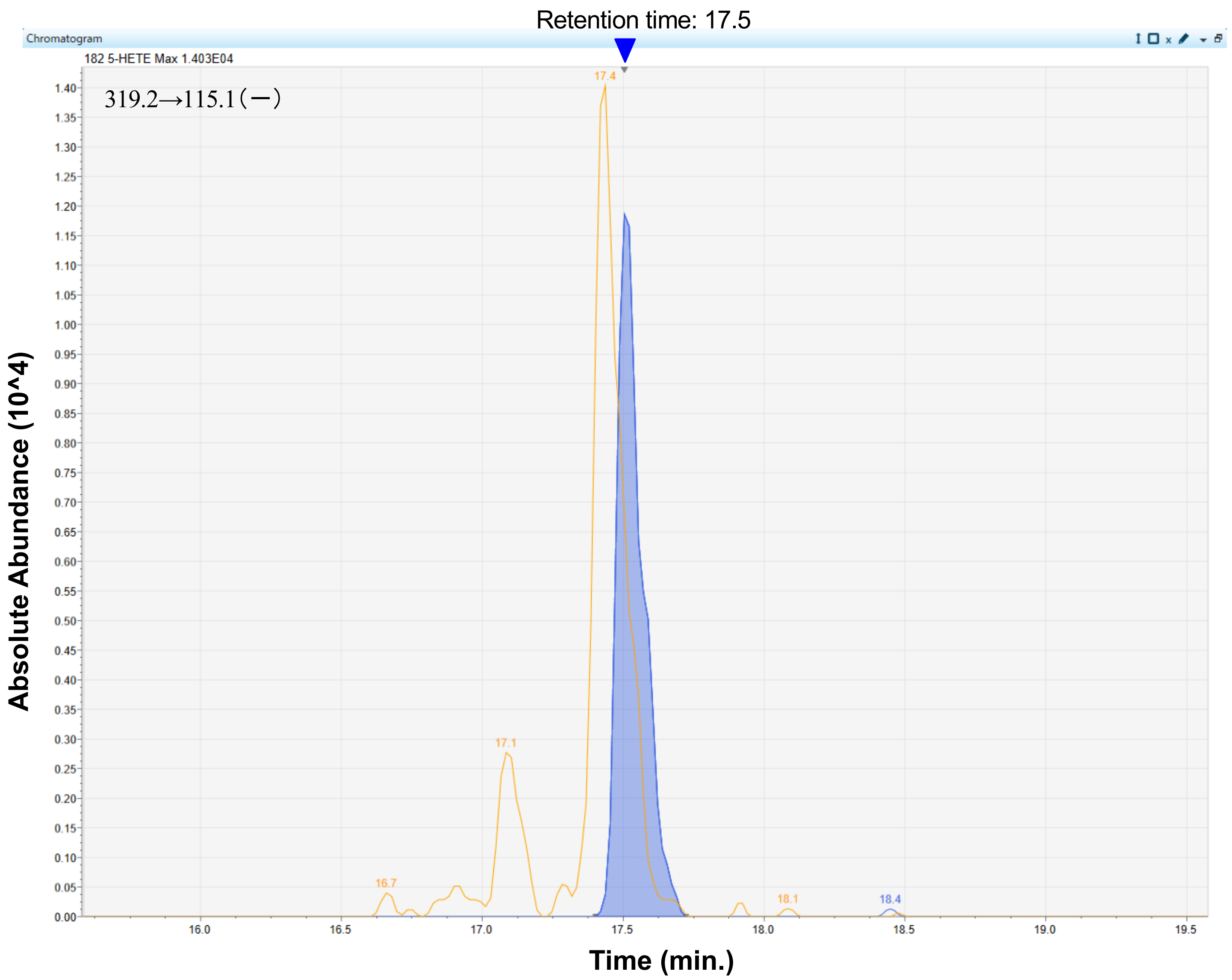
Retention time: 17.5
Chromatogram
182 5-HETE Max 1.403E04
319.2→115.1 (−)
17.4
17.1
16.7
18.1
18.4
Absolute Abundance (10^4)
1.40
1.35
1.30
1.25
1.20
1.15
1.10
1.05
1.00
0.95
0.90
0.85
0.80
0.75
0.70
0.65
0.60
0.55
0.50
0.45
0.40
0.35
0.30
0.25
0.20
0.15
0.10
0.05
0.00
16.0
16.5
17.0
17.5
18.0
18.5
19.0
19.5
Time (min.)

184 15-HETrE

Retention time: 17.6

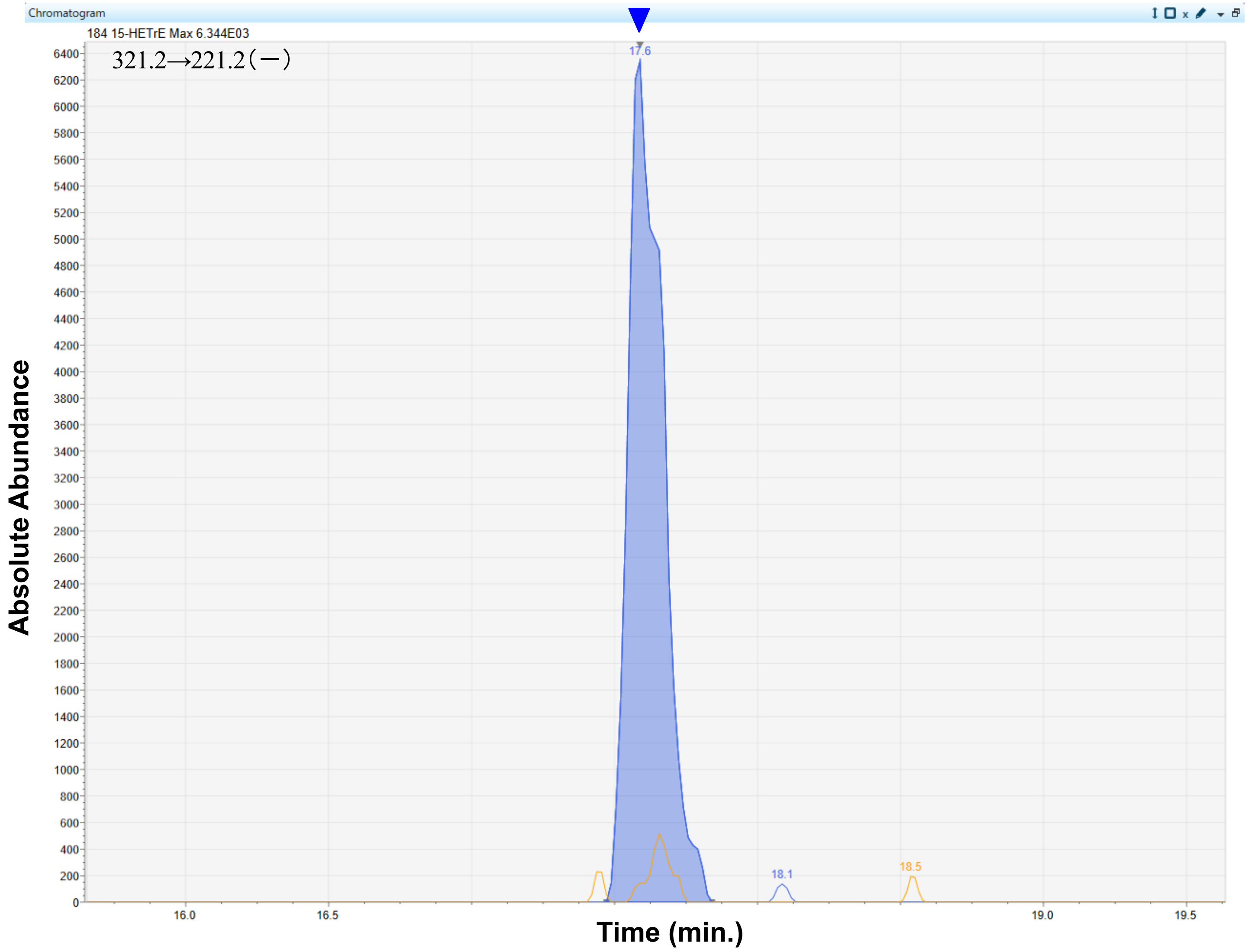

185 PAF-d_4 (IS)

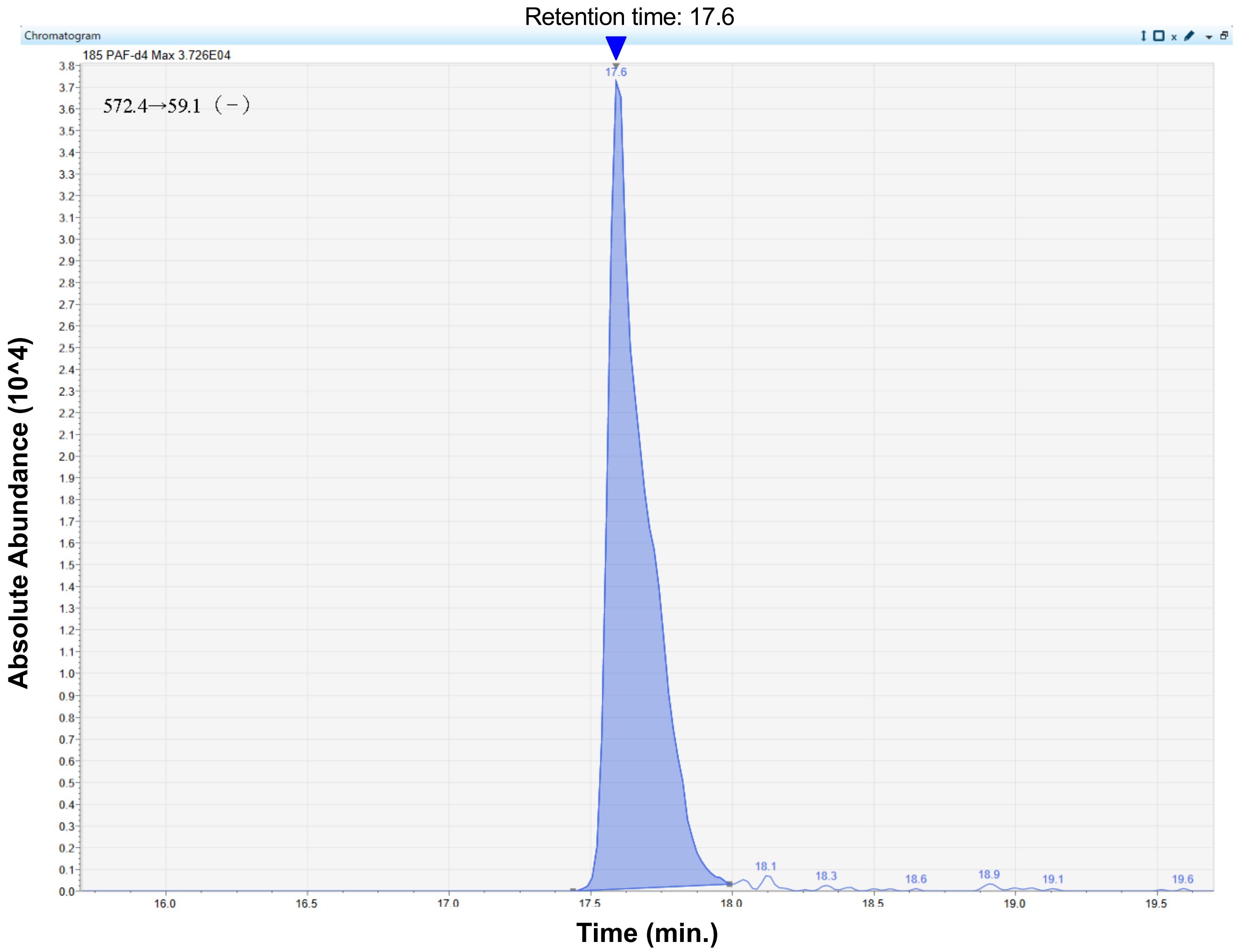
Retention time: 17.6
Chromatogram
185 PAF-d4 Max 3.726E04
572.4→59.1 (−)
17.6
18.1
18.3
18.6
18.9
19.1
19.6
Absolute Abundance (10^4)
Time (min.)

186 PAF

Retention time: 17.6

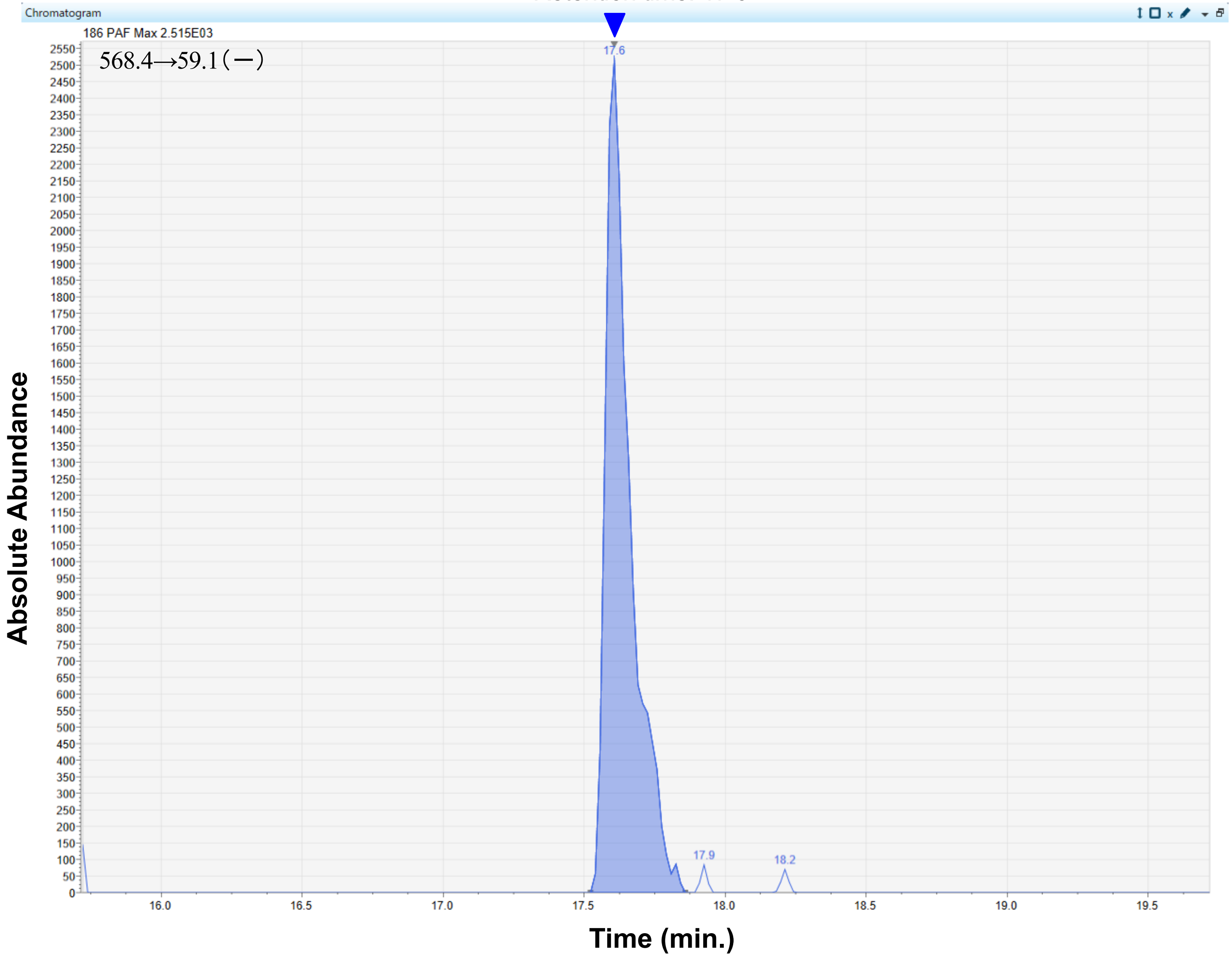

187 8-HETrE

Retention time: 17.7

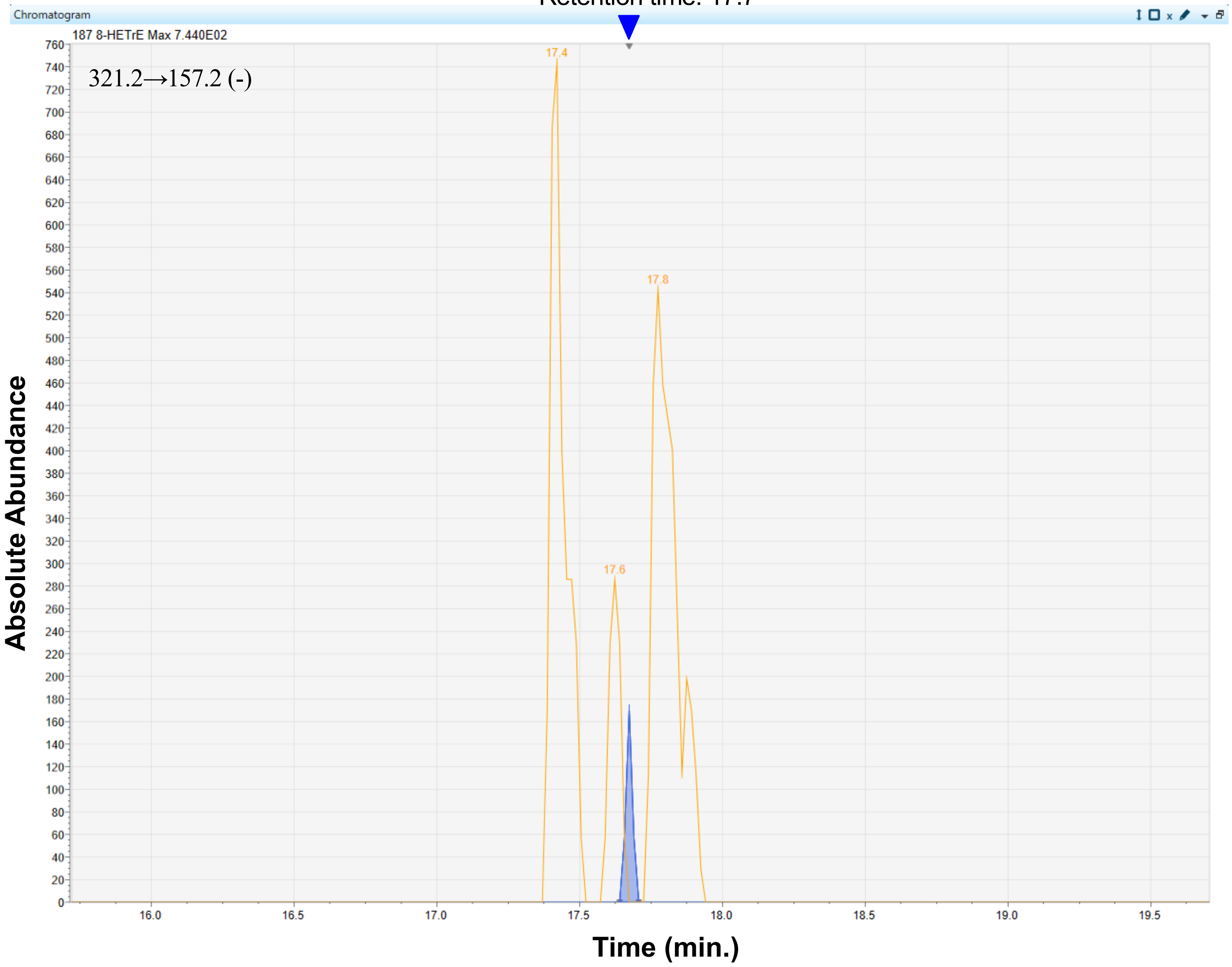

189 12-KETE

Retention time: 17.7

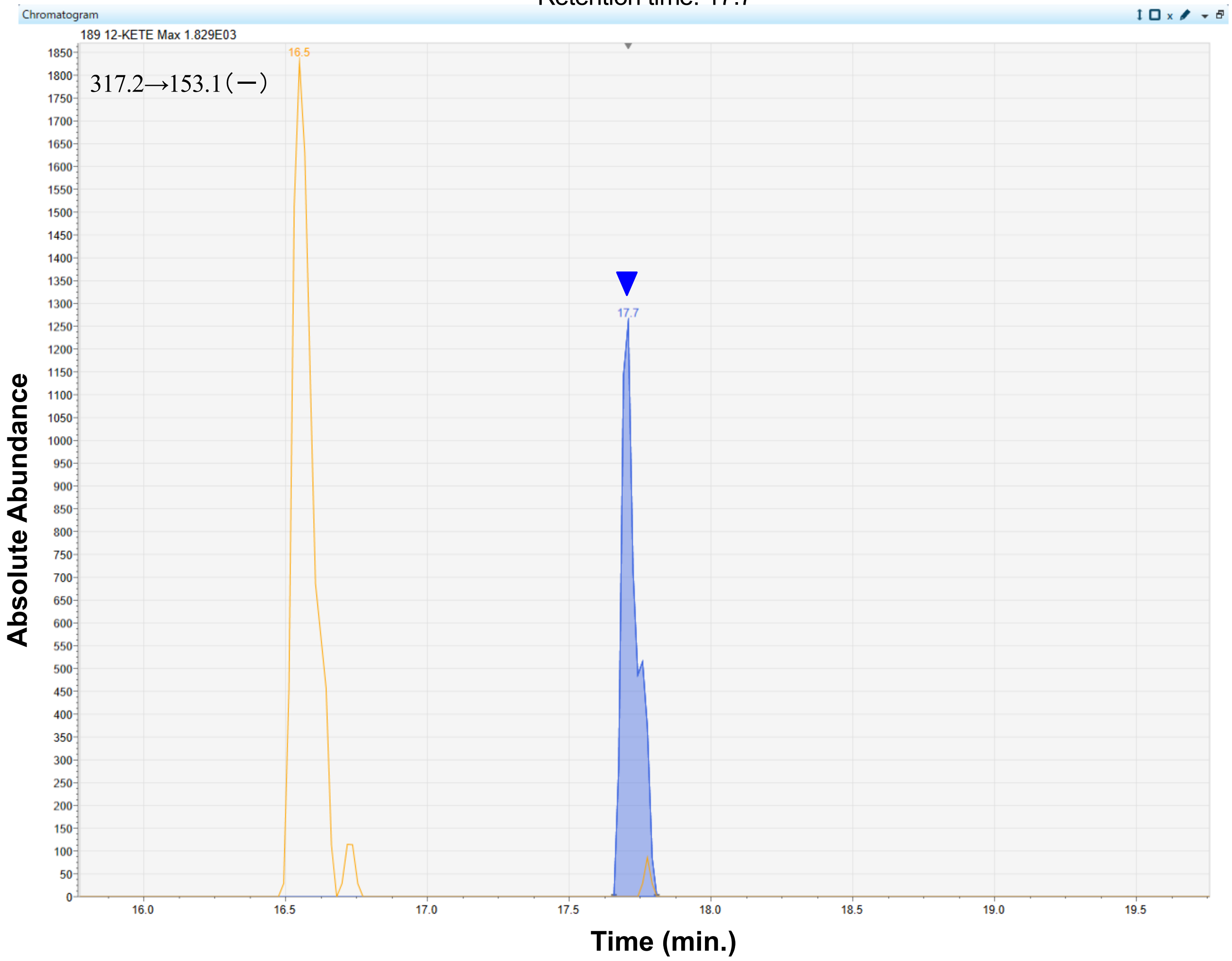

197 5-KETE

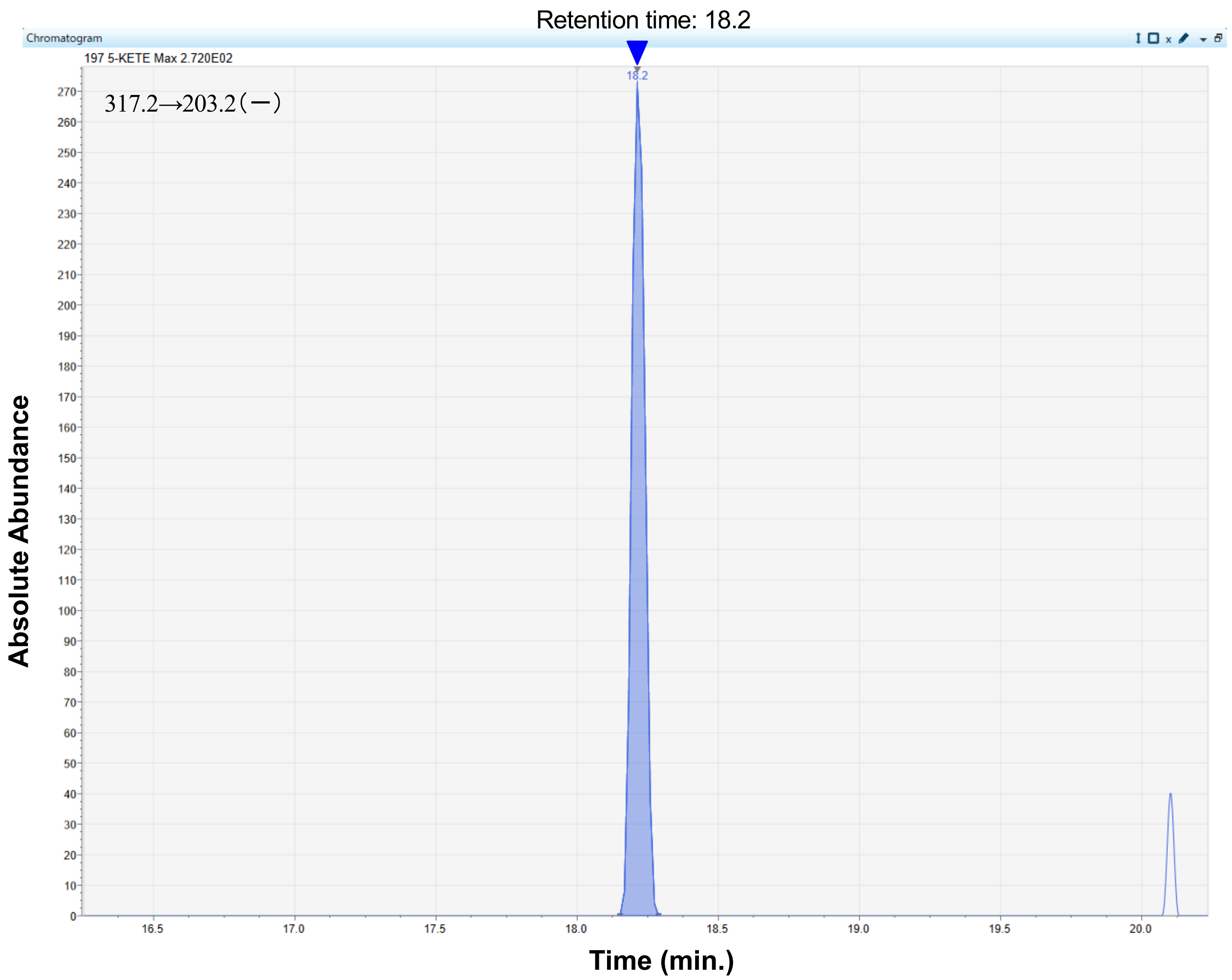
Retention time: 18.2
Chromatogram
197 5-KETE Max 2.720E02
18.2
317.2→203.2（－）
270
260
250
240
230
220
210
200
190
180
170
160
150
140
130
120
110
100
90
80
70
60
50
40
30
20
10
0
16.5
17.0
17.5
18.0
18.5
19.0
19.5
20.0
Absolute Abundance
Time (min.)

199 11-HEDE

Retention time: 18.3

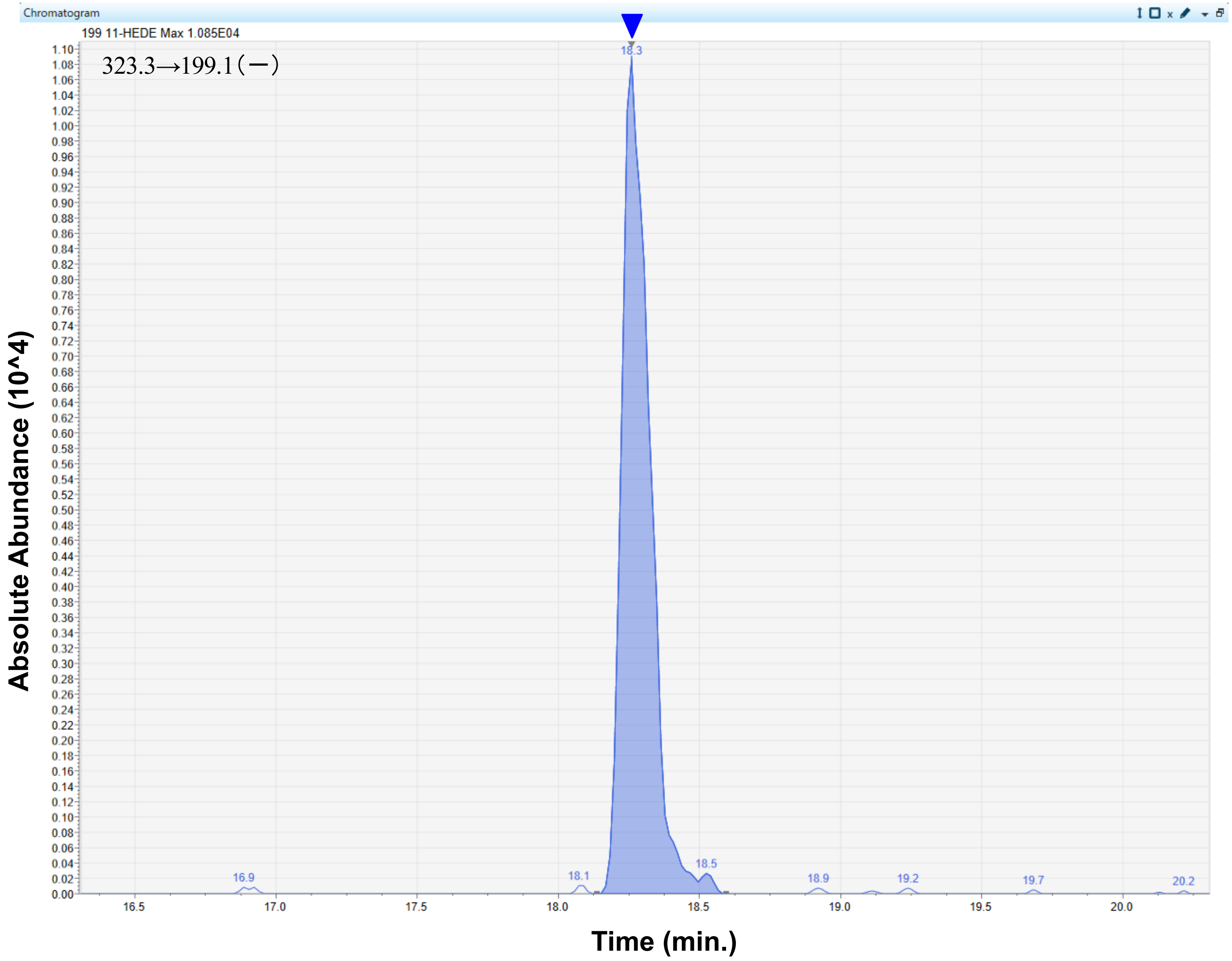

201 15-HEDE

Retention time: 18.3

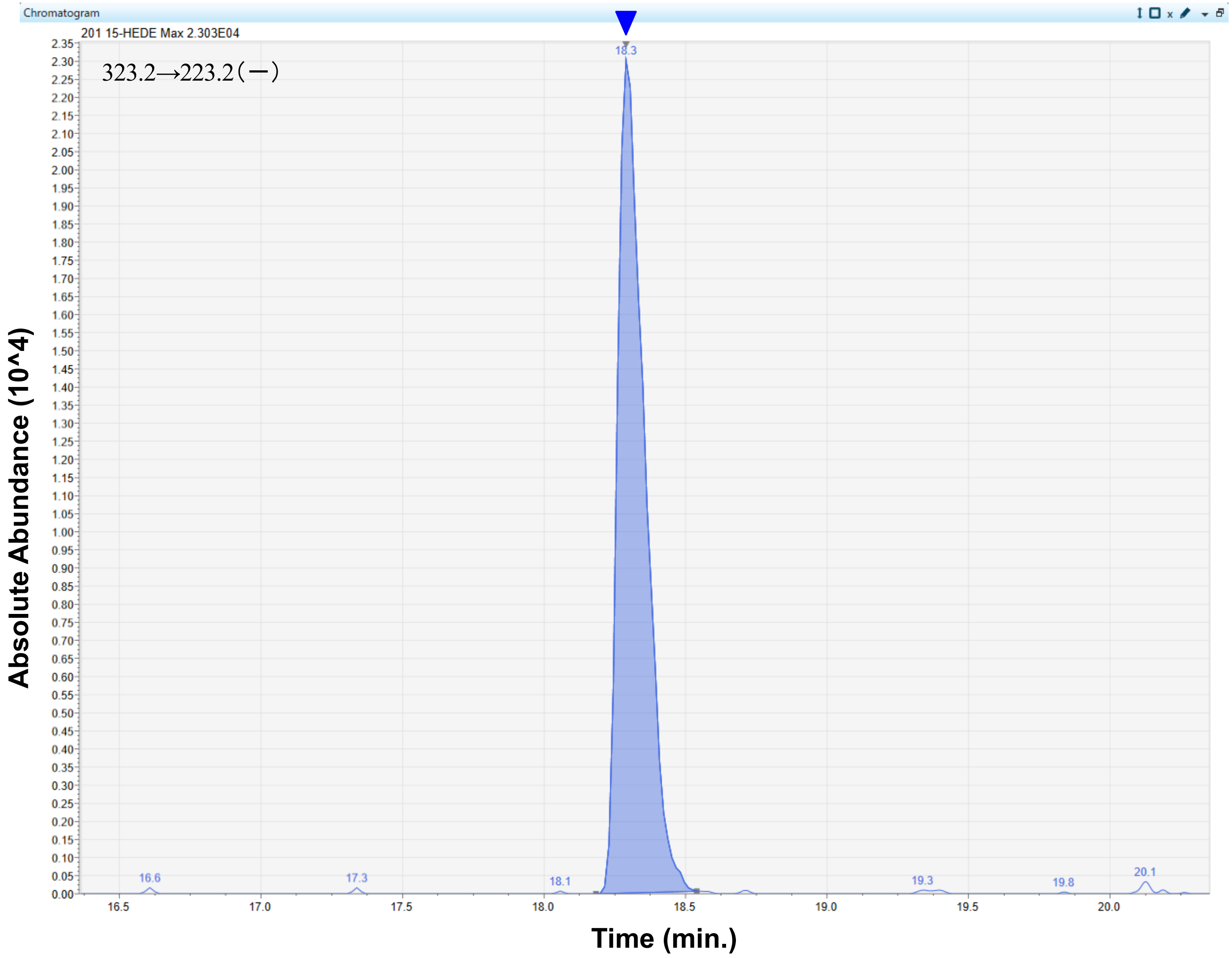

202 11,12-EET-d_{11} (IS)

Retention time: 18.3

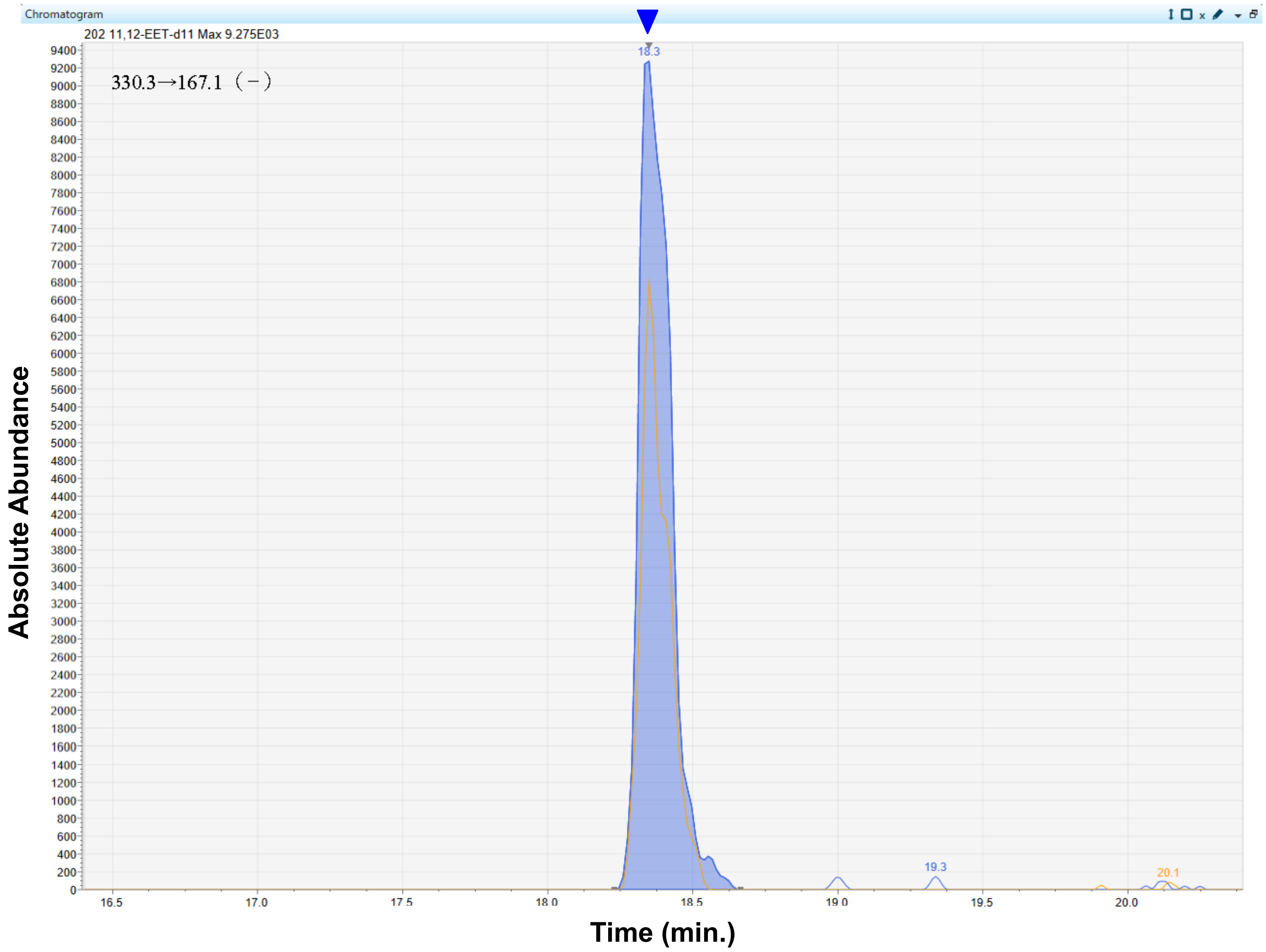

203 AEA

Retention time: 18.4

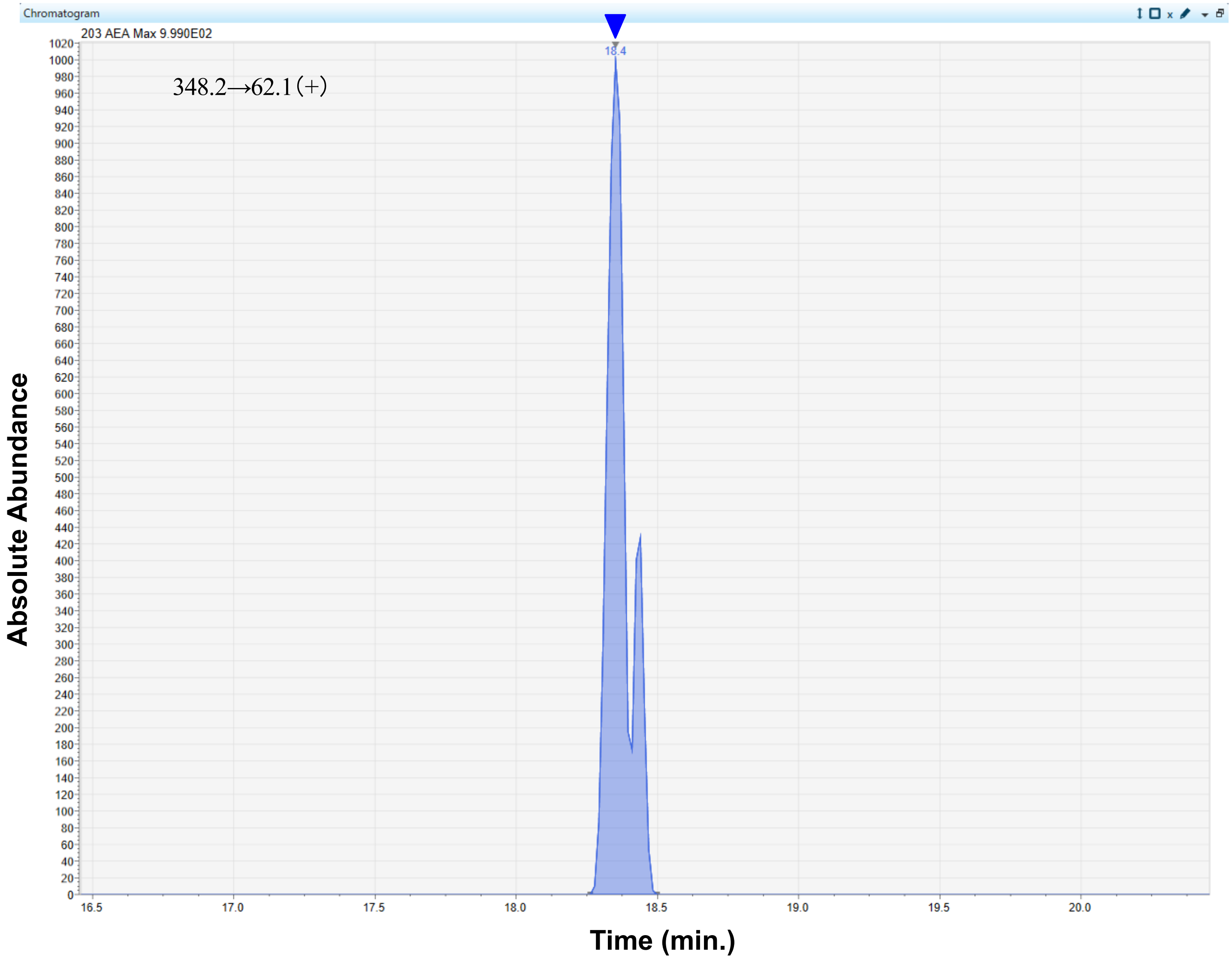

209 OEA-d_4 (IS)

Retention time: 19.2

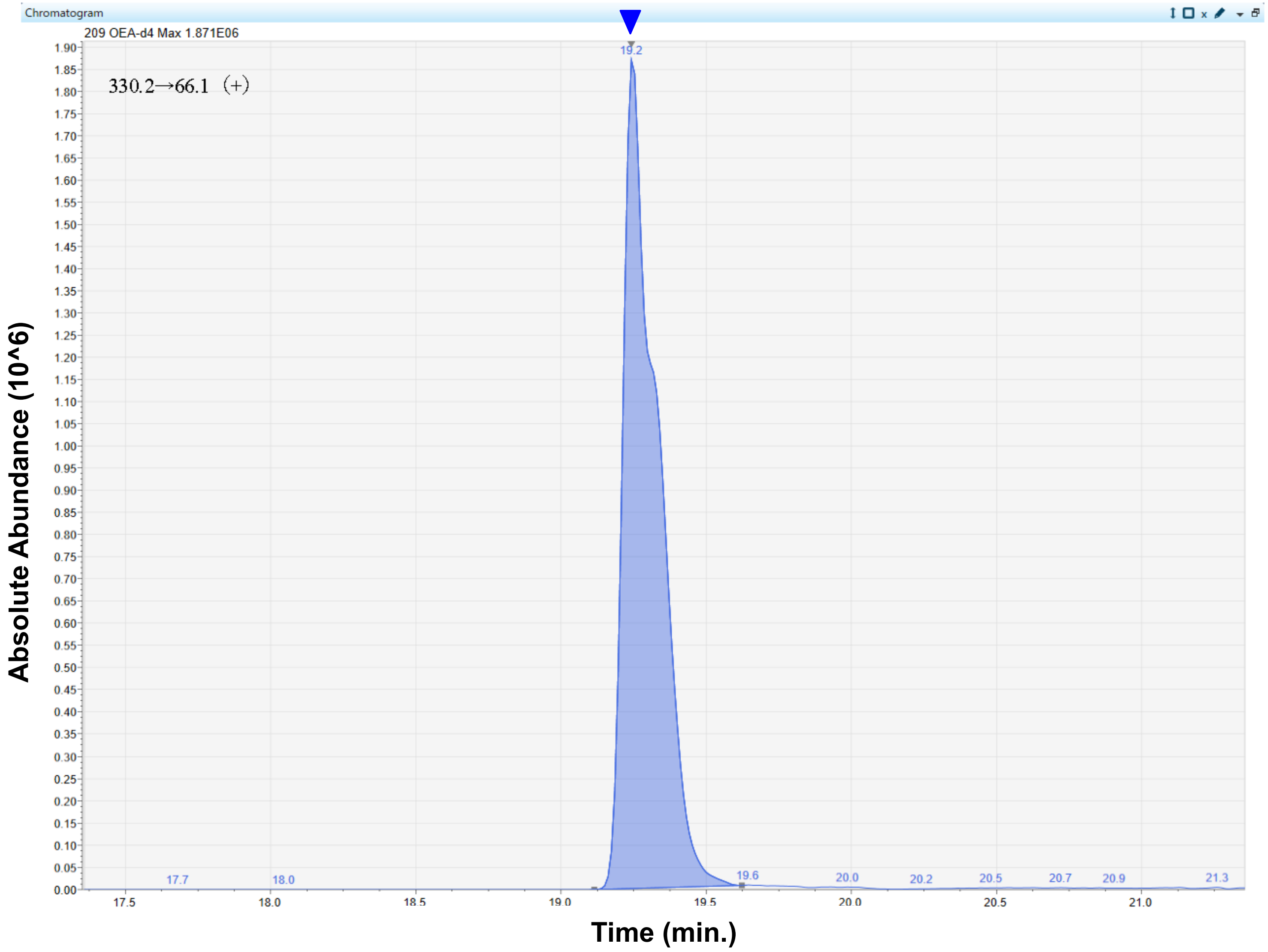

210 OEA

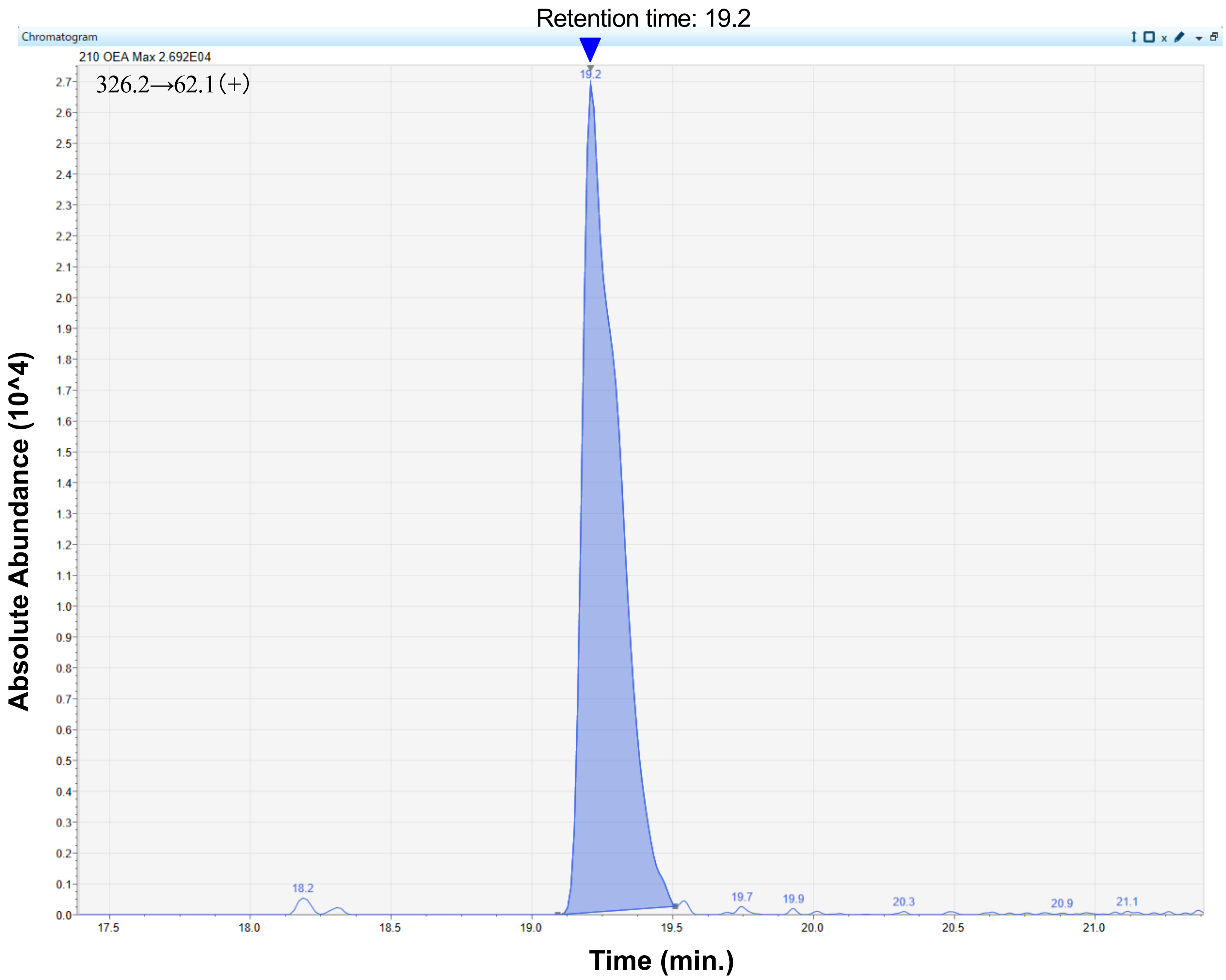
Retention time: 19.2
Chromatogram
210 OEA Max 2.692E04
326.2→62.1 (+)
19.2
18.2
19.7
19.9
20.3
20.9
21.1
Absolute Abundance (10^4)
Time (min.)

212 DHA

Retention time: 20.3

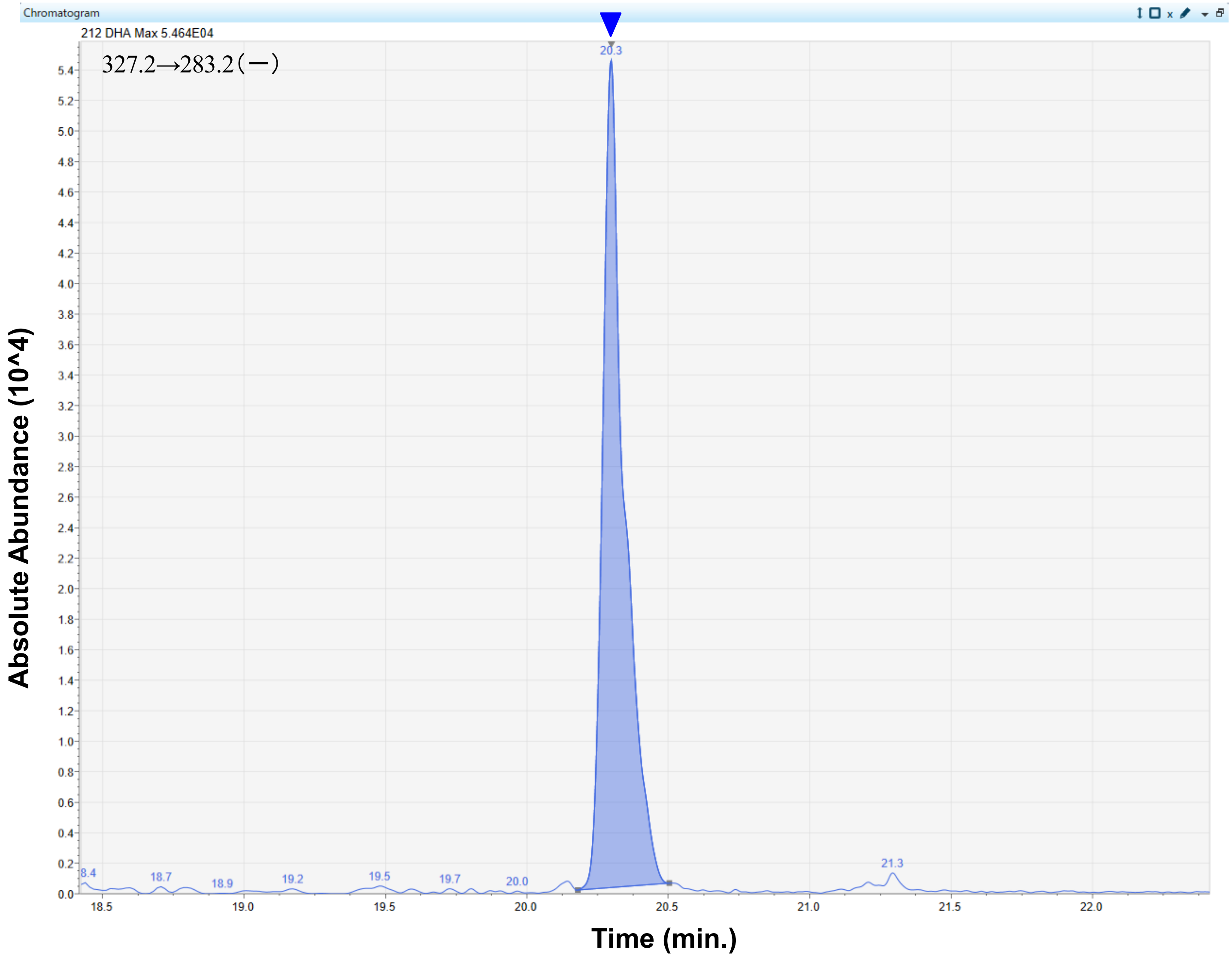

213 AA-d_8 (IS)

Retention time: 20.4

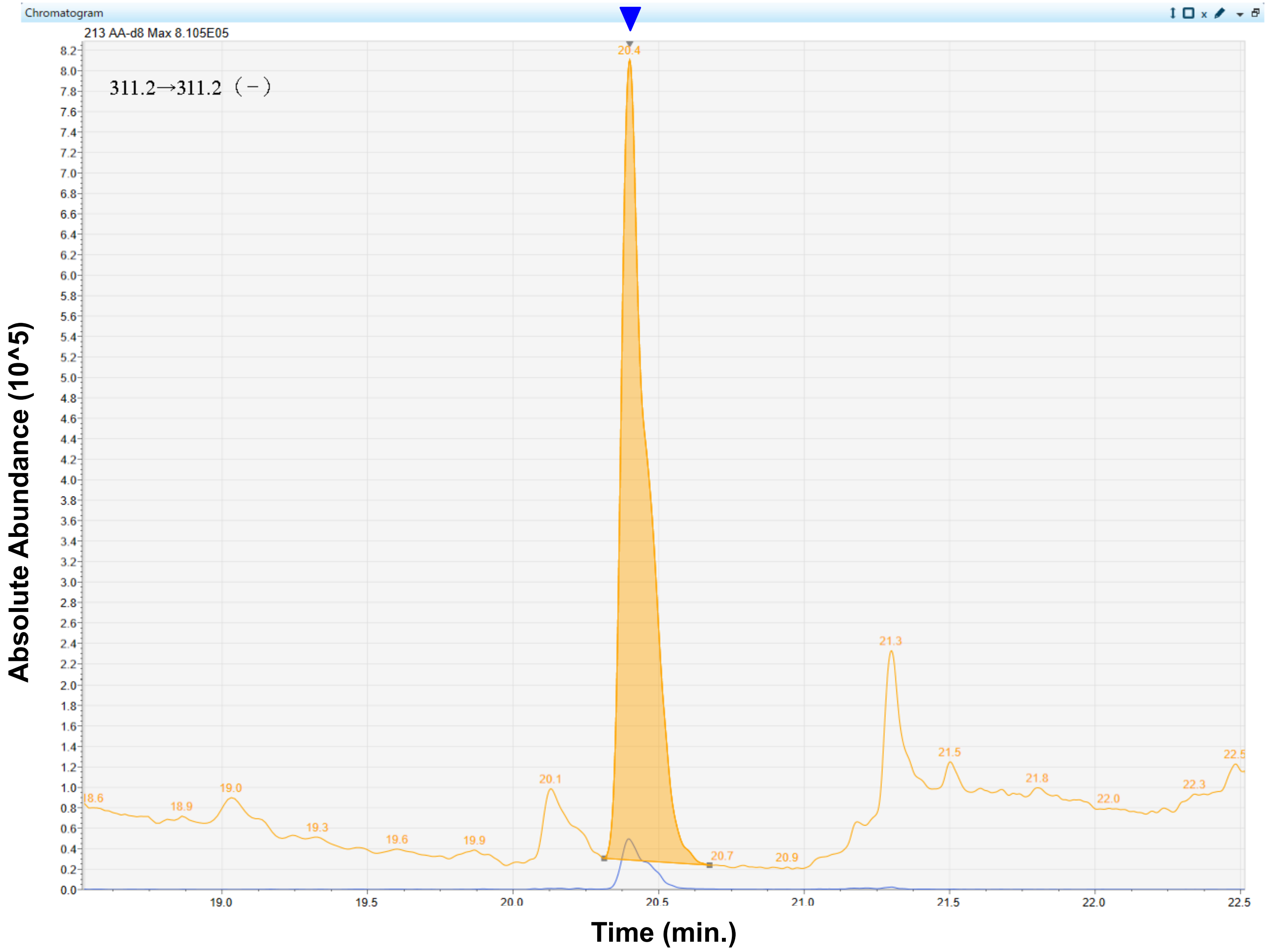

214 AA

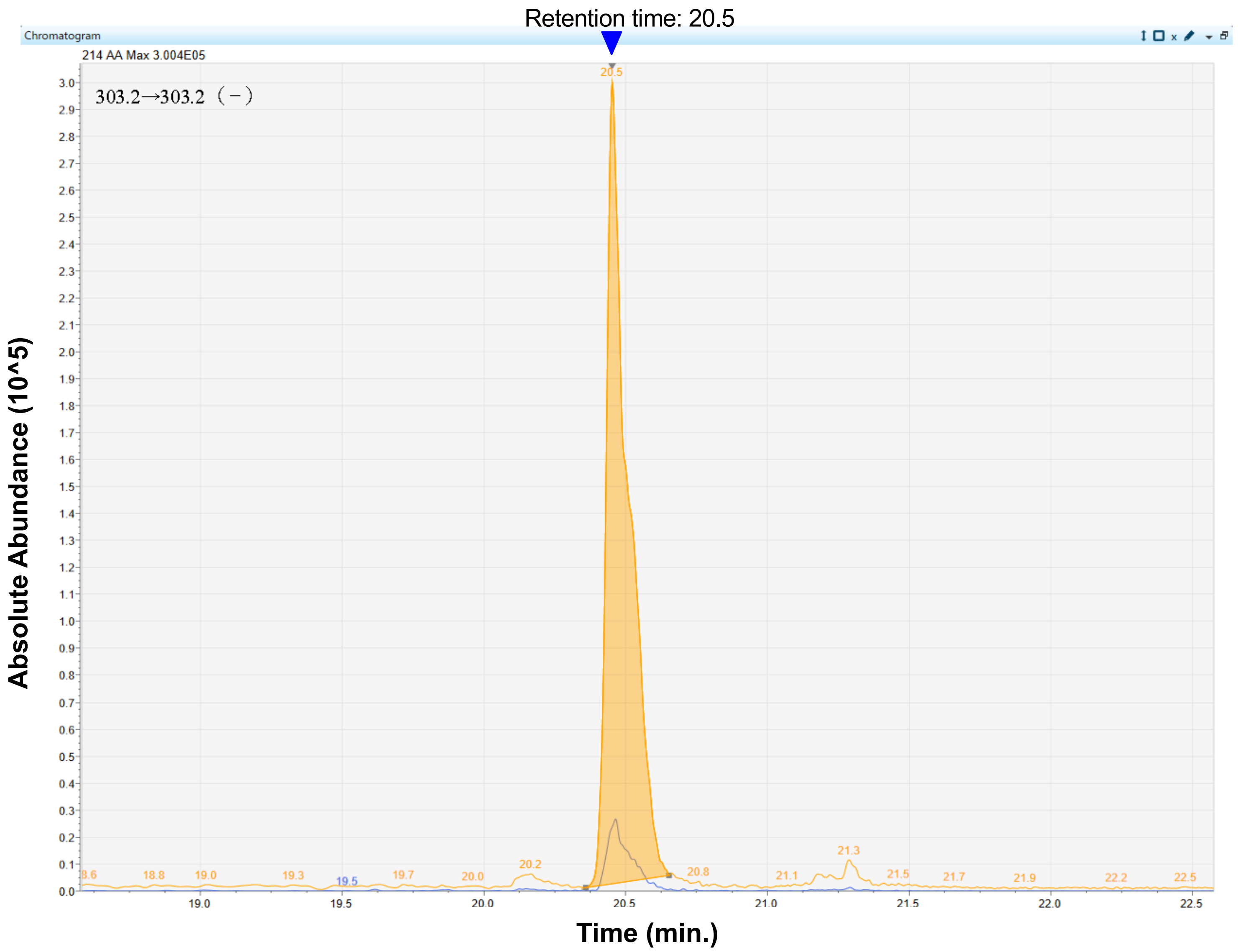
Retention time: 20.5
Chromatogram
214 AA Max 3.004E05
303.2→303.2 (−)
Absolute Abundance (10^5)
Time (min.)
