# Supplementary material for: The lysophospholipase D enzyme Gdpd3 is required to maintain chronic myelogenous leukaemia stem cells
Source: Nat Commun. 2020 Sep 17;11:4681. doi: 10.1038/s41467-020-18491-9 (PMC7499193; doi:10.1038/s41467-020-18491-9)
Supplement: Supplementary file 3 — Reporting Summary [file 41467_2020_18491_MOESM3_ESM.pdf]

nature research

Corresponding author(s): Kazuhito Naka

Last updated by author(s): Aug 25, 2020

Reporting Summary

Nature Research wishes to improve the reproducibility of the work that we publish. This form provides structure for consistency and transparency in reporting. For further information on Nature Research policies, see our Editorial Policies and the Editorial Policy Checklist.

Statistics

For all statistical analyses, confirm that the following items are present in the figure legend, table legend, main text, or Methods section.

n/a	Confirmed	
☐	☒	The exact sample size (*n*) for each experimental group/condition, given as a discrete number and unit of measurement
☐	☒	A statement on whether measurements were taken from distinct samples or whether the same sample was measured repeatedly
☐	☒	The statistical test(s) used AND whether they are one- or two-sided<br>*Only common tests should be described solely by name; describe more complex techniques in the Methods section.*
☒	☐	A description of all covariates tested
☐	☒	A description of any assumptions or corrections, such as tests of normality and adjustment for multiple comparisons
☐	☒	A full description of the statistical parameters including central tendency (e.g. means) or other basic estimates (e.g. regression coefficient) AND variation (e.g. standard deviation) or associated estimates of uncertainty (e.g. confidence intervals)
☐	☒	For null hypothesis testing, the test statistic (e.g. *F*, *t*, *r*) with confidence intervals, effect sizes, degrees of freedom and *P* value noted<br>*Give P values as exact values whenever suitable.*
☒	☐	For Bayesian analysis, information on the choice of priors and Markov chain Monte Carlo settings
☒	☐	For hierarchical and complex designs, identification of the appropriate level for tests and full reporting of outcomes
☒	☐	Estimates of effect sizes (e.g. Cohen's *d*, Pearson's *r*), indicating how they were calculated

Our web collection on statistics for biologists contains articles on many of the points above.

Software and code

Policy information about availability of computer code

Data collection	For lipidomics, LPAs were measured using a NexeraX2 system (Shimadzu Corporation, Kyoto, Japan) and Triple Quad 5500 (Sciex, Framingham, MA, USA). Lipid mediators were measured using a NexeraX2 System and an LCMS-8050 liquid chromatograph mass spectrometer system (Shimadzu). Data were compiled using the Lipidomediator LC/MS/MS Method Package (Ver. 3) (Shimadzu Corporation).<br>Lipidomics data analyses were performed using Traverse MS Ver. 1.2.7. (Reifycs Inc., Tokyo, Japan).<br><br>For RNA-Seq, paired-end reads of 150 bases were generated using HiSeq X Ten (Illumina). Filtered reads were mapped to the reference genome related to the species using STAR v.2.6.1a alignment software. Gene expression levels were measured with DESeq2 v.1.20.0. (https://bioconductor.org/packages/release/bioc/html/DESeq2.html) using the Ensembl database (https://ensembl.org/index.html).<br>MA-plots were created using the Bokeh library (ver. 0.13.0). GO enrichment analyses were performed using the DAVID Bioinformatics Resource 6.8.<br><br>Flowcytometory data were obtained by FACS Aria III instrument (BD Biosciences) with BD FACSDiva software ver.6.1.3 (BD Biosciences).<br>BCR-ABL1/EGFP+ cells were evaluated using a FACS Aria III instrument and the t-SNE algorithm in FlowJoTM (build number 10.6.1) software.<br><br>Fluorescent images were acquired using confocal microscopy (FV10i, Olympus Corporation, Tokyo, Japan) and Photoshop software (CS4 Ver11.0.2. Adobe).
Data analysis	Lipidomics data analyses were performed using Traverse MS Ver. 1.2.7. (Reifycs Inc., Tokyo, Japan).<br>EGFP/BCR-ABL1+ cells in peripheral blood were evaluated using a FACS Aria III instrument and the t-SNE algorithm in FlowJoTM (build number 10.6.1) software.<br>For RNA-Seq analyses, filtered reads were mapped to the reference genome related to the species using STAR v.2.6.1a alignment software.<br>Gene expression levels were measured with DESeq2 v.1.20.0. (https://bioconductor.org/packages/release/bioc/html/DESeq2.html) using the

Ensembl database (https://ensembl.org/index.html).
MA-plots were created using the Bokeh library (ver. 0.13.0) (https://docs.bokeh.org/en/0.13.0/). GO enrichment analyses were performed using the DAVID Bioinformatics Resource 6.8. (http://david.abcc.ncifcrf.gov).
Fluorescent images were acquired using confocal microscopy (FV10i, Olympus) and Photoshop software (CS4 Ver11.0.2. Adobe).
Statistical differences were determined using the log-rank non-parametric test for survival curves (IBM SPSS Statics 23, IBM, Chicago, IL, USA).
Microsoft® Excel (Ver. 16.40, Redmond, WA, USA) were used for P-values on the unpaired one-sided and unpaired two-sided Student's t-test.

For manuscripts utilizing custom algorithms or software that are central to the research but not yet described in published literature, software must be made available to editors and reviewers. We strongly encourage code deposition in a community repository (e.g. GitHub). See the Nature Research guidelines for submitting code & software for further information.

Data

Policy information about availability of data

All manuscripts must include a data availability statement. This statement should provide the following information, where applicable:

- Accession codes, unique identifiers, or web links for publicly available datasets
- A list of figures that have associated raw data
- A description of any restrictions on data availability

For Lipidomics data in Fig. 3a,b and Fig. 4a-c, the original data are available from Source Data file, and Supplementary Method1, 2, 3.
For RNA sequencing data in Fig. 7a, and Supplementary Figure 1, 11a-c, our data are available from a public database gene expression omnibus (GEO, ID: GSE70031 and GSE149442, NCBI, NIH, USA) (https://www.ncbi.nlm.nih.gov/gds/) and Source Data file.
Gene expression levels were measured with the bioconductor package DESeq2 v.1.20.0 (https://bioconductor.org/packages/release/bioc/html/DESeq2.html) using the Ensembl database (https://ensembl.org/index.html).
MA-plots were created using the Bokeh library (ver. 0.13.0) (https://docs.bokeh.org/en/0.13.0/).
GO enrichment analyses were performed using the DAVID Bioinformatics Resource 6.8. (http://david.abcc.ncifcrf.gov).
Gene expression data for Fig. 6d was downloaded from the public microarray dataset in the GEO database under accession code GSE12211 (https://www.ncbi.nlm.nih.gov/gds/).
The source data for Figs. 1b-g, 2a-f, 3a,b, 4a-c, 6a-g, 7a-g, Supplementary Figs. 1, 3b,c, 4a-c, 5a,b, 6a-d, 7, 11a-c, 13a,b, and Supplementary Table 1 have been provided as Source Data file.

Field-specific reporting

Please select the one below that is the best fit for your research. If you are not sure, read the appropriate sections before making your selection.

☒ Life sciences ☐ Behavioural & social sciences ☐ Ecological, evolutionary & environmental sciences

For a reference copy of the document with all sections, see nature.com/documents/nr-reporting-summary-flat.pdf

Life sciences study design

All studies must disclose on these points even when the disclosure is negative.

Sample size

We did not calculate sample size of mice before transplantation of CML stem cells into recipient mice in our experiments.
For first-round transplantation in Fig.2a, Fig. 6a, Fig.7e, normal haematopoietic LSK cells isolated from wild-type and Gdpd3 KO, or wild-type and Lgr4 Gt/Gt mice were transduced with BCR-ABL1 oncogene, and approximately same numbers of BCR-ABL1-transduced LSK cells were transplanted into 5 to 8 recipient mice to compare the survival rate of CML-affected mice on the transplantation. The number of recipient mice transplanted with the LSK cells were different depend on the LSK cell number obtained from each mouse. Thus, different number of recipient mice transplanted with CML LSK cells were compared depend on the normal LSK cell number obtained. To reduce the mouse number, we examined minimal number of recipient mice on each experiment, and repeated transplantation experiments to examine whether or not we could establish statistical significance.

For second-round serial transplantation in Fig.2b, we isolated CML LSK cells from retro-CML mouse model. There was individual difference on the cell number of CML stem cells in each CML-affected mouse. (The maximum numbers of CML LSK cells were sorted from both wild-type and Gdpd3 KO retro-CML mice, and same number of these CML LSK cells were transplanted into recipient mice.) Thus, different number of recipient mice transplanted with CML LSK cells were compared depend on the CML stem cell number obtained. We repeated minimal number of transplantation experiments to examine whether or not we could establish statistical significance.

For the survival experiment of tet-CML mouse model in Fig.1g, the minimal number of mice was compared to examine whether or not we could establish statistical significance from wild type and Gdpd3-deficient tet-CML cohorts that were born.

Data exclusions

If any recipient mouse that were transplanted with CML stem cells passed away within 10 days after irradiation due to acute radiation effects or technical failure of transplantation, we exclude the mouse from the cohort.

Replication

We repeated twice or three times experiments to obtain enough sample size and to confirm the results.

Randomization

For the first- and second-round transplantations in Fig.2a-h, Fig.6a-c, and Fig.7e-g, and Supplementary Figs.5a,b, 6a-d, 7, 9, 13b, recipient C57BL/6 and C57BL/6-CD45.1 mice were randomly allocated after one week of purchase, and were transplanted with normal and CML LSK cells. The normal and CML LSK cells were sorted from the age and sex-matched donor mice (i.e., wild-type, Gdpd3 KO, or Lgr4 Gt/Gt mice).
For the experiments of normal and tet-CML mouse model in Fig.1b, d-g, Fig.3a,b, Fig.4a-c, Fig.5a-c, Fig.7a, c,d, Fig.8a,b, Supplementary Figs. 2a,b, 3b,c, 4a-c, 8a-c, 11a-c, 13a, 14, 15, cells were isolated from the age and sex-matched mice (i.e., wild-type, Gdpd3 KO, or Lgr4 Gt/Gt

	mice).
Blinding	Although results were validated in biological replicates, there were no studies in which investigators were blinded.

Reporting for specific materials, systems and methods

We require information from authors about some types of materials, experimental systems and methods used in many studies. Here, indicate whether each material, system or method listed is relevant to your study. If you are not sure if a list item applies to your research, read the appropriate section before selecting a response.

Materials & experimental systems

n/a	Involved in the study
☐	☒ Antibodies
☐	☒ Eukaryotic cell lines
☒	☐ Palaeontology and archaeology
☐	☒ Animals and other organisms
☐	☒ Human research participants
☒	☐ Clinical data
☒	☐ Dual use research of concern

Methods

n/a	Involved in the study
☒	☐ ChIP-seq
☐	☒ Flow cytometry
☒	☐ MRI-based neuroimaging

Antibodies

Antibodies used

anti-FcγIII/II receptor (Clone # 2.4G2) (Cat. # BD 553142, Lot # 79813, BD Biosciences, dilution 1:200),
anti-CD4 (Clone # RM4-5)-FITC (Cat. # 11-0042-86, Lot #E00084-1631, eBioscience, dilution 1:200),
anti-CD8a (Clone # 53-6.7)-FITC (Cat. # 11-0081-86, Lot # E00117-1632, eBioscience, dilution 1:200),
anti-B220 (Clone # RA3-6B2)-FITC (Cat. # 11-0452-86, Lot #E00310-1631, eBioscience, dilution 1:200),
anti-Mac1 (Clone # M1/70)-FITC (Cat. #11-5931-86, Lot # E00740-1631, eBioscience, dilution 1:200),
anti-Gr-1 (Clone # RB6-8C5)-FITC (Cat. #11-0112-86, Lot #E00150-1632, eBioscience, dilution 1:200),
anti-TER119 (Clone # Ly-76)-FITC (Cat. #11-5921-85, Lot #E00736-1630, eBioscience, dilution 1:200),
anti-Sca-1 (Clone # E13-161.7)-PE (Cat. # BD553336, Lot # 05152, BD Biosciences, dilution 1:200),
anti-cKit (Clone # ACK2)-APC (Cat. # 17-1172-83, Lot # E17176-101, eBioscience, dilution 1:200),
anti-CD135/Flk2/Flt3 (Clone # A2F10)-biotin (Cat. # 13-1351-85, Lot # E02732-1630, eBioscience, dilution 1:200),
anti-CD48 (Clone # HM48-1)-APC-Cy7 (Cat. # 103432, Lot # B173122, BioLegend, dilution 1:250),
anti-CD150/SLAM (Clone # TC15-12F12.2)-Pacific blue (Cat. # 115924, Lot # B224451, BioLegend, dilution 1:250)
Streptavidin-PE-Cy7 (Cat. # 557598, Lot # 6112577, BD Biosciences, dilution 1:400),
anti-CD4 (Clone # RM4-5)-biotin (Cat. # 13-0042-86, Lot #E02364-369, eBioscience, dilution 1:200),
anti-CD8a (Clone # 53-6.7)-biotin (Cat. # 13-0081-86, Lot #E02387-339, eBioscience, dilution 1:200),
anti-B220 (Clone # RA3-6B2)-biotin (Cat. # 13-0452-86, Lot #E02532-301, eBioscience, dilution 1:200),
anti-Mac1 (Clone # M1/70)-biotin (Cat. #13-0112-86, Lot #E033770, eBioscience, dilution 1:200),
anti-Gr-1 (Clone # RB6-8C5)-biotin (Cat. #13-5931-86, Lot # E033865, eBioscience, dilution 1:200),
anti-TER119 (Clone # Ly-76)-biotin (Cat. #13-5921-85, Lot # 4300555, eBioscience, dilution 1:200),
anti-CD4 (Clone # RM4-5)-PE-Cy7 (Cat. # 25-0042-82, Lot #E07503-1630, eBioscience, dilution 1:500),
anti-CD8a (Clone # 53-6.7)-PE-Cy7 (Cat. # 25-0081-82, Lot # E07510-1631, eBioscience, dilution 1:500),
anti-B220 (Clone # RA3-6B2)-PE (Cat. # 553089, Lot # 76923, BD Biosciences, dilution 1:500),
anti-Mac1 (Clone # M1/70)-APC (Cat. # 17-0112-82, Lot #E07073-1631, eBioscience, dilution 1:500),
anti-Gr-1 (Clone # RB6-8C5)-APC (Cat. # 17-5931-82, Lot # E07334-1630, eBioscience, dilution 1:500),
anti-CD45.2-FITC (Clone # 104, Cat. # 11-0454-85, Lot # E00316-130, eBioscience, dilution 1:200),
anti-CD45.1-PE (Clone # A20, Cat. # BD553776, Lot # 17194, BD Biosciences, dilution 1:200),
anti-CD34-APC (Clone # 8G12) (Cat. # 340441, Lot # 6183704, BD Biosciences, dilution 1:100),
anti-BrdU-FITC antibody (Cat. # 51-23614L, Clone # 3D4, Lot # 7222635, BD Biosciences. dilution 1:500) in FITC BrdU Flow Kit (Cat. # 559619, BD PharmingenTM),
anti-phospho-AKT (Ser473) (Cat. # 4060S, Lot# 5, Clone # D9E, Cell Signaling Technology, dilution 1:50),
anti-phospho-S6 ribosomal protein (Ser235/236) (Cat. # 4858S, Lot# 11, Clone # D57.2.2E, Cell Signaling Technology, dilution 1:50),
anti-Ki-67 (Cat. # 550609, Lot# 43365, Clone # B56, BD Pharmingen, dilution 1:50),
AlexaFluor 546-conjugated goat anti-mouse IgG (Cat. # A11030, Lot # 833292, Molecular Probes®, dilution 1:200),
AlexaFluor 647-conjugated goat anti-rabbit IgG (Cat. # A21245, Lot # 927083, Molecular Probes®, dilution 1:200),
anti-Foxo3a (Cat. #2497S, Lot # 2, Clone 75D8; Cell Signaling Technology, dilution 1:25),
anti-active β-catenin (Cat. # 05-665, Lot #, 2700799 and 3270747, Clone 8E7; Millipore, dilution 1:25),
Duolink in situ PLA starter set RED (Merck)

Validation

We complied previous reports as followed; Naka et al., Nature 463, 676-680, 2010., Reynaud et al., Cancer Cell, 20, 661-673, 2011, and Naka et al., Nature Communications, 6, 8039, 2015.

Eukaryotic cell lines

Policy information about cell lines

Cell line source(s)	OP-9 murine stromal cells were purchased from ATCC® (CRL-2749, Lot # 70019055).<br>K562 CML cells were purchased from ATCC® (CCL-243, Lot # 70016362).
Authentication	OP-9 murine stromal cells and K562 human CML cells were authenticated.
Mycoplasma contamination	Cell lines were not tested for mycoplasma contamination.
Commonly misidentified lines<br>(See ICLAC register)	This study does not contain samples collected from fields.

Animals and other organisms

Policy information about studies involving animals; ARRIVE guidelines recommended for reporting animal research

Laboratory animals	The Gdpd3 disrupted mouse (C57BL/6 background) was established in Setsurotech Inc. (Tokushima, Japan).<br>For supplementary table 1, female and male Gdpd3 disrupted mice (10 and 39 wk-old) were used.<br>For Fig.1c, supplementary figs.3b,c, 4a-c,5a,b, female and male mice (6-8 wk-old) were used.<br>The Lgr4/Gpr48 gene-trap mice were backcrossed for four generations in the C57BL/6 background.<br>Female and male mice (6-8 wk-old) were used in this study.<br>C57BL/6 mice were purchased from Crea Japan, Inc. (Tokyo, Japan).<br>For supplementary table 1, female and male C57BL/6 mice (10 wk-old) were used.<br>For RNA-sequencing, qRT-PCR, and lipidmics in Fig.1b, Fig.3a,b, Fig.4a-c, and supplementary Figs.1, female and male C57BL/6 mice (10-12 wk-old) were used as control.<br>For bone marrow transplantation (BMT) of CML-LSK cells in Fig.2a-h, Fig.6a-c, and Fig.7e-g, and Supplementary Figs. 6a-d, 7, 9, 13b, female C57BL/6 mice (6-8 wk-old) were used for recipient mice and for isolation of competitor BMMNCs.<br>SCL-tTA transgenic mice (JAX database strain #006209) and TRE-BCR-ABL1 transgenic mice (JAX database strain #006202) were purchased from the Jackson Laboratory. SCL-tTA (C57BL/6; F5) and TRE-BCR-ABL1 (C57BL/6; F5) transgenic mice were used in this study.<br>For Fig.1g, female and male tet-CML-affected mice (Gdpd3 wild-type, Gdpd3 KO, 10-53 wk-old) were used in the survival experiments.<br>For Fig.1b,c,e-g, Fig.3a,b, Fig.4a-c, Fig.5a-c, Fig.7a,b,c,d, Fig.8a,b, Supplementary Figs. 2a,b, 3b,c, 4a-c, 8a-c, 10, 11a-c, 12, 13a, 14, 15, female and male tet-CML-affected mice (Gdpd3 wild-type, Gdpd3 KO, and Lgr4/Gpr48Gt/Gt, 10-12 wk-old) were used.<br>C57BL/6-CD45.1 mouse strain was purchased from Sankyo Labo Service Corporation, Inc. (Tokyo, Japan).<br>Female and male C57BL/6-CD45.1 mice (6-8 wk-old) were used for first- and second-rounds serial transplantation of normal LSK cells in supplementary figure 5a,b.<br>The mice were housed in a 12-h light:dark cycle at 25°C±2°C temperature with relative humidity of 50±20 %, and given ad libitum access to water and feed. Mice were maintained in SPF (specific-pathogen-free) conditions.
Wild animals	We did not use wild animals in this study.
Field-collected samples	We did not use filed-collected samples in this study.
Ethics oversight	All animal care and experimentation were carried out in accordance with the guidelines for animal and recombinant DNA experiments of Hiroshima University (Authorized Protocol Numbers A18-36, A18-37 and 30-257) and Fundamental Guidelines for Proper Conduct of Animal Experiment and Related Activities in Academic Research Institutions under the jurisdiction of the Ministry of Education, Culture, Sports, Science and Technology Japan.<br>The latest inspection by the Japanese Association for Laboratory Animal Sciences was at December 5th, 2017.

Note that full information on the approval of the study protocol must also be provided in the manuscript.

Human research participants

Policy information about studies involving human research participants

Population characteristics	The viable BMMNCs were collected from a primary CML patient for diagnosis. The patient was confirmed diagnosis of chronic phase CML. The BMMNCs were isolated from a surplus of bone marrow fluid without any additional bone marrow aspiration to the patient, and the anonymized BMMNCs were stored in freezing condition.
Recruitment	The BMMNCs from a primary CML patient for diagnosis at the University Hospital, Dokkyo Medical University (Tochigi, Japan) were collected between October 2014 and March 2019.
Ethics oversight	Institutional Review Board (IRB) of the University Hospital, Dokkyo Medical University (Tochigi, Japan) (IRB approval number: 26058). All procedures involving human participants were performed in compliance with the relevant ethical standards.

Note that full information on the approval of the study protocol must also be provided in the manuscript.

Flow Cytometry

Plots

Confirm that:

- [x] The axis labels state the marker and fluorochrome used (e.g. CD4-FITC).
- [x] The axis scales are clearly visible. Include numbers along axes only for bottom left plot of group (a 'group' is an analysis of identical markers).
- [x] All plots are contour plots with outliers or pseudocolor plots.
- [x] A numerical value for number of cells or percentage (with statistics) is provided.

Methodology

Sample preparation	For isolation of the most primitive long-term (LT) CML stem cells, bone marrow mononuclear cells (BMMNCs) were isolated from the two hind limbs of tet-CML-affected mice (SCL-tTA+ TRE-BCR-ABL1+) and healthy littermate mice (SCL-tTA+) at five weeks after Dox withdrawal. To purify LT-CML stem cells, BMMNCs were first blocked by incubation with anti-FcγIII/II receptor monoclonal antibody (mAb) (dilution 1:200, Clone # 2.4G2, Cat. # BD 553142, Lot # 79813, BD Biosciences), and then stained with anti-CD4-FITC (dilution 1:200, Clone # RM4-5, Cat. # 11-0042-86, Lot #E00084-1631, eBioscience), anti-CD8a-FITC (dilution 1:200, Clone # 53-6.7, Cat. # 11-0081-86, Lot # E00117-1632, eBioscience), anti-B220-FITC (dilution 1:200, Clone # RA3-6B2, Cat. # 11-0452-86, Lot #E00310-1631, eBioscience), anti-Mac1-FITC (dilution 1:200, Clone # M1/70, Cat. #11-5931-86, Lot # E00740-1631, eBioscience), anti-Gr-1-FITC (dilution 1:200, Clone # RB6-8C5, Cat. #11-0112-86, Lot #E00150-1632, eBioscience), anti-TER119-FITC (dilution 1:200, Clone # Ly-76, Cat. #11-5921-85, Lot #E00736-1630, eBioscience), anti-Sca-1-PE (dilution 1:200, Clone # E13-161.7, Cat. # BD553336, Lot # 05152, BD Biosciences), anti-cKit-APC (dilution 1:200, Clone # ACK2, Cat. # 17-1172-83, Lot # E17176-101, eBioscience), anti-CD135/Flk2/Flt3-biotin (dilution 1:200, Clone # A2F10, Cat. # 13-1351-85, Lot # E02732-1630, eBioscience), anti-CD48-APC-Cy7 (dilution 1:250, Clone # HM48-1, Cat. # 103432, Lot # B173122, BioLegend), and anti-CD150/SLAM-Pacific blue (dilution 1:250, Clone # TC15-12F12.2, Cat. # 115924, Lot # B224451, BioLegend) mAbs. Biotinylated primary mAbs were visualised using Streptavidin-PE-Cy7 (dilution 1:400, Cat. # 557598, Lot # 6112577, BD Biosciences). CD150+CD48-CD135-LSK (Lineage-Sca-1+cKit+) cells were purified using a FACS Aria III cell sorter (S/N, P64828201002) with BD FACSDiva software ver.6.1.3 (BD Biosciences).<br><br>To purify WT haematopoietic stem/progenitor (LSK) cells, BMMNCs isolated from WT, Gdpd3-/-, and Lgr4Gt/Gt mice (6-8 wk-old) were first blocked by incubation with anti-FcγIII/II receptor mAb (dilution 1:200, Clone # 2.4G2, Cat. # BD 553142, Lot # 79813, BD Biosciences), and then stained with anti-CD4-FITC (dilution 1:200, Clone # RM4-5, Cat. # 11-0042-86, Lot #E00084-1631, eBioscience), anti-CD8a-FITC (dilution 1:200, Clone # 53-6.7, Cat. # 11-0081-86, Lot # E00117-1632, eBioscience), anti-B220-FITC (dilution 1:200, Clone # RA3-6B2, Cat. # 11-0452-86, Lot #E00310-1631, eBioscience), anti-Mac1-FITC (dilution 1:200, Clone # M1/70, Cat. #11-5931-86, Lot # E00740-1631, eBioscience), anti-Gr-1-FITC (dilution 1:200, Clone # RB6-8C5, Cat. #11-0112-86, Lot #E00150-1632, eBioscience), anti-TER119-FITC (dilution 1:200, Clone # Ly-76, Cat. #11-5921-85, Lot #E00736-1630, eBioscience), anti-Sca-1-PE (dilution 1:200, Clone # E13-161.7, Cat. # BD553336, Lot # 05152, BD Biosciences), and anti-cKit-APC (dilution 1:200, Clone # ACK2, Cat. # 1 (Clone # ACK2)7-1172-83, Lot # E17176-101, eBioscience). WT LSK cells were purified using a FACS Aria III cell sorter (BD Biosciences).<br><br>For serial transplantation of CML-LSK cells, mononuclear cells isolated from the two hind limbs and spleen of retro-CML-affected mice were first blocked by incubation with anti-FcγIII/II receptor mAb (dilution 1:200, Clone # 2.4G2, Cat. # BD 553142, Lot # 79813, BD Biosciences), and then stained with anti-CD4-biotin (dilution 1:200, Clone # RM4-5, Cat. # 13-0042-86, Lot #E02364-369, eBioscience), anti-CD8a-biotin (dilution 1:200, Clone # 53-6.7, Cat. # 13-0081-86, Lot #E02387-339, eBioscience), anti-B220-biotin (dilution 1:200, Clone # RA3-6B2, Cat. # 13-0452-86, Lot #E02532-301, eBioscience), anti-Mac1-biotin (dilution 1:200, Clone # M1/70, Cat. #13-0112-86, Lot #E033770, eBioscience), anti-Gr-1-biotin (dilution 1:200, Clone # RB6-8C5, Cat. #13-5931-86, Lot # E033865, eBioscience), anti-TER119-biotin (dilution 1:200, Clone # Ly-76, Cat. #13-5921-85, Lot # 4300555, eBioscience), anti-Sca-1-PE (dilution 1:200, Clone # E13-161.7, Cat. # BD553336, Lot # 05152, BD Biosciences), and anti-cKit-APC (dilution 1:200, Clone # ACK2, Cat. # 17-1172-83, Lot # E17176-101, eBioscience) mAbs. Biotinylated primary mAbs were visualised using Streptavidin-PE-Cy7 (dilution 1:400, Cat. # 557598, Lot # 6112577, BD Biosciences). BCR-ABL1/EGFP+ CML-LSK cells were purified using a FACS Aria III cell sorter (BD Biosciences).
Instrument	We used a FACS Aria III cell sorter (S/N, P64828201002) (BD Biosciences).
Software	We used BD FACSDiva software ver.6.1.3 (BD Biosciences).
Cell population abundance	We did not re-confirm the post-sorted fractions.
Gating strategy	We complied previous reports as followed; Naka et al., Nature 463, 676-680, 2010., Reynaud et al., Cancer Cell, 20, 661-673, 2011, and Naka et al., Nature Communications, 6, 8039, 2015.<br>We harvested bone marrow mononuclear cells (BMMNCs) from littermate healthy control mice (SCL-tTA+) and tet-CML mice (SCL-tTA+TRE-BCR-ABL1+), and isolated LT-stem cells (CD150+CD48-CD135-LSK), CD48+LSK cells, multipotent progenitors (MPP), and LK (Lineage-Sca-1-cKit+) populations as indicated in Supplementary Fig.2.

- [x] Tick this box to confirm that a figure exemplifying the gating strategy is provided in the Supplementary Information.
